# Rising dust pollution across Europe in a changing climate

**DOI:** 10.1038/s41586-026-10743-w

**Published:** 2026-07-15

**Authors:** Petros N. Vasilakos, Abhishek Upadhyay, Manousos I. Manousakas, Andrés Alastuey, James D. Allan, Célia A. Alves, Benjamin Bergmans, Benjamin T. Brem, Sonia Castillo, Theodoros Christoudias, Cristina Colombi, Sébastien Conil, Katja Dzepina, Anja Eichler, Konstantinos Eleftheriadis, Olivier Favez, Michael Flynn, Kristina Glojek, Stuart K. Grange, David C. Green, Christoph Hueglin, Jean-Luc Jaffrezo, Theo M. Jenk, Jianhui Jiang, Ekaterina Krymova, Franco Lucarelli, Petra Makorič, Dario Massabò, Nikolaos Mihalopoulos, Griša Močnik, Robin L. Modini, Claudia Mohr, Attilio Naccarato, Petra Pokorná, Paolo Prati, Nicole Probst-Hensch, André S. H. Prévôt, Xavier Querol, Cristina Reche, Jesús D. de la Rosa, Mark M. Scerri, Jean Sciare, Michael Sigl, Anja H. Tremper, Rita Traversi, Daniel Trejo Banos, Maria Tsagkaraki, Gaëlle Uzu, Roberta Vecchi, Marta Via, Kees de Hoogh, Imad El-Haddad, Kaspar R. Daellenbach

**Affiliations:** 1 PSI Center for Energy and Environmental Sciences, Paul Scherrer Institute, Villigen, Switzerland; 2 Environmental Radioactivity & Aerosol Technology for Atmospheric and Climate Impact Lab, INRaSTES, National Centre for Scientific Research “Demokritos”, Athens, Greece; 3 Institute of Environmental Assessment and Water Research (IDAEA-CSIC), Barcelona, Spain; 4 Department of Earth and Environmental Sciences, The University of Manchester, Manchester, UK; 5 National Centre for Atmospheric Science, The University of Manchester, Manchester, UK; 6 Department of Environment and Planning, CESAM - Centre for Environmental and Marine Studies, University of Aveiro, Aveiro, Portugal; 7 Institut Scientifique de Service Public - ISSeP, Liège, Belgium; 8 Andalusian Institute for Earth System Research, IISTA-CEAMA, University of Granada, Granada, Spain; 9 Climate and Atmosphere Research Centre (CARE-C), The Cyprus Institute, Nicosia, Cyprus; 10 Agenzia Regionale per la Protezione dell’Ambiente Lombardia (ARPA Lombardia), Milan, Italy; 11 ANDRA DISTEC/EES Observatoire Pérenne de l’Environnement, Bure, France; 12 Centre for Atmospheric Research (CRA), University of Nova Gorica, Ajdovščina, Slovenia; 13 Oeschger Centre for Climate Change Research (OCCR), University of Bern, Bern, Switzerland; 14 Institut National de l’Environnement Industriel et des Risques (INERIS), Verneuil-en-Halatte, France; 15 Laboratory for Air Pollution and Environmental Technology, Swiss Federal Laboratories for Materials Science and Technology (EMPA), Dübendorf, Switzerland; 16 Climate and Environmental Physics, Physics Institute, University of Bern, Bern, Switzerland; 17 Environmental Research Group, School of Public Health, Imperial College London, London, UK; 18 University Grenoble Alpes, CNRS, IRD, INP-G, INRAE, IGE (UMR 5001), Grenoble, France; 19 Global Institute for Urban and Regional Sustainability, School of Ecological and Environmental Sciences, East China Normal University, Shanghai, China; 20 Swiss Data Science Center, EPFL and ETH Zurich, Zurich, Switzerland; 21 Department of Physics and Astronomy, University of Florence and INFN, Florence, Italy; 22 Laboratory for Environmental and Life Sciences, University of Nova Gorica, Nova Gorica, Slovenia; 23 Dipartimento di Fisica, Università di Genova, Genoa, Italy; 24 Environmental Chemical Processes Laboratory, Department of Chemistry, University of Crete, Heraklion, Greece; 25 Institute for Environmental Research and Sustainable Development, National Observatory of Athens, Athens, Greece; 26 Department of Chemistry and Chemical Technologies, University of Calabria, Rende, Italy; 27 Institute of Chemical Process Fundamentals, Czech Academy of Sciences, Prague, Czech Republic; 28 Swiss Tropical and Public Health Institute, Allschwil, Switzerland; 29 University of Basel, Basel, Switzerland; 30 CIQSO-Center for Research in Sustainable Chemistry, Associate Unit CSIC-University of Huelva “Atmospheric Pollution”, Huelva, Spain; 31 Institute of Earth Systems, University of Malta, Msida, Malta; 32 Department of Chemistry “Ugo Schiff”, University of Florence, Florence, Italy; 33 Department of Physics, Università degli Studi di Milano, Milan, Italy

**Keywords:** Climate and Earth system modelling, Environmental impact

## Abstract

Mineral desert dust is a major contributor to total atmospheric particulate matter^1^. Desert dust outbreaks degrade air quality and can pose adverse health effects^2^, including asthma exacerbation^3^ and increased mortality^4^. At some European locations, there has been a rise in the intensity and frequency of transported dust outbreaks from deserts in recent decades^5–9^. However, it remains unclear whether this increase is consistent across Europe and whether desertification and aridity or shifts in atmospheric circulation are the main drivers behind this rise. Here we compile a database of daily dust metal concentrations from European sites, establishing robust elemental ratios for transported dust. Using this database, we develop a machine learning model to estimate daily PM_10_ (particulate matter smaller than 10 μm) dust concentrations from 2012 to 2021, ranging from 2.09 ± 1.05 μg m^−3^ across northern and central Europe to 5.28 ± 2.65 μg m^−3^ across the south. In southern Europe, residents are exposed to transported dust events averaging 9.68 ± 4.85 μg m^−3^, linked to a 0.67 ± 0.02% rise in daily mortality. Intensified dust intrusions over the past decade are linked to shifts in atmospheric circulation. Data from an Alpine ice core record shows a 110% increase in dust concentrations since pre-industrial times, mostly associated with North African desertification. As climate change accelerates land degradation and affects weather patterns, worsening dust pollution may pose increasing risks to public health and air quality goals.

## Abstract (web summary)

A database of daily dust metals in Europe is established and a machine learning model shows that transported dust intrusion events over the past decade are linked to changes in atmospheric circulation, while an ice core shows that the increase in dust since pre-industrial times is governed by increasing aridity in North Africa..

## Main

Dust has a central role in the Earth and climate systems by interacting with solar and terrestrial infrared radiation^10^, serving as cloud condensation and ice nuclei^11^, supplying iron and other nutrients to ecosystems^12^—particularly over oceans—and influencing atmospheric acidity^13^, which affects the partitioning of inorganic and organic vapours^14^. Desert dust outbreaks degrade air quality by raising PM_10_ and PM_2.5_ (particulate matter smaller than 2.5 μm) levels^15,16^, disrupt economic activities such as air traffic^17^ and pose adverse health effects^2^, including asthma exacerbation^3^, stillbirths^18^, increased mortality^4^ and even the transport of pathogens^19^. Analyses of transported dust outbreaks from the Saharan and Middle East deserts, at specific European locations, reveal a rising intensity and frequency in recent decades^5–9^. Regional and global modelling show that dust emissions and transport are tightly coupled to large-scale circulation patterns, including the North Atlantic Oscillation^20–22^ (NAO) and stratospheric intrusions^21^, which would modulate the frequency of dust intrusions over Europe. Long-term reconstructions from Alpine ice cores^23^ and recent assessments of dust-related health risks^2^ further highlight the growing importance of dust under a changing climate, probably linked to increasing North African droughts. However, owing to the strong spatial and temporal variability in dust levels, it remains unclear whether this increase is consistent across Europe and whether desertification and aridity or shifts in atmospheric circulation are the main drivers behind this rise. Most existing studies rely on coarse-resolution dust transport models, satellite proxies or total PM_10_ concentrations^24^ to infer dust concentrations, approaches limited by uncertainties in emission parameterizations, spatial resolution or the lack of observational constraints. Consequently, there is no continent-wide, observation-driven quantification of dust levels, trends, drivers and impacts on air quality and health.

**Fig. 1:**
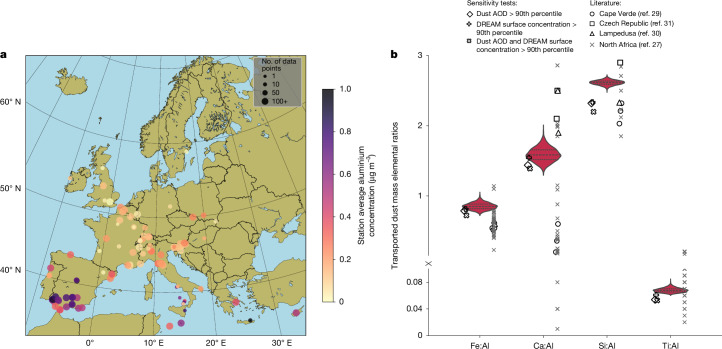
Dust observations and elemental ratios of transported dust in Europe*.* **a**, Average aluminium concentrations for 103 locations in Europe. The size of the dot corresponds to the number of daily measurements and the colour corresponds to the average concentration of Al at this location. High Al concentrations show the large impact of transported dust on air quality in southern Europe. **b**, Violin plots of elemental ratios of transported dust, estimated by means of bootstrapped zero-intercept linear regressions applied to daily data with increased transported dust concentrations (Al concentrations >1 μg m^−3^ for Fe:Al and Ca:Al). The diamonds and outlined crosses and ‘×’s correspond to filtering to the 90th percentile of dust aerosol optical depth (AOD), DREAM dust surface concentration and both, respectively. Circles, squares and triangles represent literature values from Chiapello et al.^29^, Loskot et al.^31^ and Marconi et al.^30^, respectively, for dust arriving to Europe, whereas North Africa source estimates from Liu et al.^27^ are denoted with the grey ‘×’s. Source data

Here we address this gap by developing a data-driven, observation-constrained random forest (RF) model trained on the most extensive elemental dataset available in Europe. The model integrates several data products, including satellite-derived dust optical depth, land use information and a state-of-the-art physical dust model. The resulting European-wide daily dust PM_10_ concentrations for 2012–2021, combined with ice core observations, enable a comprehensive characterization of both the short-term variability and long-term drivers of dust, identifying the climatic modes controlling its transport and assessing its contribution to present air quality limit values and associated mortality from short-term exposure. We compile a uniquely comprehensive database of PM_10_ dust metal measurements (Al, Ti, Si, Ca and Fe) from 103 rural and urban sites across Europe, totalling about 18,500 daily measurements (Fig. 1a and Supplementary Table 1). We note that the spatial coverage of metal measurements is uneven across Europe, with limited data availability in northeastern Europe, the Balkans and Scandinavia—regions that are also climatologically less affected by Saharan dust. This data limitation underscores the need for further long-term supersite measurements in these areas, in line with present European air quality directives^24^. Elements primarily associated with transported dust (Al, Si and Ti) show strong correlations throughout the dataset (Supplementary Fig. 1). By contrast, Ca and Fe, which are also influenced by local emissions such as brake wear, construction, soil erosion and road resuspension, show a larger variation in elemental ratios (Supplementary Fig. 1). Consistent with previous findings^15^ in Barcelona, Al and Ti serve as reliable tracers of desert dust, whereas Ca has substantial urban contributions. Although mean Al concentrations show no clear east–west differences, levels are clearly higher in the south than in the north, reflecting the susceptibility of these regions to Saharan dust intrusions in line with the literature^25^ (Fig. 1a). Although dust is mostly present in the coarse mode, on the basis of the observed PM_2.5_:PM_10_ elemental ratios at locations for which both size fractions are available, we estimate that 27.7% of dust is present as PM_2.5_ (Supplementary Fig. 2).

Next we determine elemental ratios characteristic of transported dust by applying zero-intercept linear regression to a subset of data with high Al concentrations (here >1 μg m^−3^; details in Methods section ‘Uncertainty of elemental ratios and dust estimate’)—indicative of increased transported dust contributions—and find Si:Al, Ti:Al, Ca:Al and Fe:Al ratios of 2.610 ± 0.033, 0.068 ± 0.003, 1.580 ± 0.099 and 0.850 ± 0.052, respectively (Fig. 1b). Although standard linear regression would provide a better fit of the data, it would not be physically consistent, because, for pure dust, if one species was zero, then all would have to be zero and a non-zero-intercept regression violates that principle. These elemental ratios align well with reported values for dust transported to Europe^25–31^ and North Africa source estimates^27^, acknowledging variability in elemental composition depending on the emission area, particularly for Ca. Despite this variability, given the extensive spatial and temporal coverage of our dataset across urban and rural environments, these ratios provide the most comprehensive observational characterization of the average elemental composition of transported dust in Europe so far.

Typically transported dust events, originating mainly from the Saharan and Middle East deserts, are predicted using three-dimensional models such as SKIRON, the Multiscale Online Nonhydrostatic Atmosphere Chemistry model (MONARCH), CHIMERE^32^ and the Barcelona Supercomputing Center Dust Regional Atmospheric Model (BSC-DREAM model, hereafter DREAM)^33^. These models are essential for long-distance transport predictions as they simulate key atmospheric dust processes, including aeolian emission, transport and deposition. Satellite products, particularly dust optical depth at 550 nm (refs. ^11,34^), are also used to refine and validate model predictions. In fact, the European Commission’s working paper proposed a methodology combining modelling, remote sensing and particulate matter concentration measurements to estimate daily dust contributions to particulate matter for compliance with limit values^24^. Although effective at predicting extreme events, these models struggle with accurately resolving ground-level concentrations, often miss minor dust intrusions (<10 μg m^−3^) and do not account for local sources of dust, such as wind-blown soil erosion, resuspension or agricultural activities^35^. Recently, machine learning techniques have shown promise in predicting dust events, particularly in the Middle East^36,37^. However, these models mostly rely on PM_10_ measurements, which include substantial contributions from anthropogenic and naturally formed particles^38^, making them less accurate as dust-specific indicators.

## Dust phenomenology

We developed a RF model for daily, ground-level dust concentrations (10 × 10 km), fusing the unique measurement database with reanalysis dust optical depth, a state-of-the-art physical dust model (DREAM^33^), meteorological parameters (wind speed, temperature, wind direction and total precipitation) and land use data such as road coverage and population density (250 m-resolution land use data were used for each station) over continental Europe.

For quantifying dust, we opted for a single-tracer approach rather than a multitracer one, as most locations lack comprehensive tracer data, preventing consistent training of the RF model. Although Si would be an ideal tracer owing to its clear association with transported dust, Si measurements are notably less abundant than Al owing to common sampling methodologies using quartz filters. Given the strong correlation between Al and Si where both are available, we rely on Al as a transported dust proxy in the RF model (Supplementary Fig. 1). Specifically, we predict aluminium concentrations with the RF model and convert them to dust concentrations using the elemental ratios from the observational dataset (Ti:Al = 0.068, Si:Al = 2.610, Fe:Al = 0.850, Ca:Al = 1.580; Fig. 1b). This conversion assumes the elements being present as oxides, that is, Al_2_O_3_, SiO_2_, CaO, Fe_2_O_3_, FeO, TiO_2_ and K_2_O (2.20Al + 2.49Si + 1.63Ca + 1.94Ti + 2.42Fe)^39^, which yields a dust estimate 13.5 times the Al concentration, similar to values reported in the literature^26^. We validate the RF model by predicting daily Al levels at locations and years unseen by the model, using a rigorous leave-one-station-out (training the model on all but one station and testing it on the excluded station) and leave-one-year-out (training the model on all but one year and testing it on the excluded year) cross-validation. The RF model effectively captures daily dust concentrations in unseen training years (Supplementary Fig. 3b) and stations (Supplementary Fig. 3c), yet overestimates concentrations below 0.1 μg m^−3^ and underestimates the highest concentrations above 10 μg m^−3^ (Supplementary Fig. 3b,c), leading to an overall positive bias (fractional bias for leave-one-year-out: 30.1%; fractional bias for leave-one-station-out: 38.2%). When averaged annually across unseen locations, the model shows much better agreement with the measurements (fractional bias: 14.2%), making it well suited for long-term trend analysis (Supplementary Fig. 4). DREAM dust concentrations contribute the most to the predictions (RF weight 0.32), whereas the dust optical depth and meteorological variables also have an important effect (Supplementary Fig. 5). By contrast, land use variables have a small impact (RF combined weight < 0.18), consistent with most of Al arising from transported dust and not local sources^40,41^, while at the same time indicating that, even for background dust, transported dust is its dominant component, even though it is a mixture of both local sources and desert dust from low-intensity events. The RF model accurately predicts multiyear dust concentrations and intrusions at the sites with the longest time series (Supplementary Fig. 6), without exhibiting a time-dependent bias (Supplementary Fig. 7). It outperforms DREAM by not only capturing high dust episodes but also moderate and low dust events (dust concentrations < 20 μg m^−3^) (Supplementary Fig. 3). As a study case, the RF model captures the plume path and magnitude of the most severe and heavily studied Saharan dust events that hit the Balkans in April 2019, showing close agreement in plume path and ground-level concentrations (30–80 μg m^−3^)^42^, supporting the suitability of the model for analysing dust frequency and intensity across Europe (Supplementary Fig. 8). By comparison, the physical DREAM model exhibits a strong negative bias at concentrations below 20 μg m^−3^ and a tenfold positive bias at concentrations above 50 μg m^−3^ (Supplementary Fig. 3a). Overall, the RF model outperforms DREAM in capturing spatiotemporal variations in dust loadings over Europe. The RF model shows high accuracy in regions with dense station clusters (for example, southern Spain), in which dust concentrations are highest. By contrast, its accuracy is expected to be lower in eastern Europe and Scandinavia owing to fewer measurements, although these areas are less affected by transported dust. Given the extensive dataset that powers the model, the lack of severe biases and the ability of the model to accurately capture intrusions both large and small, the RF model is uniquely suited for analysing dust in Europe.

**Fig. 2:**
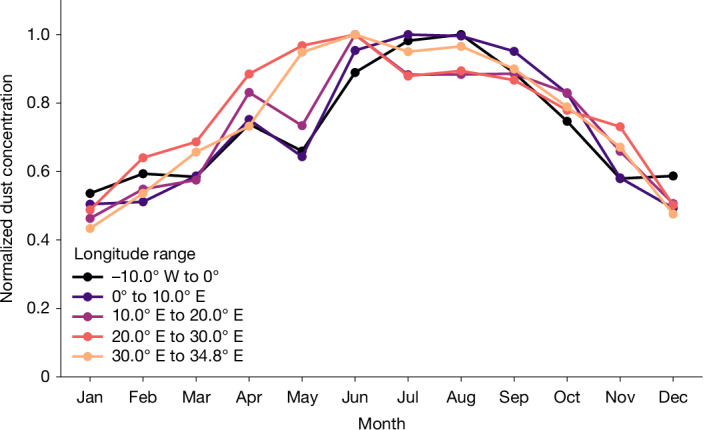
Dust seasonality in southern Europe. Monthly variation of normalized dust concentration across different longitudinal bands in southern Europe (land surface from 34° to 45° N) is presented. Normalized dust concentrations are calculated by dividing the mean monthly concentrations for the entire study decade (2012–2021) by the maximum mean monthly concentration within the respective longitudinal band. Source data

Using the RF model, we generated a decadal European dust phenomenology for assessing transported dust, enabling an investigation into the trends in dust intrusion severity and frequency over the study period (2012–2021). For all of the analysis that follows, southern Europe is defined as all of the land surface of Europe below and including the latitude of Milan (45° N) and northern Europe as all land surface above that. In southern Europe, dust concentration shows a pronounced seasonal variation, with the lowest concentrations in winter (Fig. 2). However, seasonal patterns differ between the eastern and western Mediterranean areas (Fig. 2 and Supplementary Figs. 9 and 10). In the east, dust concentrations peak between March and May, a period during which levels in the west (for example, Spain) remain relatively low, and persist until October or November (Fig. 2), consistent with previous studies^5,43^. Instead, the west experiences its dust peak in July–August, indicating a spatially dependent seasonality, with the east having a longer dust season than the west (Fig. 2 and Supplementary Figs. 9 and 10).

**Fig. 3:**
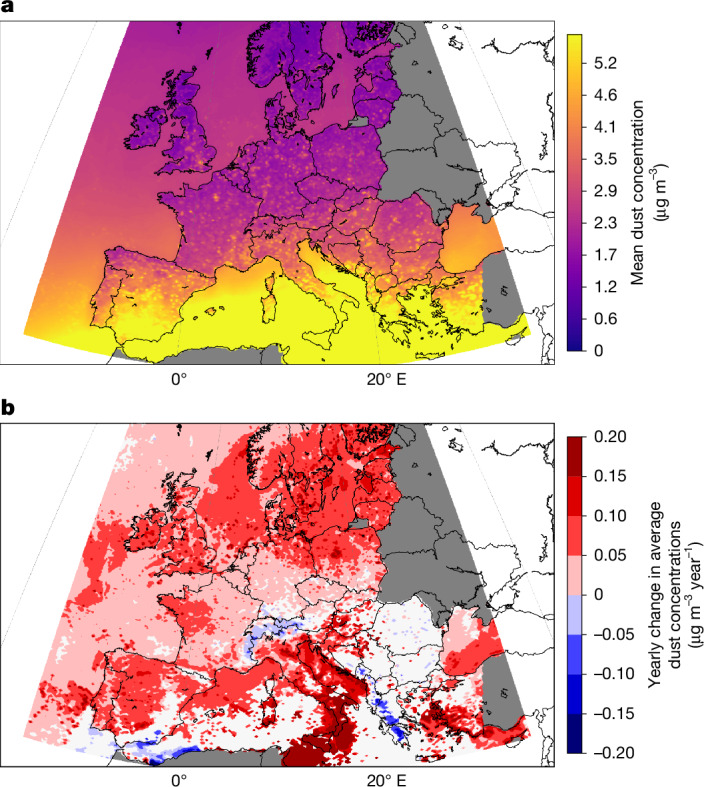
Dust concentrations and their changes in Europe during the period 2012–2021. **a**,**b**, Mean dust concentrations (**a**) and trend (**b**). Concentrations are reported in μg m^−3^ and their corresponding trends in μg m^−3^ year^−1^. A trend is defined as the mean of a 100 times-bootstrapped linear regression for the variable of interest and shown on the map if there is no sign change between the 25th and 75th percentiles of the bootstrap results. Areas in which a sign change occurs are denoted with white (for regional means considered zero), whereas areas in which no land use data exist are denoted in grey. Source data

Yearly dust concentrations are predictably higher in southern Europe than in the north by a factor of 2.5 (5.28 ± 2.65 μg m^−3^ versus 2.09 ± 1.05 μg m^−3^) (Fig. 3a). To assess changes in dust concentrations, we applied linear regression to the annual time series at each grid cell, using 100 bootstrap resamples. The reported change in dust concentration is the mean of the bootstrap slopes and cells in which zero lies within the interquartile range are masked in white (Fig. 3b, for regional means considered zero). For most of Europe, concentrations increase by 0.055 ± 0.022 μg m^−3^ year^−1^ between 2012 and 2021, with the largest increases over Italy and the Adriatic and Aegean seas at 0.074 ± 0.030 μg m^−3^ year^−1^ (Fig. 3b). Notably, in northern Europe, including Scandinavia, the magnitude of the increases is identical to that of the south (0.055 ± 0.022 μg m^−3^), suggesting that transported dust could become a growing concern for these regions in the future. A potential consideration for northern Europe is not only transported dust from the south but also Icelandic dust. Iceland is the most important dust source for the high-latitude areas of Europe and is also included in the model through DREAM^44^, and therefore can contribute to the observed changes in concentration in northern Europe as well as to North African dust.

**Fig. 4:**
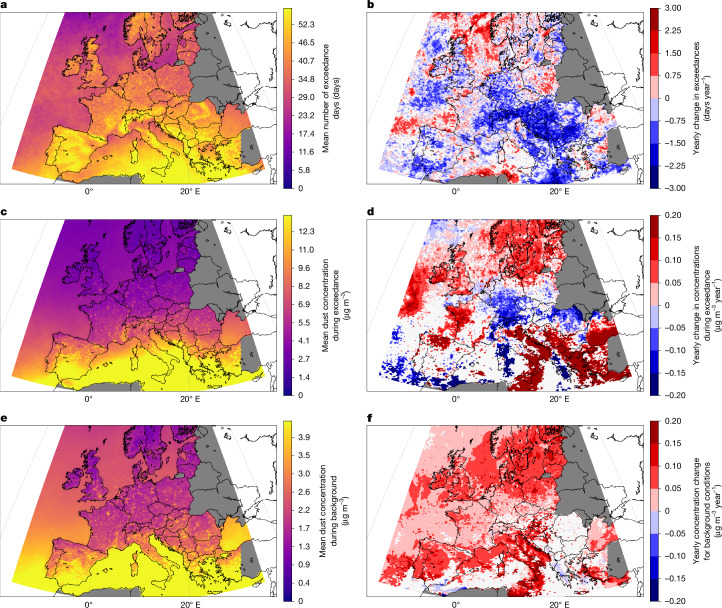
Dust episodes and their trends in Europe during the period 2012–2021. **a**–**f**, Mean number of days on which a dust exceedance occurs (**a**) and trend (**b**), mean dust exceedance concentrations (**c**) and trend (**d**) and mean dust concentrations during background (**e**) and trend (**f**). Concentrations are reported for PM_10_ dust in μg m^−3^ and their corresponding trends in μg m^−3^ year^−1^, whereas the number of exceedance days is reported as days, with their corresponding trend reported as days year^−1^. A trend is defined as the mean of a 100 times-bootstrapped linear regression for the variable of interest and shown on the map if there is no sign change between the 25th and 75th percentiles of the bootstrap results. Areas in which a sign change occurs are denoted with white (for regional means considered zero), whereas areas in which no land use data exist are denoted in grey. Source data

Daily dust levels exhibit pronounced variations, strongly affected by dust events. In accordance with present European Commission recommendations^24^, we define transport events as deviations from a background (which includes both local and transported sources) varying over time for each location. For this, we determine for each day and each grid cell a concentration threshold (*C*_th_) describing an upper concentration limit expected for background conditions (for details, see Methods section ‘Dust event detection’). This is a local and relative metric resulting in the number of detected transport events varying temporally and geographically. Southern Europe, including Spain, Italy and the eastern Mediterranean, experiences around 46 ± 7.82 dust intrusion days per year (Fig. 4a). However, compared with the average dust concentration (Fig. 3a), the north–south gradient is less pronounced (Fig. 4a). At higher altitudes, such as the Alps, Carpathians and Pyrenees, more dust intrusions are detected than in nearby lower-altitude areas, comparable with their number in the south. Conversely, dust concentrations during intrusions show a clear north–south gradient, similar to the average concentrations, with the highest levels observed in the south, with an average of 11.00 ± 5.51 μg m^−3^, gradually diminishing towards the north to an average value of 3.30 ± 1.65 μg m^−3^ (Fig. 4c).

## Changes in dust intrusions

The analyses of dust intrusion trends (Fig. 4b,d) reveal notable contrasts with average dust concentrations (Fig. 4a,c,e, analysis analogous to yearly means in Fig. 3). In northern Europe, including Scandinavia and the British Isles, background dust levels rise by 0.044 ± 0.018 μg m^−3^ year^−1^, nearly double the increase of 0.022 ± 0.005 μg m^−3^ year^−1^ during exceedances (Fig. 4f), suggesting that dust intrusions reaching these regions have not become more severe but rather the background dust levels have increased. It should be noted that, owing to the relative nature of the exceedance metric, the background also includes dust events of concentrations below the threshold and therefore it is expected that the observed increase is still attributed to transport. This interpretation is further supported by the fact that the increase during background conditions is observed over both land and sea—consistent with the spatial footprint expected for transported dust. Another potential driver is decreasing soil moisture owing to warming, albeit that the model-predicted Al seems insensitive to perturbations of soil parameters (Supplementary Fig. 11).

By contrast, southern Europe experiences a substantial rise in intrusion severity over the study period. In particular, dust concentrations during intrusion events increase by 1.40 ± 0.33 μg m^−3^ over the decade in the Adriatic–Ionian region, the Aegean and much of the Balkans. The trend is even more pronounced in southern Italy, in which intrusion-related concentrations rise by 0.270 ± 0.063 μg m^−3^ year^−1^. These increases are mainly driven by more intense intrusion events, as changes in background concentrations remain comparatively small—approximately 0.032 ± 0.013 μg m^−3^ year^−1^ and 0.063 ± 0.025 μg m^−3^ year^−1^, respectively (Fig. 4f).

Meanwhile, the number of exceedance days (Fig. 4b) declines across Europe from 2012 to 2021, despite a decreasing threshold concentration for exceedance detection, suggesting a reduction in intrusion frequency. Eastern Europe, the Alpine regions and the eastern Mediterranean, including Greece, show fewer exceedance days but increased intrusion severity (Fig. 4b,d). In regions with the sharpest rise in exceedance concentrations, particularly southeastern Europe, the number of exceedance days generally remained unchanged. Overall, although the frequency of dust intrusions has not increased, their severity has greatly increased, along with a notable increase in background concentrations. Similar trends have been observed in the past with satellite data for the period 2000–2007, with increased intrusion severity and slightly reduced frequency^45^.

## Climatic and short-term drivers of dust

**Fig. 5:**
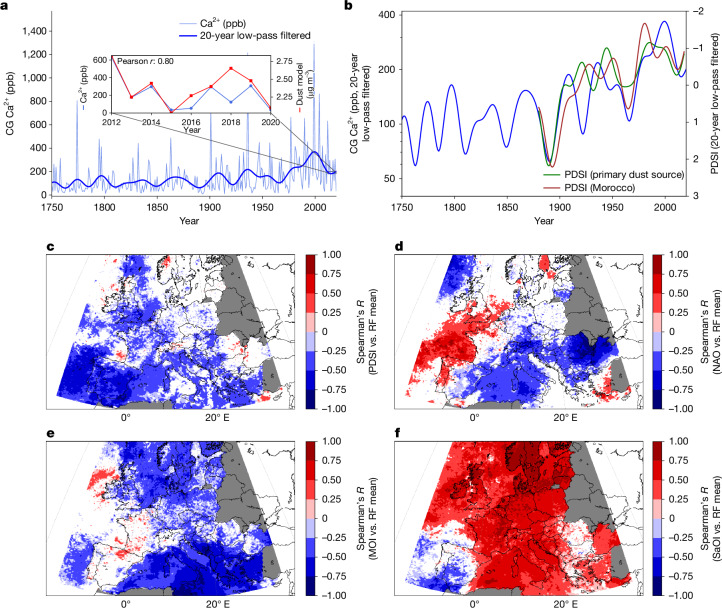
Long-term climatological trends and short-term variations in dust. **a**,**b**, Time series of Colle Gnifetti (CG) ice core Ca^2+^ concentrations (annual, thin blue line; 20-year low-pass filtered, bold blue line) from 1750 to 2020 and comparison with model results for the period 2012–2020 (inset) (**a**), 20-year low-pass filtered Ca^2+^ concentrations (blue) and the PDSI in the main source region of dust for the Swiss–Italian Alps in the northwestern Sahara (−7.14°–11.27° E, 32.15°–35.66° N, green) and Morocco (−10°–0° E, 30°–35° N, brown) (**b**), along with the spatiotemporal correlation between annual mean dust concentrations in the period 2012–2021 and PDSI in the extended Sahara area (14.2°–30.17° N, −9.15°–32.52° E) (**c**), NAO index (**d**), MOI (**e**) and SaOI (**f**). Results for the correlations are presented as the mean of a 100 times-bootstrapped linear regression for the variable of interest and RF results and shown on the map if there is no sign change between the 25th and 75th percentiles of the bootstrap results. Areas in which a sign change occurs are denoted with white (for regional means considered zero), whereas areas in which no land use data exist are denoted in grey. This analysis is designed to reveal statistically significant associations, not causal relationships; however, the robustness of these correlations provides valuable insights into the drivers of the short-term variability in dust over Europe. ppb, parts per billion. Source data

To assess whether the observed decadal trends reflect a sustained long-term increase in dust concentrations, we analysed Ca^2+^ concentration levels in Alpine ice cores from Colle Gnifetti (Monte Rosa, 4,450 m above sea level, 45° 55′ 46″ N; 07° 52′ 30″ E) for the period 1750–2020. The dataset combines three cores: one drilled in 2021 and two parallel cores recovered in 2003 (refs. ^46,47^). Because Al concentration data were only available until 1990, Ca^2+^ was used as a proxy for dust, an appropriate choice given the strong correlation between Ca^2+^ and Al during the overlapping period (*r* = 0.87, 1750–1990) and the absence of local sources at Colle Gnifetti. Although the ice core record reflects total dust deposition rather than atmospheric concentrations, it exhibits a consistent temporal pattern with the modelled dust concentrations over the nine overlapping years (2012–2020; Fig. 5a, inset), including a slight decline over the past decade—consistent with modelled trends across the Alps (Fig. 3b). Over the long term, Ca^2+^ concentrations preserved in the ice archive increased by approximately 110% between the pre-industrial period (1750–1850) and the past decade (2010–2020) (Fig. 5a). The trend derived from the three Colle Gnifetti cores is consistent with the multisite analysis in Kok et al.^10^, which reports a similar but less pronounced increase over the twentieth century. On interannual scales, Ca^2+^ concentrations in the ice core are correlated with the NAO (Spearman’s rank correlation coefficient (*R*_s_) = 0.48), consistent with the established influence of NAO-related circulation variability on dust transport^9,20,23^. Also, the long-term trend in Ca^2+^ exhibits a strong negative correlation with the self-calibrating Palmer Drought Severity Index (PDSI)^48,49^, which characterizes hydroclimatic variability over the primary dust source regions for the Swiss–Italian Alps, located in the northwestern Sahara (32.15°–35.66° N; −7.14°–11.27° E, following Coen et al.^50^). Drier conditions—corresponding to lower PDSI values—are associated with higher Ca^2+^ levels in the ice core (*R*_s_ = −0.70). When restricting the analysis to Moroccan dust sources, the correlation strengthens further (*R*_s_ = −0.78). These statistically robust associations indicate that long-term dust variability recorded in the Alpine archive is most strongly linked with persistent aridification trends in North Africa and with large-scale circulation variability, as represented by the NAO, which modulates precipitation and surface conditions over key dust source regions^21^ (Fig. 5a,b and Supplementary Fig. 12).

In Fig. 5d–f, we examine how circulation-oriented climate indices are statistically associated with dust inputs to Europe, alongside the dryness of the Sahara (Fig. 5c), considering a broad source region encompassing most of the Sahara (14.20°–30.17° N, −9.15°–32.52° E). The Saharan Oscillation Index (SaOI) represents the pressure difference between the Azores (37.79° N, −25.5° E) and Niamey (13.51° N, 2.10° E)^51^, whereas the Mediterranean Oscillation Index (MOI)^52^ corresponds to the normalized pressure difference between Algiers and Cairo. Both indices are closely related to the NAO^51,52^. During 2012–2021, dust concentrations were strongly correlated with the SaOI and anticorrelated with the MOI (Fig. 5e,f), except in western Europe, in which correlations were weaker and occasionally of opposite sign. This pattern may reflect the dual influence of the MOI: although low MOI values are associated with enhanced westward dust transport, they also coincide with higher precipitation over western Europe^52^, probably offsetting increases in surface dust concentrations through wet deposition. Increasing SaOI values were most strongly correlated with enhanced Saharan dust advection towards Europe, whereas declining MOI values were associated with reduced eastward transport and a relative strengthening of dust inflow to central and southern Europe. These combined circulation tendencies are most strongly linked to intensified southerly winds from North Africa, consistent with the spatial dust patterns shown in Fig. 4d,f.

Similarly, the NAO exhibits a robust statistical relationship with dust transport. Positive NAO phases during 2012–2021 were associated with stronger cyclonic activity and westward transport, corresponding to higher dust levels over the Atlantic, France and Spain^41^ (Fig. 5d), whereas stronger westerlies under these conditions coincided with reduced dust transport into central and eastern Europe. Over the Aegean Sea, increased dust concentrations during positive NAO phases are most probably linked to intensified easterly winds in the far eastern Mediterranean^43^.

Overall, the observed dust variability across Europe is most strongly correlated with concurrent trends in North African desertification and regional circulation patterns, emphasizing the dual influence of source region aridity and large-scale atmospheric dynamics on dust transport towards Europe.

## Implications

By 2021, our model shows that, in southern Europe, transported dust alone already accounted for 31% of World Health Organization (WHO)^53^ annual mean PM_10_ guideline values (15 µg m^−3^) and 25.8% (calculated on the basis of observed PM_2.5_:PM_10_ elemental ratios) of PM_2.5_ guideline values (5 µg m^−3^), substantially hindering these regions to comply with regulations. Residents in the region experience 46 ± 7.82 dust episodes annually, with dust levels averaging 9.68 ± 4.85 μg m^−3^ during these events. We estimate that short-term exposure to such dust concentrations would be associated with a 0.67% ± 0.02% contribution to daily mortality and a 0.73% ± 0.04% contribution to daily respiratory hospitalizations during dust events among patients older than 15 years, as in Stafoggia et al.^4^ (Methods; using global relative risk coefficients leads to similar results presented in Supplementary Table 6). The impact on younger patients is even more pronounced, with a contribution of 2.47% ± 0.07% in daily respiratory hospitalizations during dust events, highlighting more severe impacts of dust on vulnerable populations^4^. Although the increase in dust concentrations observed during the 10-year study period was not sufficient to cause substantial changes in health outcomes, continued increases in dust levels could exacerbate these health risks. We demonstrate that dust deposited in the Alps has increased by 110% since pre-industrial times owing to desertification, whereas shifts in synoptic circulation patterns have intensified intrusions, raising the annual dust PM_10_ concentrations by 2.2 ± 0.51 μg m^−3^ in the south of Italy over the past decade. With climate change accelerating desertification and altering atmospheric circulation, dust pollution will increasingly threaten public health and hinder efforts to meet WHO and European Union air quality targets—a direct feedback of climate change on air quality.

## Methods

### Dataset and database construction

We compiled an extensive database of daily average measurements of PM_10_ elements related to dust (Al, Ti, Si, Ca and Fe) from all over Europe, to estimate dust concentrations. The bulk of the data was compiled ad hoc for this study, whereas some were extracted from the EBAS database^54^. The most frequently measured metal that is prevalent in mineral dust was aluminium; measurement data for other dust metals found purely in dust such as silicon and titanium were much scarcer. By contrast, local sources such as road dust as well as non-exhaust traffic and construction emissions affect calcium and iron, making them unsuitable for estimating transported dust in this study^40^. Metal concentrations were measured with both offline filter-based techniques (inductively coupled plasma (ICP), particle-induced X-ray emission (PIXE) and offline X-ray fluorescence (XRF))^55^ and online techniques (online XRF X-ray induced acoustic computed tomography (XACT))^55,56^. Al measurements from these techniques are directly comparable for the purposes of trend analysis, as numerous inter-laboratory comparison studies have shown that these techniques yield highly consistent results for particulate elemental concentrations^57–61^. Also, grouping model RF cross-validation results by training technique (XRF-based, ICP-based or PIXE; Supplementary Fig. 13) does not indicate any inherent biases between the techniques. Great care was taken in curating the database by flagging data below detection limits and working with data providers to ensure high data quality. After data were aggregated from all providers, various methods were used to ensure data quality. Low concentration values at the detection limit were discarded. To that end, concentration values below a detection cut-off of Al = 0.003 µg m^−3^ were removed. Also, values that were repeated more than three times in sequence or repeated more than five times (at a third decimal precision) were interpreted as data that were replaced by the detection limit. Further data with a time resolution >1 day were discarded, whereas hourly data were aggregated to daily averages.

### Uncertainty of elemental ratios and dust estimate

We quantified the impact of uncertainty in the elemental ratios—defined here as one standard deviation from the bootstrap results shown in Fig. 1b (Si:Al 2.610 ± 0.033, Ti:Al 0.068 ± 0.003, Ca:Al 1.580 ± 0.099 and Fe:Al 0.890 ± 0.052)—on the dust estimates using uncertainty propagation based on the formula: 2.2Al + 2.49Si + 1.63Ca + 1.94Ti + 2.42Fe. This results in a total dust estimate of (13.5 ± 0.377) × Al. Notably, the largest contributions to the overall uncertainty originate from Fe and Ca, which are also influenced by substantial local sources. Zero-intercept fits were used for element–element plots (for example, Ca concentration versus Al concentration) to estimate dust phase mass-based elemental ratios under the assumption that—after blank correction and removal of non-dust contributions—both analytes originate from the same dust endmember. In that case, when the dust contribution vanishes (Al → 0), the co-emitted element should also vanish (Ca → 0), implying a physically meaningful intercept of zero. Also, we tested a linear model with an intercept using bootstrap analysis and found that zero lies within one standard deviation of the mean intercept (intercept ± *σ*_intercept_), supporting the use of a zero-intercept model. Given this result, along with the physical consistency and practical advantages of a zero-intercept regression—such as enabling the use of average ratios to estimate other components—we opted for the zero-intercept model. A summary of the relevant statistics is provided in Supplementary Table 2.

Elemental ratios on days with low amounts of transported dust can be substantially affected by local sources. With a rather high aluminium concentration threshold of >1 μg m^−^^3^ for determining the transported dust elemental ratios, we aimed at minimizing the impact of local sources on the elemental ratios. Also, we performed a sensitivity analysis varying the threshold, showing that the elemental ratios vary with cut-offs above 0.5 μg m^−3^ for Ca:Al and 0.75 μg m^−3^ for Fe:Al within one standard deviation of the value obtained with a cut-off of 1 μg m^−^^3^. Further, we investigated model-driven criteria for dust episodes instead of using a static aluminium concentration cut-off using the DREAM and optical depth values and only including data points that were higher than the 90th percentile in those (each alone or both at the same time), as a proxy for dust events. This approach resulted in similar values (Fig. 1b and Supplementary Fig. 14).

The uncertainty *σ*_mean_ in modelled yearly mean dust concentrations dust_mean_ (including exceedance and background concentrations) is estimated by accounting for two main components: (1) the uncertainty in the derived dust:Al ratio (RE_ratio_ = 0.377/13.5 = 2.7%) and (2) the relative root mean square error of the modelled annual mean dust concentrations (RRMSE_annual mean_ = 0.45; Supplementary Fig. 3a), as determined through a leave-one-out performance analysis against chemically derived dust concentrations from in situ measurements (equation(1)).

1 $${\sigma }_{{\rm{mean}}}=\sqrt{{({{\rm{RRMSE}}}_{\text{annual mean}}\times {{\rm{dust}}}_{{\rm{mean}}})}^{2}+{({{\rm{RE}}}_{{\rm{ratio}}}\times {{\rm{dust}}}_{{\rm{mean}}})}^{2}}=\sqrt{{({{\rm{RRMSE}}}_{\text{annual mean}})}^{2}+{{\rm{RE}}}_{{\rm{ratio}}}^{2}}\times {{\rm{dust}}}_{{\rm{mean}}}$$

The robustness of dust concentration trends over the 10-year study period was evaluated by bootstrapping the linear regression of annual dust concentration time series at each grid cell (100 bootstrap resamples). The reported trend *t*_dust_ represents the mean of the bootstrap-derived slopes, in which grid cells where the interquartile range includes zero are masked in white (zero trends are assumed for regional means in such cases). The overall uncertainty *σ*_trend,conc_ in the trend (equation (2)) includes both uncertainty in the dust:Al ratio and the bootstrap-based variability in fitting the trend (RE_boot_, domain mean relative uncertainty excluding grid cells with non-significant trends), whereas the precision in the yearly dust prediction is already accounted for by the latter (see equation (1)):

2 $${\sigma }_{{\rm{trend,conc}}}=\sqrt{{({{\rm{R}}{\rm{E}}}_{{\rm{r}}{\rm{a}}{\rm{t}}{\rm{i}}{\rm{o}}}\times {t}_{{\rm{d}}{\rm{u}}{\rm{s}}{\rm{t}}})}^{2}+{({{\rm{R}}{\rm{E}}}_{{\rm{b}}{\rm{o}}{\rm{o}}{\rm{t}}}\times {t}_{{\rm{d}}{\rm{u}}{\rm{s}}{\rm{t}}})}^{2}}=\sqrt{{({{\rm{R}}{\rm{E}}}_{{\rm{r}}{\rm{a}}{\rm{t}}{\rm{i}}{\rm{o}}})}^{2}+{({{\rm{R}}{\rm{E}}}_{{\rm{b}}{\rm{o}}{\rm{o}}{\rm{t}}})}^{2}}\times {t}_{{\rm{d}}{\rm{u}}{\rm{s}}{\rm{t}}},$$

with $\sqrt{{({{\rm{RE}}}_{{\rm{ratio}}})}^{2}+{({{\rm{RE}}}_{{\rm{boot}}})}^{2}}$ equal to 0.5, 0.23 and 0.4 for the trend in mean, exceedance and non-exceedance concentrations, respectively.

Analogously, we estimate the uncertainty *σ*_ed_ of the number of exceedance days and related trends. We use counting statistics to describe the uncertainty of the number of days (RE_d_ = 0.17 being the domain mean relative uncertainty based on $\sqrt{{\rm{d}}{\rm{a}}{\rm{y}}{\rm{s}}}/{\rm{d}}{\rm{a}}{\rm{y}}{\rm{s}}$) (equation (3)).

3 $${\sigma }_{{\rm{ed}}}={{\rm{RE}}}_{{\rm{d}}}\ast {\rm{days}}$$

The domain mean relative uncertainty *σ*_trend,day_ (excluding grid cells with non-significant trends) in the trend of exceedance days *t*_days_ is estimated by means of the bootstrap-based relative variability (RE_db_ = 0.27) in fitting the trend (equation (4)), which also accounts for the precision in the yearly prediction in exceedance days (see equation (3)).

4 $${\sigma }_{\mathrm{trend,day}}={{\rm{RE}}}_{{\rm{db}}}\times {t}_{{\rm{days}}}$$

### Machine learning model

We created a dust predicting model using RF^34,36,62–64^, which is an ensemble learning method that constructs several decision trees during training and outputs the mean prediction of all individual trees. Each tree in the RF is trained on a bootstrapped subset of the data and node splits occur on the basis of a randomly selected number of features, leading to a reduction in overfitting and variance. RF was selected because it provided a balance between bias and variance and owing to its robustness to noisy data and feature correlation, especially given that aluminium concentrations can span many orders of magnitude, from a few nanograms to tens of micrograms. We use the sklearn Python package RandomForestRegressor, which is based on the Breiman implementation of RFs^64,65^. The model hyperparameters including the number of trees (n_estimators), maximum depth (max_depth) and minimum samples per split (min_samples_split) were tuned and optimized to ensure the best possible validation (parameters used in this work are n_estimators=150, max_depth=None, criterion=‘friedman_mse’, bootstrap=True, random_state=66). RF also permits the derivation of the importance of each individual feature, allowing for the physical interpretation of the results.

### Model inputs

The most influential input that drives the machine learning model are the daily transported dust fields provided by DREAM. DREAM is an Eulerian model that resolves the equations for aeolian dust emission and dispersion. It includes all of the relevant processes for dust emission and transport, from convection to deposition. A detailed description of the model can be found in ref. ^33^. We used the surface dust concentration model variable (SCONC_dust; μg m^−3^) at a model resolution of 0.33° by 0.33°. As well as DREAM, other model inputs included CERRA sub-daily regional reanalysis data for Europe, specifically surface level temperature, precipitation, wind speed and wind direction^66^, along with satellite column integrated dust optical depth (550 nm) from CAMS global reanalysis (EAC4)^67^ for the years 2012–2021 (Supplementary Table 3). Finally, coordination of information on the environment (CORINE) annual land-use data at 200 m resolution for the whole of Europe were also used as RF model inputs for the training dataset (that is, stations), including the fractions of 14 land use types^68^ (for example, urban fraction, natural green, agriculture and barren land), population density^69^, altitude^70^ and road length and category^71^. Vegetation is already accounted for in the model through the natural green land use variable; however, seasonal vegetation changes were not explicitly included. A comprehensive list of variables can be found in Supplementary Table 4. To improve spatial coverage, all data were harmonized by cubic interpolation, to a 10 × 10 km-resolution grid extending from the northern part of Tunisia to the edge of Norway, similar to the grid used in ref. ^38^. For grid cells with measurements over the sea, all land-use categories are set to 0 and the water fraction is set to 1.

Although RF is inherently resilient to potential collinearities^72^, to evaluate potential redundancy between DREAM-modelled dust and meteorological predictors, we examined the contribution of each variable to the RF model performance using both Shapley Additive Explanations (SHAP) values and variable importance metrics. DREAM dust emerged as the single most influential predictor, accounting for roughly 30% of the explained variance in model outputs, whereas individual meteorological variables (for example, wind speed) contributed substantially less (<5% each). This dominance of DREAM dust in both metrics, combined with the distinct physical meaning of this variable—representing long-range dust emission and transport processes rather than local meteorology—demonstrates that it provides independent explanatory power rather than redundant information.

### Model performance

The model was optimized by hyperparameter testing, with the optimal parameters being n_estimators=150, max_depth=None, criterion=‘friedman_mse’, bootstrap=True and random_state=66. The criterion for picking the optimal model was the average Pearson *r* value for all of the sites from the leave-one-station-out validation, that is, the model configuration that had the highest *r* across all sites. It was chosen over other absolute metrics such as root mean square error owing to the measurements spanning many orders of magnitude (Supplementary Figs. 2 and 3), which would lead to sites in the south with larger average concentrations, overpowering sites in the north. It is noteworthy that all configurations attempted yielded very similar results, indicating that the method was insensitive to the hyperparameters.

The RF model was trained using observations from 2013–2021, whereas reconstructed dust concentrations cover the analysis period 2012–2021. The year 2012 therefore represents an out-of-sample prediction. Notably, ‘year’ was not included as a predictor variable, preventing the model from learning or imposing artificial temporal trends. Consequently, trends in reconstructed dust concentrations arise solely from variations in the meteorological and environmental predictors rather than from the model structure itself.

A leave-one-year-out validation, that is, removing an entire year from the training dataset and predicting it, was also conducted, yielding similar results to the leave-one-station-out validation, indicating that the model carries no inherent spatiotemporal biases (Supplementary Tables 5 and 7 and Supplementary Figs. 7 and 15). To further validate the model, and test its generalizability, we conducted a extra temporal validation for the year 2012, which was not included in the training of the model^25^. Also, these sites, with the exception of Athens and Finokalia, were also not included in the training dataset, meaning that the spatial and temporal predictive abilities of the model were tested at the same time. The year was not used as a predictor variable, preventing the model from learning or imposing artificial temporal trends. Model performance for the unused year 2012 is very similar to that of the other years used in training (Supplementary Table 7 and Supplementary Fig. 15), reinforcing the robustness of the model.

Model training is completed within less than 5 min, whereas producing dust fields for 10 years and for the entire domain takes 53 min on 60 processors (two cores per processor).

### Dust event detection

For the detection of dust transport events, we determine for each day and each grid cell a concentration threshold (*C*_th_) describing an upper concentration limit expected for background conditions. This methodology is based on the working paper by the European Commission for event identification^24^. When the observed dust concentration exceeds this threshold concentration, the day in question is classified as a transport event for this grid cell. This daily threshold concentration for each grid cell of the domain is based on the rolling median of the 30 preceding and 30 following days (*C*_Med,30 prec._, *C*_Med,30 foll._), along with their median absolute deviation (MAD_30 prec._, MAD_30 foll._). Specifically for an exceedance to occur, the dust concentration needs to be higher than a threshold value, defined as (equation (5)):

5 $${C}_{{\rm{t}}{\rm{h}}}=\frac{({C}_{{\rm{M}}{\rm{e}}{\rm{d}},30{\rm{p}}{\rm{r}}{\rm{e}}{\rm{c}}.}+{C}_{{\rm{M}}{\rm{e}}{\rm{d}},30{\rm{f}}{\rm{o}}{\rm{l}}{\rm{l}}.})}{2}+3\frac{({{\rm{M}}{\rm{A}}{\rm{D}}}_{30{\rm{p}}{\rm{r}}{\rm{e}}{\rm{c}}.}+{{\rm{M}}{\rm{A}}{\rm{D}}}_{30{\rm{f}}{\rm{o}}{\rm{l}}{\rm{l}}.})}{2}.$$

### Health impacts from short-term exposure to dust

To estimate the increase in mortality owing to short-term exposure to dust, we first calculated the fractional increase in mortality resulting from dust, based on the increase in risk for all-cause mortality (denoted IR) owing to acute dust exposure provided in ref. ^4^, per 10 μg m^−3^ increase in dust exposure. The corresponding IRs were 0.65% ± 0.02% for all-cause mortality with a 0–1 lag day, 0.70% ± 0.02% for respiratory hospitalizations of the older than 15 years age group with 0–5 lag days and 2.47% ± 0.07% for respiratory hospitalizations for the 0–14 years age group with 0–5 lag days. This increase is the population weighted to account for the differences in population density. Assuming an exponential dose–response function, the percent increase in mortality or hospitalizations for a given grid area then becomes (equation (6)):

6 $${\rm{ \% }}\,\text{increase in mortality}=100{\rm{ \% }}\,\times \mathop{\sum }\limits_{i=1}^{{\rm{n}}{\rm{o}}.\,{\rm{o}}{\rm{f}}\,{\rm{p}}{\rm{o}}{\rm{i}}{\rm{n}}{\rm{t}}{\rm{s}}}\frac{{\rm{p}}{\rm{o}}{\rm{p}}{\rm{u}}{\rm{l}}{\rm{a}}{\rm{t}}{\rm{i}}{\rm{o}}{\rm{n}}\,{\rm{o}}{\rm{f}}\,{\rm{c}}{\rm{e}}{\rm{l}}{\rm{l}}\,{i}}{{\rm{t}}{\rm{o}}{\rm{t}}{\rm{a}}{\rm{l}}\,{\rm{p}}{\rm{o}}{\rm{p}}{\rm{u}}{\rm{l}}{\rm{a}}{\rm{t}}{\rm{i}}{\rm{o}}{\rm{n}}}\times \left(1-\frac{1}{{e}^{{\rm{I}}{\rm{R}}\times {\rm{e}}{\rm{x}}{\rm{c}}{\rm{e}}{\rm{e}}{\rm{d}}{\rm{a}}{\rm{n}}{\rm{c}}{\rm{e}}{\rm{c}}{\rm{o}}{\rm{n}}{\rm{c}}{\rm{e}}{\rm{n}}{\rm{t}}{\rm{r}}{\rm{a}}{\rm{t}}{\rm{i}}{\rm{o}}{{\rm{n}}}_{{i}}}}\right),$$

in which no. of points is equal to the total number of grid cells in the area, population of cell *i* is the total population within the grid cell, IR is the increase in risk for all-cause mortality and exceedance concentration_*i*_ is the average exceedance concentration over the decade for that grid cell. For this calculation, the south is defined as all of the land surface of Europe below, and including, Milan (45° N), for which we calculate an increase of 0.67% ± 0.02% in all-cause mortality for exceedance days, a 0.73% ± 0.04% increase in daily respiratory hospitalizations among the older than 15 years age group and a 2.55% ± 0.07 rise in daily respiratory hospitalizations among the 0–14 years age group. The mortality calculations were repeated using the IRs from ref. ^73^ and shown in Supplementary Table 6.

## Online content

Any methods, additional references, Nature Portfolio reporting summaries, source data, extended data, supplementary information, acknowledgements, peer review information; details of author contributions and competing interests; and statements of data and code availability are available at 10.1038/s41586-026-10743-w.

## Supplementary information

Supplementary Information Supplementary Tables 1–7 and Supplementary Figs. 1–15

Peer Review file

## Source data

Source Data Fig. 1

Source Data Fig. 2

Source Data Fig. 3

Source Data Fig. 4

Source Data Fig. 5

## Data Availability

The full datasets shown in the figures and tables are publicly available at 10.5281/zenodo.19236528 (ref. ^74^). Source data are provided with this paper.

## Code availability

The random forest model was built in Python v3.9.10 using scikit-learn (https://scikit-learn.org/stable/) and is publicly available at 10.5281/zenodo.19236528 (ref. ^74^).
