# Supplementary material for: Rising dust pollution across Europe in a changing climate
Source: Nature. 2026 Jul 15;655(8123):647–54. doi: 10.1038/s41586-026-10743-w (PMC13372676; doi:10.1038/s41586-026-10743-w)
Supplement: Supplementary file 1 — Supplementary Tables 1–7 and Supplementary Figs. 1–15 [file 41586_2026_10743_MOESM1_ESM.pdf]

nature portfolio

https://doi.org/10.1038/s41586-026-10743-w

Supplementary information

Rising dust pollution across Europe in a changing climate

In the format provided by the authors and unedited

Supporting information

Rising dust pollution across Europe in a changing climate

Petros N. Vasilakos[1]; Abhishek Upadhyay[1]; Manousos I. Manousakas[1,2]; Andrés Alastuey[3]; James D. Allan[4,5]; Célia A. Alves[6]; Benjamin Bergmans[7]; Benjamin T. Brem[1]; Sonia Castillo[8]; Theodoros Christoudias[9]; Cristina Colombi[10]; Sébastien Conil[11]; Katja Dzepina[1,12]; Anja Eichler[1,13]; Konstantinos Eleftheriadis[2]; Olivier Favez[14]; Michael Flynn[4]; Kristina Glojek[3]; Stuart K. Grange[15,16]; David Green[17]; Christoph Hueglin[15]; Jean-Luc Jaffrezo[18]; Theo Manuel Jenk[1,13]; Jianhui Jiang[19]; Ekaterina Krymova[20]; Franco Lucarelli[21]; Petra Makorič[22]; Dario Massabò[23]; Nikolaos Mihalopoulos[9,24,25]; Griša Močnik[12]; Robin L. Modini[1]; Claudia Mohr[1]; Attilio Naccarato[26]; Petra Pokorná[27]; Paolo Prati[23]; Nicole Probst-Hensch[28,29]; André S. H. Prévôt[1]; Xavier Querol[3]; Cristina Reche[3]; Jesús D. de la Rosa[30]; Mark M. Scerri[31]; Jean Sciare[9]; Michael Sigl[1,13]; Anja Tremper[17]; Rita Traversi[32]; Daniel Trejo Banos[20]; Maria Tsagkaraki[24]; Gaëlle Uzu[18]; Roberta Vecchi[33]; Marta Via[12]; Kees de Hoogh[28,29]; Imad El-Haddad[1]; Kaspar R. Daellenbach[1]

[1] PSI Center for Energy and Environmental Sciences, Paul Scherrer Institute, Villigen, Switzerland
[2] Environmental Radioactivity & Aerosol Tech. for Atmospheric & Climate Impact Lab, INRaSTES, National Centre for Scientific Research "Demokritos", Athens, Greece
[3] Institute of Environmental Assessment and Water Research (IDAEA-CSIC), Barcelona, Spain
[4] Department of Earth and Environmental Science, The University of Manchester, Manchester M13 9PL, UK
[5] National Centre for Atmospheric Science, The University of Manchester, Manchester M13 9PL, UK
[6] Department of Environment and Planning, CESAM - Centre for Environmental and Marine Studies, University of Aveiro, 3810-193, Aveiro, Portugal
[7] Institut Scientifique de Service Public - ISSeP, Belgium
[8] Andalusian Institute of Earth System Research, IISTA-CEAMA, University of Granada, 18006 Granada, Spain
[9] Climate and Atmosphere Research Centre (CARE-C), The Cyprus Institute, Nicosia, Cyprus
[10] Agenzia Regionale per la Protezione dell'Ambiente Lombardia (ARPA Lombardia), 20124 Milan, Italy
[11] ANDRA DISTEC/EES Observatoire Pérenne de l'Environnement, F-55290, Bure, France
[12] Centre for Atmospheric Research (CRA), University of Nova Gorica, Ajdovščina, Slovenia
[13] Oeschger Centre for Climate Change Research, University of Bern, 3012 Bern, Switzerland
[14] Institut national de l'environnement industriel et des risques (Ineris), Parc Technol. ALATA, 60550 Verneuil en Halatte, France
[15] Laboratory for Air Pollution and Environmental Technology, Swiss Federal Laboratories for Materials Science and Technology (Empa), Duebendorf, Switzerland
[16] School of Earth and Atmospheric Sciences, Queensland University of Technology (QUT), Brisbane, Queensland, 4000, Australia
[17] Environmental Research Group, MRC Centre for Environment and Health, School of Public Health,

Imperial College London, London, UK
[18] University Grenoble Alpes, CNRS, IRD, INP-G, INRAE IGE (UMR 5001), 38000 Grenoble, France
[19] Institute for Urban and Regional Sustainability School of Ecological and Environmental Sciences, East China Normal University, 200241 Shanghai, China
[20] Swiss Data Science Center, EPFL and ETH Zürich, Zürich, Switzerland
[21] Department of Physics and Astronomy, University of Florence and I.N.F.N., Sesto Fiorentino, 50019 Florence, Italy
[22] Laboratory for environmental and life sciences, University of Nova Gorica, Nova Gorica 5000, Slovenia
[23] Dipartimento di Fisica, Università di Genova, via Dodecaneso 33, 16146 Genova, Italy
[24] Environmental Chemical Processes Laboratory, Department of Chemistry, University of Crete, Heraklion, 71003, Greece
[25] Institute for Environmental Research and Sustainable Development, National Observatory of Athens, Lofos Koufou, P. Penteli, Athens, 15236, Greece
[26] Department of Chemistry and Chemical Technologies, Block 12D, I-87036, Arcavacata di Rende (CS), University of Calabria, Italy
[27] Institute of Chemical Process Fundamentals of the Czech Academy of Sciences, Prague, 16500, Czech Republic
[28] Swiss Tropical and Public Health Institute, Kreuzstrasse 2, 4123, Allschwil, Switzerland
[29] University of Basel, Basel, Switzerland
[30] CIQSO-Center for Research in Sustainable Chemistry, Associate Unit CSIC-University of Huelva "Atmospheric Pollution", Campus El Carmen s/n, 21071, Huelva, Spain
[31] Institute of Earth Systems, University of Malta, Msida MSD2080, Malta
[32] Department of Chemistry "Ugo Schiff", University of Florence, 50019 Sesto F.no (Florence), Italy
[33] Department of Physics, Università degli Studi di Milano, Milan 20133, Italy

***Correspondence to*:** Petros N. Vasilakos (email: petros.vasilakos@psi.ch), Imad El-Haddad (email: imad.el-haddad@psi.ch) & Kaspar R. Daellenbach (email: kaspar.daellenbach@psi.ch)

Table S1 | Sites with daily measurements included in the study and measurement technique deployed to measure Aluminium.

Country	Site Name	Latitude	Longitude	Al mean (μg m^{-3})	Measurement technique
**BELGIUM**	**Vielsalm**	**50.304**	**6.001**	**0.047**	**ICP**
**CYPRUS**	**Agia Marina Xyliatou/ Cyprus Atmospheric Observatory**	**35.038**	**33.058**	**0.531**	**ICPS**
**CZECH REPUBLIC**	**National Atmospheric Observatory Košetice – NAOK**	**49.959**	**17.691**	**0.247**	**XACT**
**ENGLAND**	**Harwell**	**51.573**	**-1.317**	**0.024**	**PIXE**
**ENGLAND**	**London Marylebone**	**51.52**	**-0.151**	**0.000**	**XACT**
**ENGLAND**	**Manchester-Fallowfield**	**53.466**	**-2.231**	**0.128**	**XACT**
**FRANCE**	**Observatoire Perenne de l'Environnement – ANDRA**	**48.562**	**5.506**	**0.025**	**PIXE/ICP**
**FRANCE**	**Puy de Dôme**	**45.772**	**2.965**	**0.054**	**PIXE**
**FRANCE**	**SIRTA Atmospheric Research Observatory**	**48.709**	**2.159**	**0.042**	**PIXE**
**FRANCE**	**Aix-en-provence**	**43.53**	**5.441**	**0.044**	**ICP**
**FRANCE**	**Chamonix**	**45.923**	**6.87**	**0.054**	**ICP**
**FRANCE**	**Grande Synthe**	**51.025**	**2.303**	**0.232**	**ICP**
**FRANCE**	**Grenoble (GRE-fr)**	**45.162**	**5.736**	**0.143**	**ICP**
**FRANCE**	**Lens**	**50.437**	**2.827**	**0.156**	**ICP**
**FRANCE**	**Marseille (MRS-5av)**	**43.306**	**5.396**	**0.084**	**ICP**
**FRANCE**	**Nice**	**43.702**	**7.286**	**0.100**	**ICP**
**FRANCE**	**Nogent sur Oise**	**49.276**	**2.482**	**0.118**	**ICP**
**FRANCE**	**Port-de-Bouc dB (PdB)**	**43.402**	**4.982**	**0.202**	**ICP**
**FRANCE**	**Poitiers**	**46.584**	**0.346**	**0.210**	**ICP**
**FRANCE**	**Revin**	**49.908**	**4.63**	**0.121**	**ICP**
**FRANCE**	**Roubaix**	**50.707**	**3.181**	**0.174**	**ICP**
**FRANCE**	**Strasbourg (STG-cle)**	**48.59**	**7.745**	**0.155**	**ICP**
**FRANCE**	**Talence**	**44.8**	**-0.588**	**0.023**	**ICP**
**GERMANY**	**Melpitz**	**51.526**	**12.928**	**0.073**	**PIXE**
**GREECE**	**Finokalia**	**35.338**	**25.669**	**1.126**	**ICP**
**GREECE**	**Athens**	**37.995**	**23.816**	**0.392**	**PIXE**
**HUNGARY**	**K-puszta**	**46.967**	**19.583**	**0.170**	**ICP**
**IRELAND**	**Mace Head**	**53.326**	**-9.899**	**0.121**	**PIXE**

**ITALY**	**Turin**	**45.074**	**7.676**	**0.105**	**ICP**
**ITALY**	**Ispra**	**45.8**	**8.633**	**0.083**	**PIXE**
**ITALY**	**Calenzano**	**43.852**	**11.176**	**0.347**	**PIXE**
**ITALY**	**Capannori**	**43.84**	**10.573**	**0.296**	**PIXE**
**ITALY**	**Capannori (Lucca)**	**43.84**	**10.57**	**0.182**	**PIXE**
**ITALY**	**Figline Vno (Florence)**	**43.62**	**11.47**	**0.170**	**PIXE**
**ITALY**	**Florence Bassi**	**43.786**	**11.287**	**0.181**	**PIXE**
**ITALY**	**Lecce (Costal southern Italy) 2016 - Site A and Site B**	**40.3**	**18.1**	**0.204**	**PIXE**
**ITALY**	**Montale**	**43.915**	**11.006**	**0.214**	**PIXE**
**ITALY**	**Lampedusa**	**35.52**	**12.63**	**0.333**	**PIXE**
**ITALY**	**Milan AIRUSE**	**45.479**	**9.235**	**0.246**	**XRF**
**ITALY**	**Milan Pascal**	**45.479**	**9.235**	**0.296**	**XRF**
**ITALY**	**Milan Senato**	**45.47**	**9.197**	**0.254**	**XRF**
**ITALY**	**Mantova-Schivenoglia**	**45.017**	**11.076**	**0.248**	**XRF**
**MALTA**	**Malta**	**35.896**	**14.49**	**0.589**	**XRF**
**POLAND**	**Krakow**	**50.065**	**19.945**	**0.261**	**ICP**
**PORTUGAL**	**Coimbra traffic**	**40.207**	**-8.437**	**0.220**	**PIXE**
**PORTUGAL**	**Coimbra Urban background**	**40.207**	**-8.425**	**0.173**	**PIXE**
**PORTUGAL**	**Porto**	**41.2**	**-8.55**	**0.437**	**PIXE**
**SCOTLAND**	**Auchencorth Moss**	**55.792**	**-3.243**	**0.017**	**ICP**
**SEA**	**Acicastello**	**37.538533**	**15.182997**	**0.810**	**ICP**
**SEA**	**C. Flegrei 1**	**40.779876**	**14.169802**	**0.225**	**ICP**
**SEA**	**C. Flegrei 2**	**40.770611**	**14.08416**	**0.158**	**ICP**
**SEA**	**Ischia**	**40.690414**	**13.898805**	**0.675**	**ICP**
**SEA**	**Marsili**	**39.249787**	**14.394479**	**0.622**	**ICP**
**SEA**	**Panarea**	**38.635426**	**15.103832**	**0.751**	**ICP**
**SEA**	**Stromboli 1**	**38.850744**	**15.230235**	**0.267**	**ICP**
**SEA**	**Stromboli 2**	**38.748674**	**15.22142**	**0.575**	**ICP**
**SEA**	**Stromboli 3**	**38.804634**	**15.168529**	**0.403**	**ICP**
**SEA**	**Vulcano 1**	**38.421679**	**14.927802**	**0.464**	**ICP**
**SEA**	**Vulcano 2**	**38.413465**	**14.989952**	**0.100**	**ICP**
**SLOVAKIA**	**Starina**	**49.05**	**22.267**	**0.042**	**PIXE**
**SLOVENIA**	**CELJE**	**46.236**	**15.268**	**0.122**	**ICP**
**SLOVENIA**	**Deskles**	**46.052499**	**13.614876**	**0.100**	**ICP**
**SLOVENIA**	**ISKRBA**	**45.561**	**14.858**	**0.067**	**ICP**
**SLOVENIA**	**Ljubljana**	**46.051**	**14.506**	**0.130**	**ICP**
**SLOVENIA**	**Maribor**	**46.558**	**15.646**	**0.258**	**ICP**
**SLOVENIA**	**NOVA GORICA**	**45.956**	**13.643**	**0.143**	**ICP**
**SLOVENIA**	**PTUJ**	**46.419**	**15.869**	**0.154**	**ICP**
**SLOVENIA**	**Zerjav**	**46.485**	**14.87**	**0.149**	**ICP**

SLOVENIA	Kanal ob Soči	46.085	13.634	0.235	XACT
SPAIN	San Fernando	36.462	-6.218	0.333	ICP
SPAIN	Bailén	38.093	-3.784	0.741	ICP
SPAIN	Campus Univ El Carmen	37.272	-5.925	0.602	ICP
SPAIN	Carranque	36.72	-4.43	0.574	ICP
SPAIN	Granada Norte	37.197	-3.608	0.766	ICP
SPAIN	La Línea	36.159	-5.348	0.369	ICP
SPAIN	La Rabida	37.199	-6.932	0.665	ICP
SPAIN	Lepanto	37.894	-4.768	0.770	ICP
SPAIN	Matalascañas	37.016	-6.57	0.498	ICP
SPAIN	Mediterráneo	36.845	-2.457	0.544	ICP
SPAIN	Moguer	37.282	-6.834	0.927	ICP
SPAIN	Nerva	37.696	-6.55	0.819	ICP
SPAIN	Plza Castillo	36.997	-1.895	0.486	ICP
SPAIN	Príncipes	37.377	-6.004	0.678	ICP
SPAIN	Puente Mayorga	36.183	-5.387	0.382	ICP
SPAIN	Bailen	38.093	-3.784	0.819	ICP
SPAIN	Barcelona	41.387	2.115	0.256	ICP
SPAIN	Gijon	43.547	-5.704	0.329	ICP
SPAIN	Granada	37.164	-3.605	0.615	ICP
SPAIN	Madrid Ensanche Aguirre	40.422	-3.682	0.710	ICP
SPAIN	Madrid Ensanche Vallecas	40.373	-3.612	0.973	ICP
SPAIN	Manlleu	42.003	2.287	0.375	ICP
SPAIN	Montsec	42.051	0.73	0.022	ICP
SPAIN	Montseny	41.767	2.35	0.183	ICP
SPAIN	Villanueva del Arzobispo	38.174	-3.005	0.521	ICP
SPAIN	Huelva	37.262	-6.943	0.759	XACT
SWEDEN	Aspvreten	58.8	17.383	0.011	PIXE
SWITZERLAND	Basel	47.541	7.583	0.107	ICP
SWITZERLAND	Bern	46.951	7.441	0.184	ICP
SWITZERLAND	Magadino	46.16	8.934	0.160	ICP
SWITZERLAND	Payerne	46.813	6.944	0.104	ICP
SWITZERLAND	Zurich	47.378	8.53	0.088	ICP/XACT
SWITZERLAND	Payerne	46.813	6.945	0.021	ICP
SWITZERLAND	Dübendorf	47.403	8.613	0.262	XACT

Table S2 | Slopes and intercept (with corresponding uncertainties, defined as one standard deviation for a 10000-round bootstrap) for the comparison of the zero intercept (forced origin) and intercept models.

specie	forced origin slope	linear regression slope	linear regression intercept
**Fe**	0.85±0.052	0.81±0.14	0.11±0.23
**Ca**	1.58±0.099	1.46±0.29	0.29±0.47
**Si**	2.61±0.033	2.34±0.16	0.09±0.25
**Ti**	0.068±0.003	0.069±0.013	-0.016±0.022

Table S3 | Model variables used in this study.

Variable	Resolution (km)	Type	Source
**10-meter wind speed**	5.5	reanalysis	CERRA sub-daily regional reanalysis
**10-meter wind direction**	5.5	reanalysis	CERRA sub-daily regional reanalysis
**2-meter temperature**	5.5	reanalysis	CERRA sub-daily regional reanalysis
**total precipitation**	5.5	reanalysis	CERRA sub-daily regional reanalysis
**Dust aerosol optical depth at 550 nm (duaod550)**	83.325	reanalysis	CAMS global reanalysis (EAC4)
**DREAM**	36.663	forecast	WMO Barcelona Dust Regional Center

Table S4 | Land use variables used in this study.

LAND USE VARIABLE/CATEGORIES	SOURCE	SPATIAL RESOLUTION	TEMPORAL COVERAGE	COMMENTS
**LAND USE CATEGORIES: URBAN GREEN, NATURAL GREEN, WETLANDS, AGRICULTURE, PORTS, AIRPORTS, URBAN FABRIC, SNOW ICE, ROADS RAILS, WATER, BARREN LAND, INDUSTRIAL, TOTAL BUILD-UP**	CORINE Cover version 20	100m	2012 and 2018	Land use reported as percentage coverage for a circular buffer range of 1000m radius

**ELEVATION**	Altitude 90 m SRTM DTM v4	90m	N/A	Elevation reported for a circular buffer range of 1000m radius
**POPULATION DENSITY**	GEOSTAT population grid	1 km	2011	Population density reported for a circular buffer range of 1000m radius
**IMPERVIOUS DENSITY (IMD) – PERCENTAGE OF SEALED AREA**	Copernicus services	100m	2009, 2012, 2015, 2018	IMD reported as a percentage coverage for a circular buffer range of 1000m radius
**ROADS IN 3 ROAD CATEGORIES ROAD CLASS1 (MOTORWAY, MOTORWAY LINK, TRUNK AND TRUNK LINK), ROAD CLASS2 (PRIMARY AND PRIMARY LINK), ROAD CLASS 3 (SECONDARY, SECONDARY LINK, TERTIARY, TERTIARY LINK AND RESIDENTIAL)**	Open street maps	25m	2020	Road coverage reported as total meters for a circular buffer with a radius of 200, 500, 100 and 2000m

Table S5 | Summary statistics between the dust models

	**RF dust leave-1 year out**	**RF dust leave-1 station out**	**DREAM dust Comparison**
**Pearson r**	0.54	0.52	0.49
**R^2**	0.29	0.27	0.24
**RMSE**	6.0	6.2	12.6
**MAE**	2.8	3.2	5.0
**Fractional Bias (FB)**	30.1	38.2	-109.0

Table S6 | Increase in all cause, cardiovascular and respiratory mortality using the relative risks reported in Stafoggia et al. 2016 and Pouri et al. 2024. For the case of all-cause mortality Pouri et al. 2024 report the IR for PM_{10} during dust day rather than desert PM as Stafoggia. The impact of the uncertainty in mean dust exceedance concentrations on the health outcomes is used to compute the uncertainty of those outcomes.

	**Stafoggia et al. 2016**	**Pouri et al. 2024**
**Natural/All-cause**	0.67%±0.02	1.97%±0.06
**Cardiovascular**	1.14%±0.03	1.07%±0.03
**Respiratory**	1.33%±0.04	1.06%±0.03

Table S7 | Summary performance statistics of the RF dust model for different years

**Year Fold**	**# of datapoints**	**Pearson r**	**R²**	**RMSE**	**MAE**	**Fractional Bias (FB)**
**2012***	664	0.49	0.24	5.44	2.84	-0.62
**2013**	2678	0.48	0.24	4.76	2.61	23.30
**2014**	2697	0.50	0.25	6.71	3.18	20.58
**2015**	1903	0.51	0.26	6.30	3.00	17.59
**2016**	1285	0.58	0.33	10.11	3.79	31.09
**2017**	2163	0.58	0.33	5.75	2.69	30.56
**2018**	1860	0.58	0.33	6.27	3.11	24.35
**2019**	2679	0.52	0.27	4.95	2.57	34.49
**2020**	2137	0.60	0.36	5.14	2.35	21.06
**2021**	1066	0.53	0.28	6.14	3.21	29.89

*not included in final model

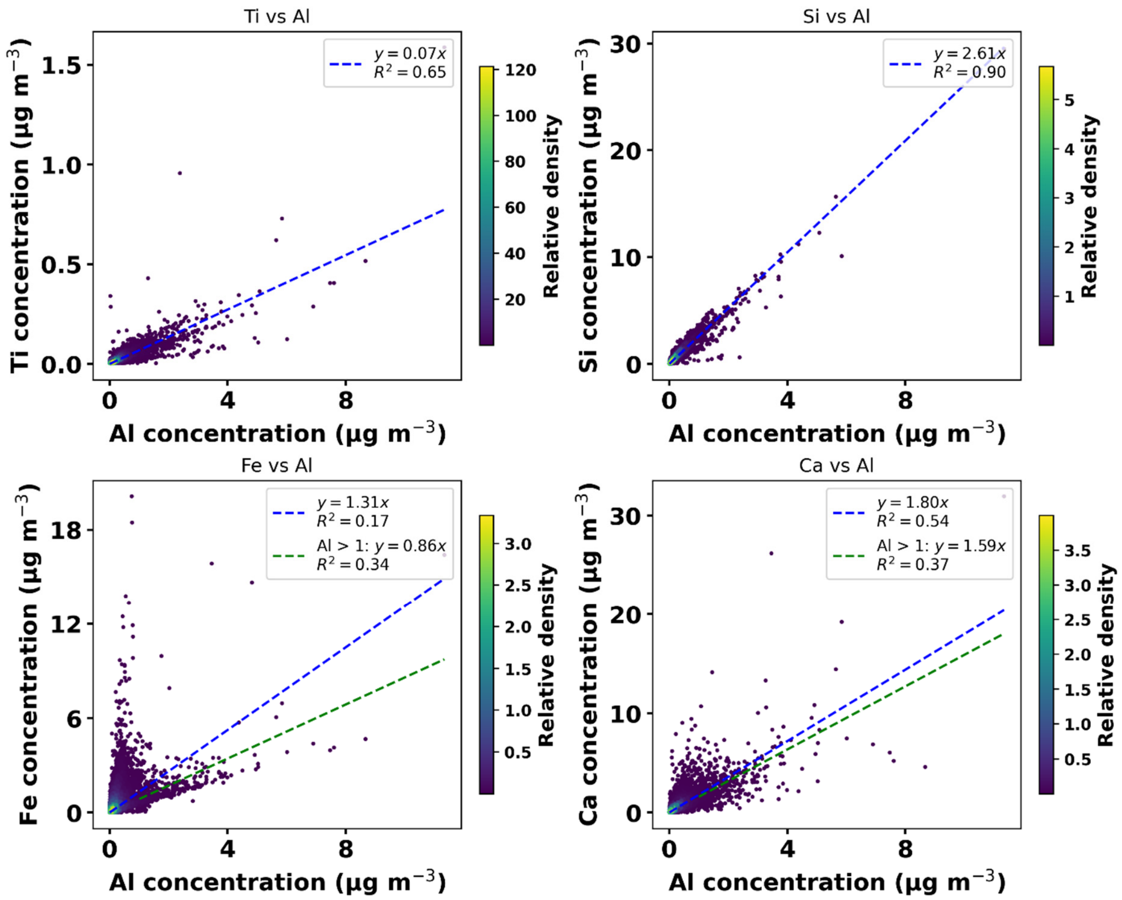

Fig S1 | Correlations between Al and Ti (a), Si (b), Fe (c) & Ca (d). For c & d, linear regression slopes are also shown for the subset of the data where Al is greater than 1 µg m^{-3} (green line).

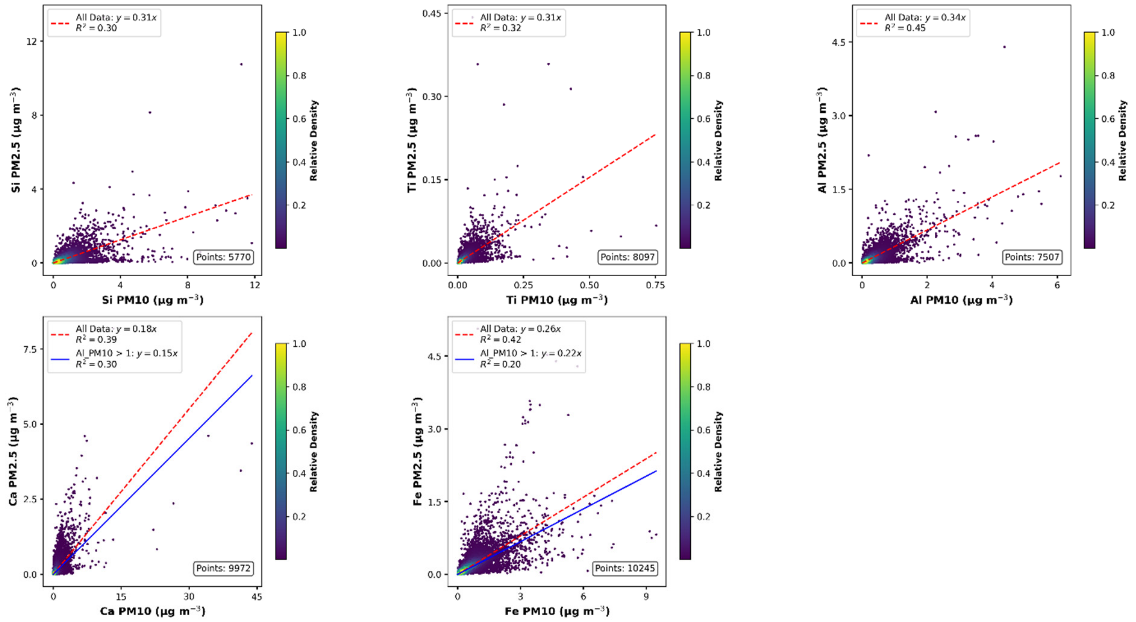

Fig S2 | Scatter plots of PM_{10} vs $PM_{2.5}$ metals for Si (a), Ti (b), Al (c), Ca (d) and Fe (e) for a subset of the database (18 sites), where co-located $PM_{2.5}$ & PM_{10} chemical analyses exist. A $PM_{2.5}$:PM_{10} concentration ratio of dust, equal to 27.7%, was obtained via inserting the $PM_{2.5}$:PM_{10} concentration ratios for the elements (for Ca and Fe at Al>1 μg/m^3) in the equation 2.2Al+2.49Si+1.63Ca+1.94Ti+ 2.42Fe.

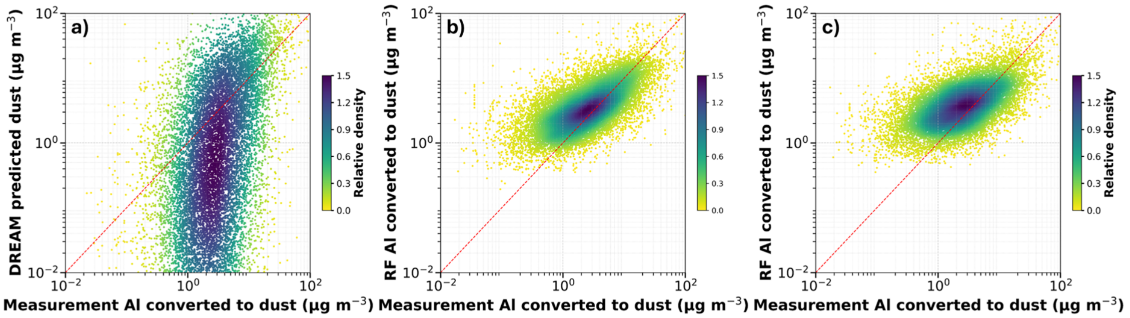

Fig S3 | Comparison between measured dust and DREAM (a), Random Forest leave-1-year-out (b) and leave-1-station-out (c). Statistical measures for this figure are shown in table S5.

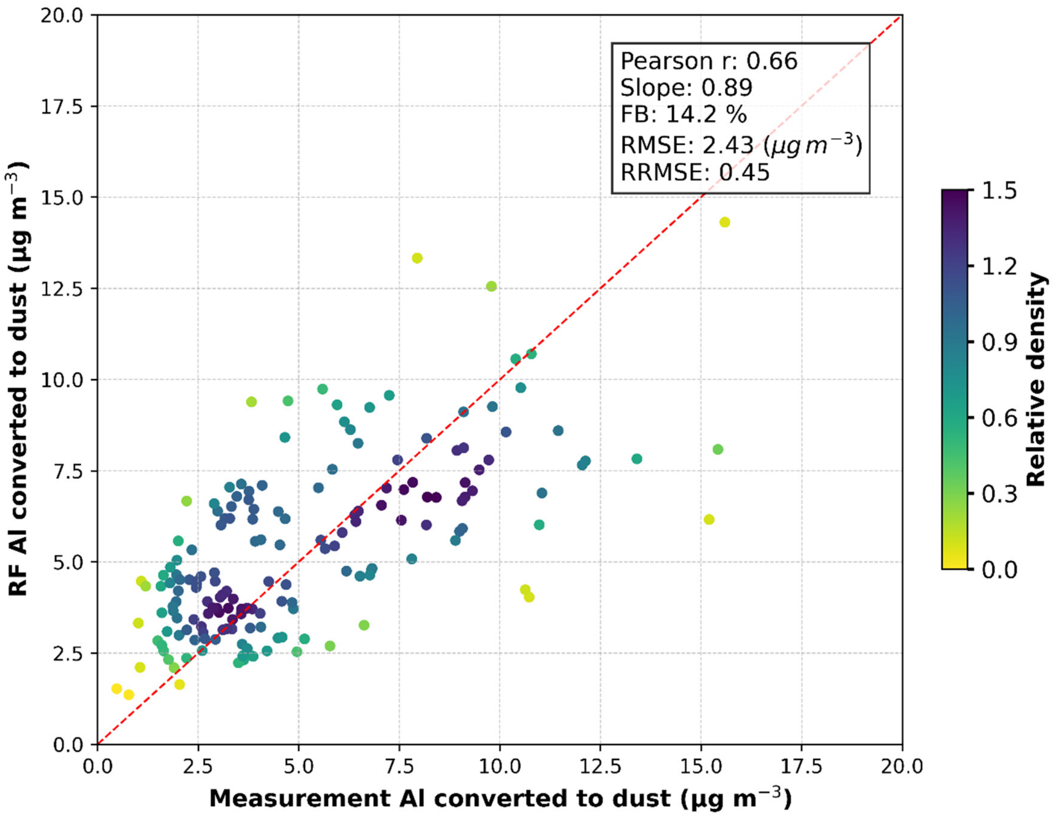

Fig S4 | Leave-1-out results aggregated on a yearly basis, with relevant statistical metrics (Pearson correlation coefficient r, linear regression slope, fractional bias FB, root mean square error RMSE and relative root mean square error RRMSE).

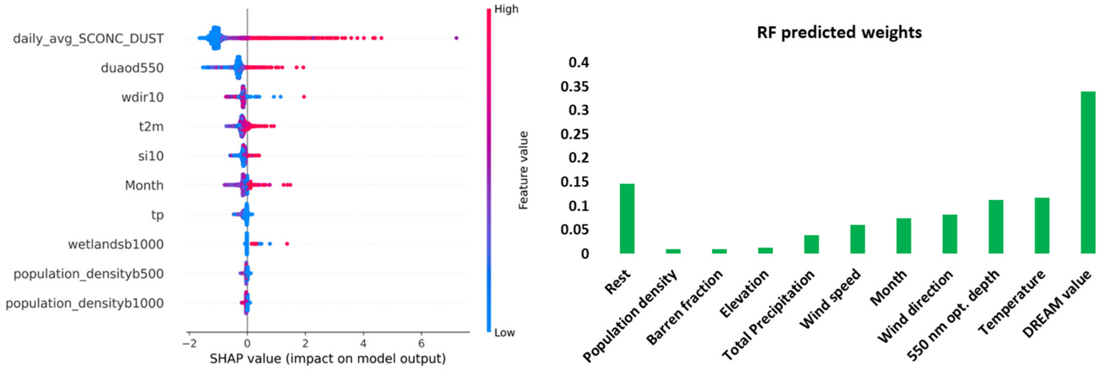

Fig S5 | SHAPLEY plot for the top 10 in importance model variables (a), and Random Forest (RF) predicted variable weights (b).

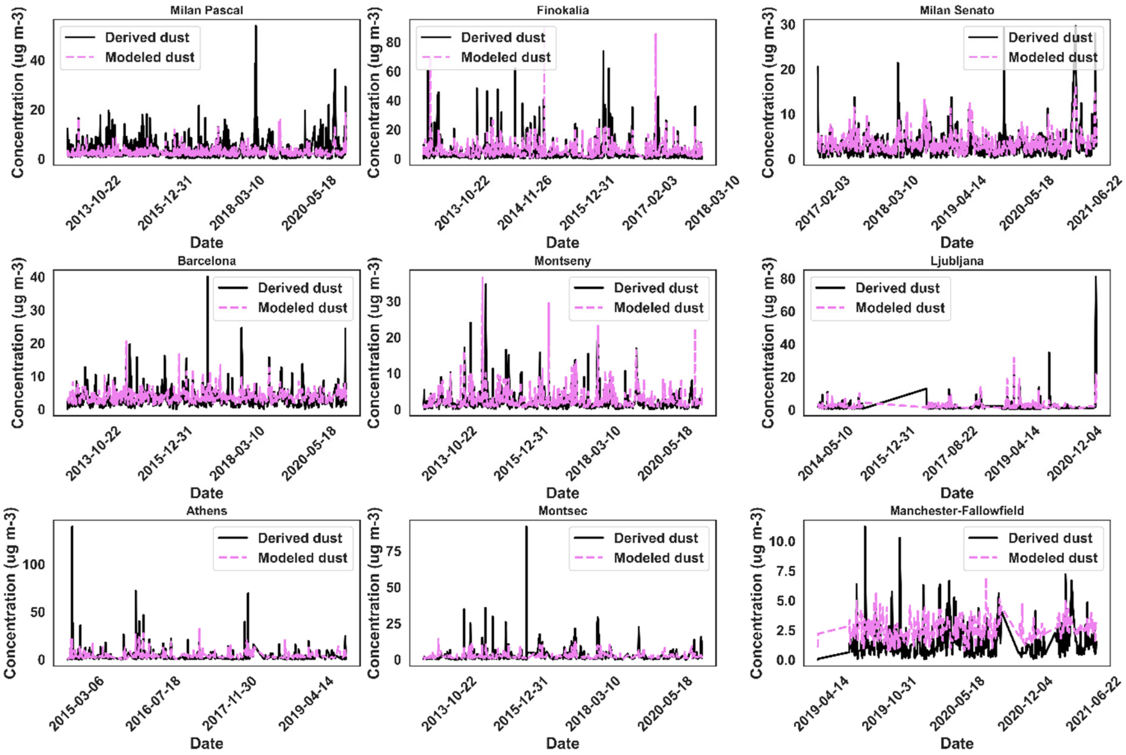

Fig S6 | Timeseries for the sites with largest number of daily measurements in the dataset, for the measurement derived dust (solid line) and the Random Forest (RF) model derived dust (dashed pink line).

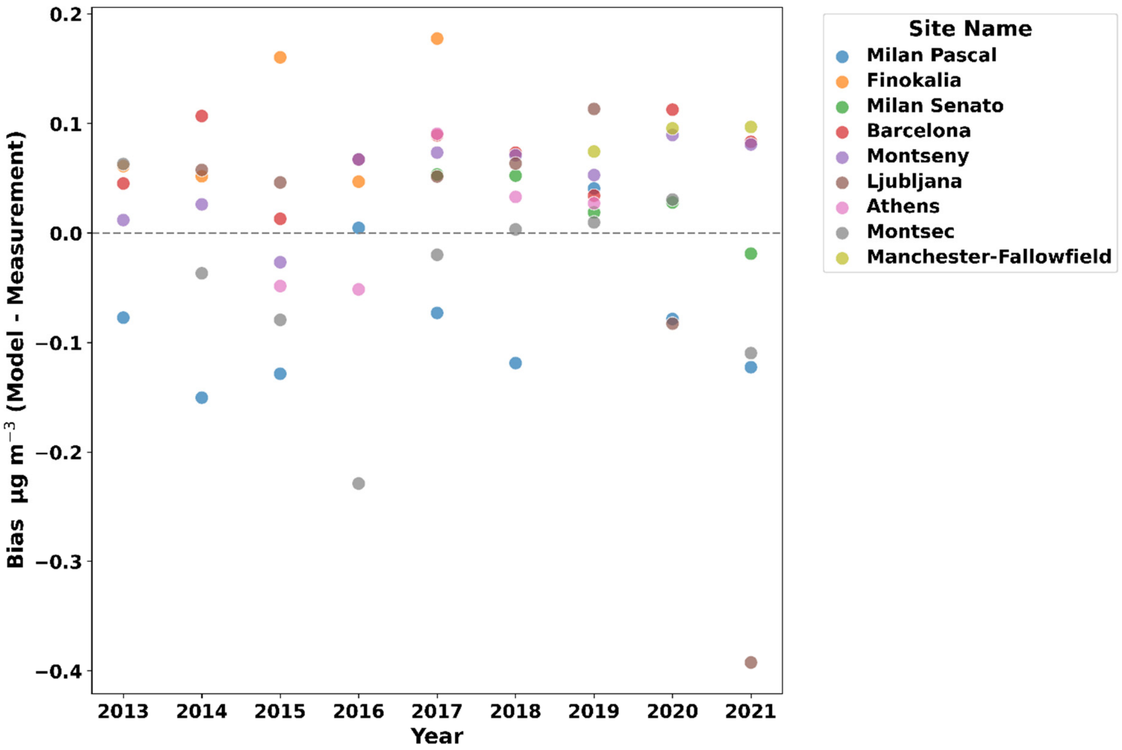

Fig S7 | Temporal bias (yearly model average value minus yearly measurement value) for the stations with the largest number of daily measurements (same as in Fig. S5).

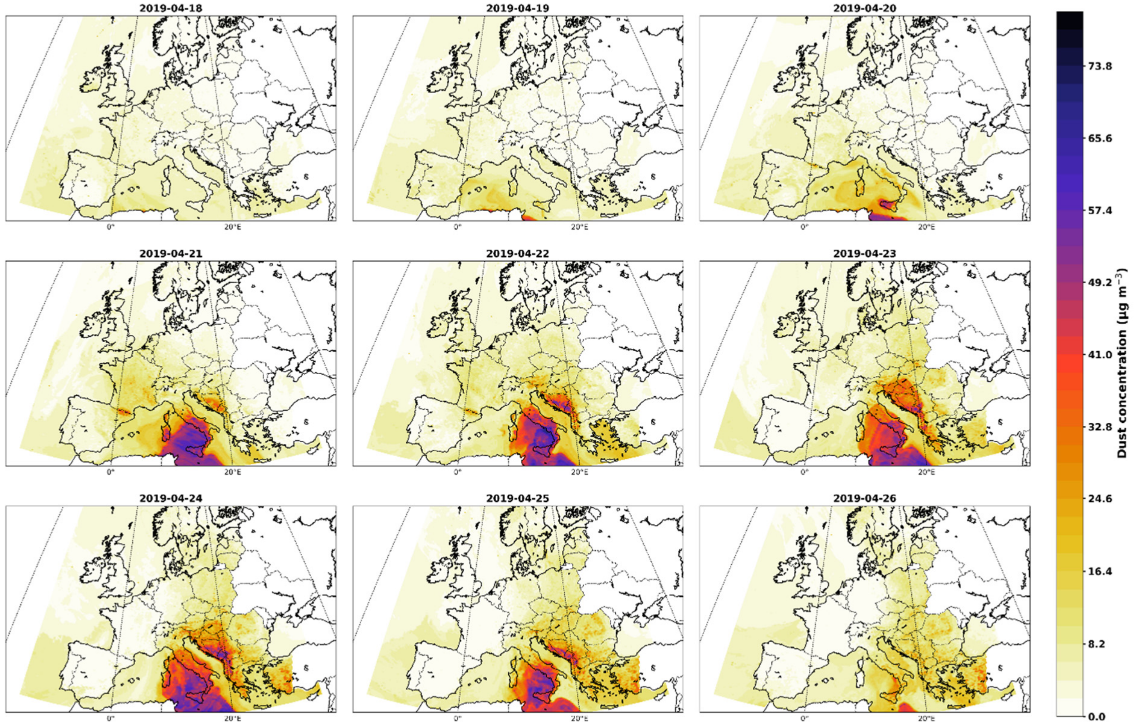

Fig S8 | Random forest–predicted dust concentrations (μg m^{-3}) showing the simulated transport trajectory of Saharan dust during the extreme event affecting the Balkans, as reported by Peshev et al. (2021), for the period 18 April 2019 (one day before the episode's onset) to 26 April 2019 (episode end).

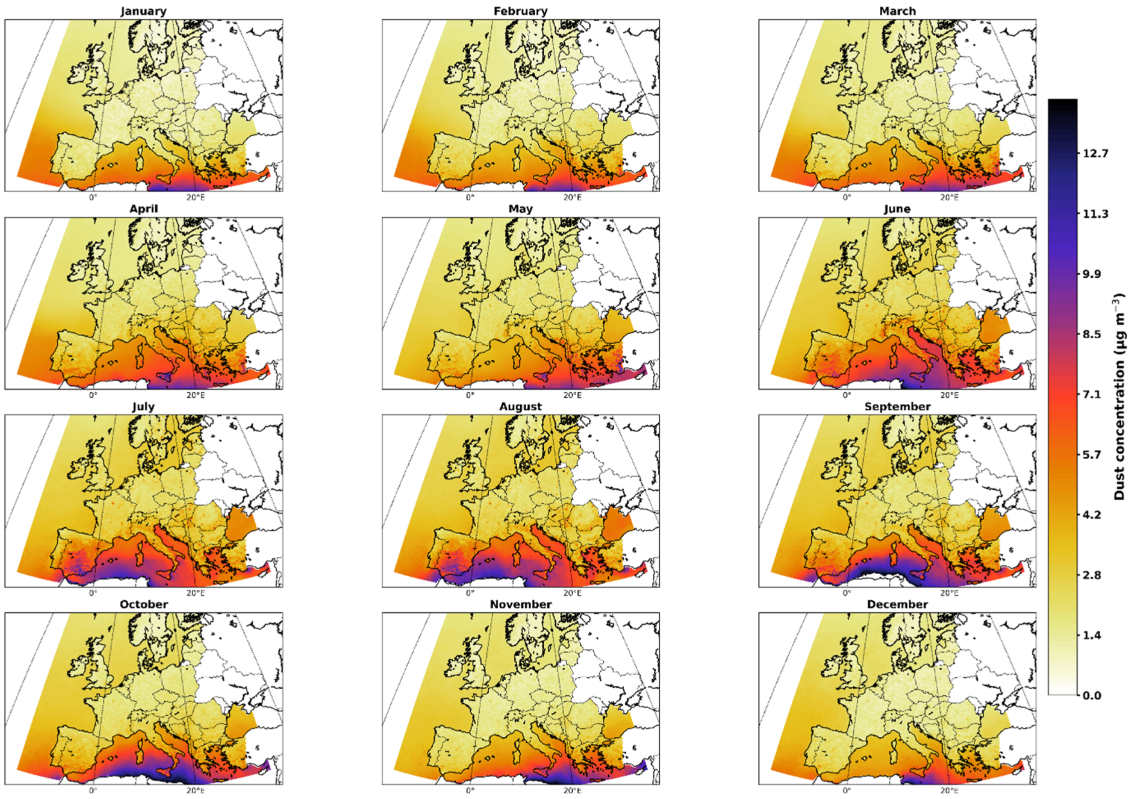

Fig S9 | Seasonal variation of random forest–predicted dust concentrations (μg m^{-3}), shown as 10-year monthly averages for the study domain.

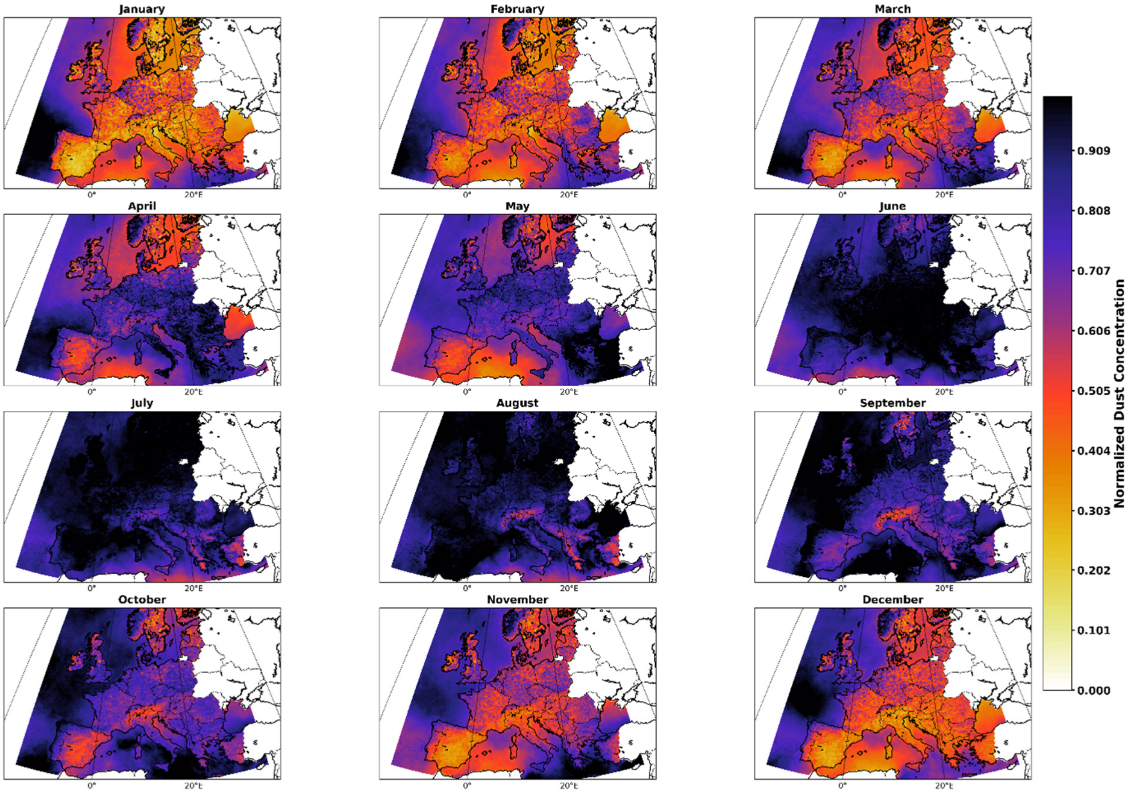

Fig S10 | Seasonal variation of random forest–predicted dust concentrations, expressed as monthly averages relative to the yearly maximum monthly concentration of the respective grid cell for the study domain.

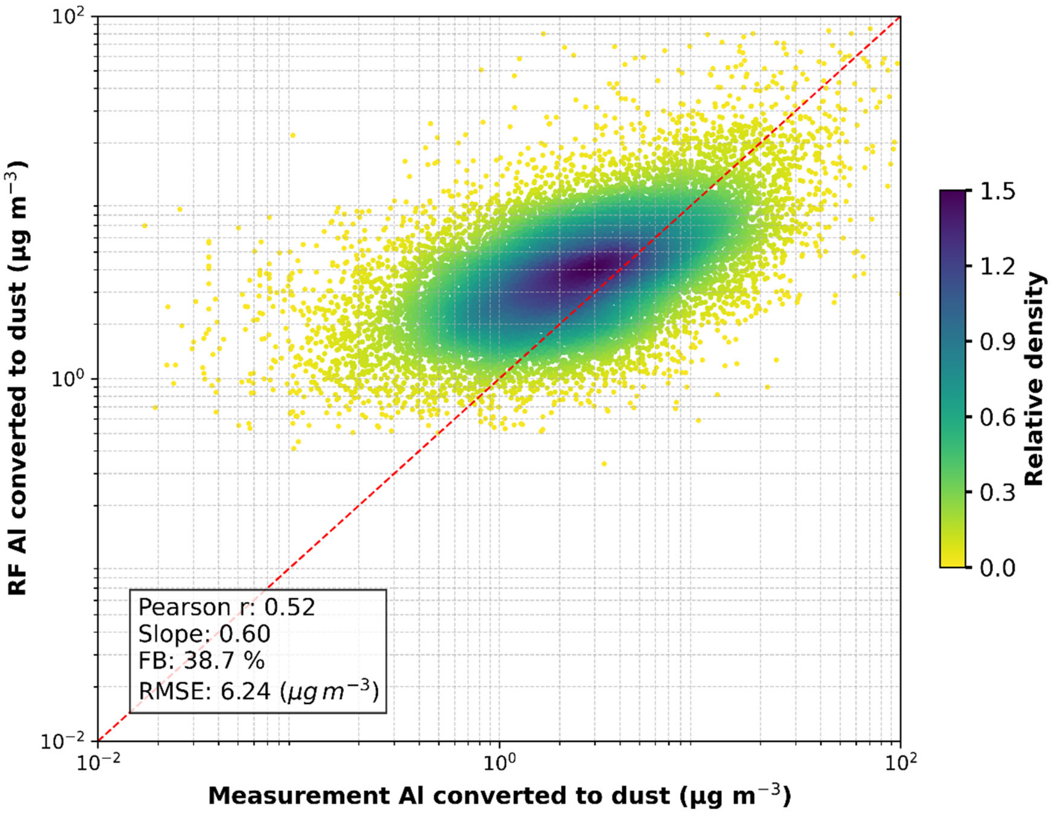

Fig S11 | Model training results when all available soil variables from the European Soil Database (https://esdac.jrc.ec.europa.eu/content/european-soil-database-v2-raster-library-1kmx1km, last accessed: 05.11.2025) are added, including soil moisture & erodibility. Also shown are relevant statistical metrics (Pearson correlation coefficient r, linear regression slope, fractional bias FB & root mean square error RMSE). Results are shown in a logarithmic scale and the color represents the relative density of measurements.

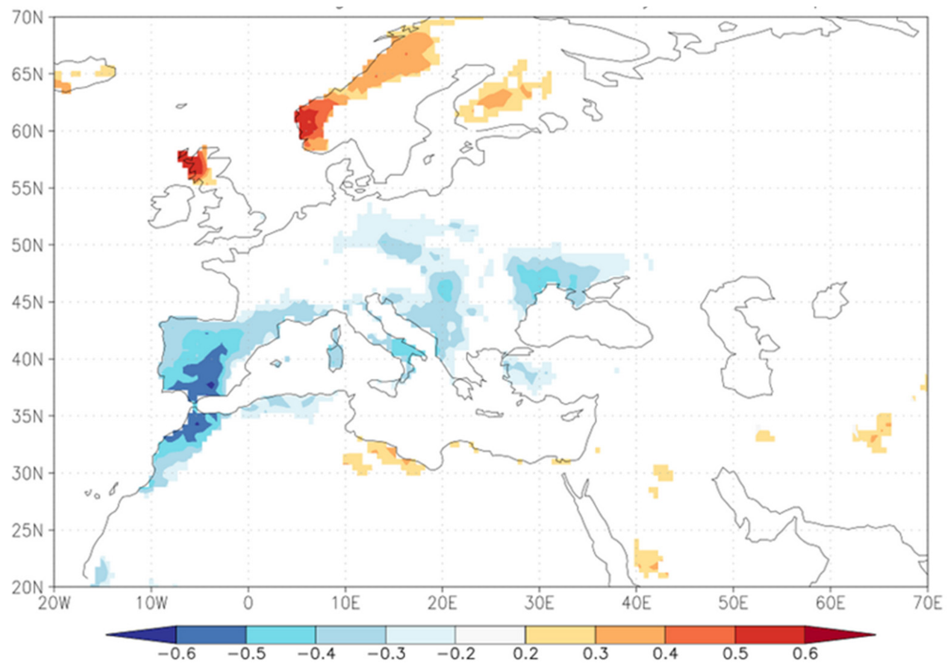

Fig S12 | Spatiotemporal correlation between December-March averaged North Atlantic Oscillation (NAO) and annual Palmer Drought Severity Index (PDSI) between 1901 and 2021 (from KNMI Climate Explorer. c2022. de Bilt: Koninklijk Nederlands Meteorologisch Instituut (KNMI); [last accessed 2025 Feb 21]; https://climexp.knmi.nl/start.cgi.)

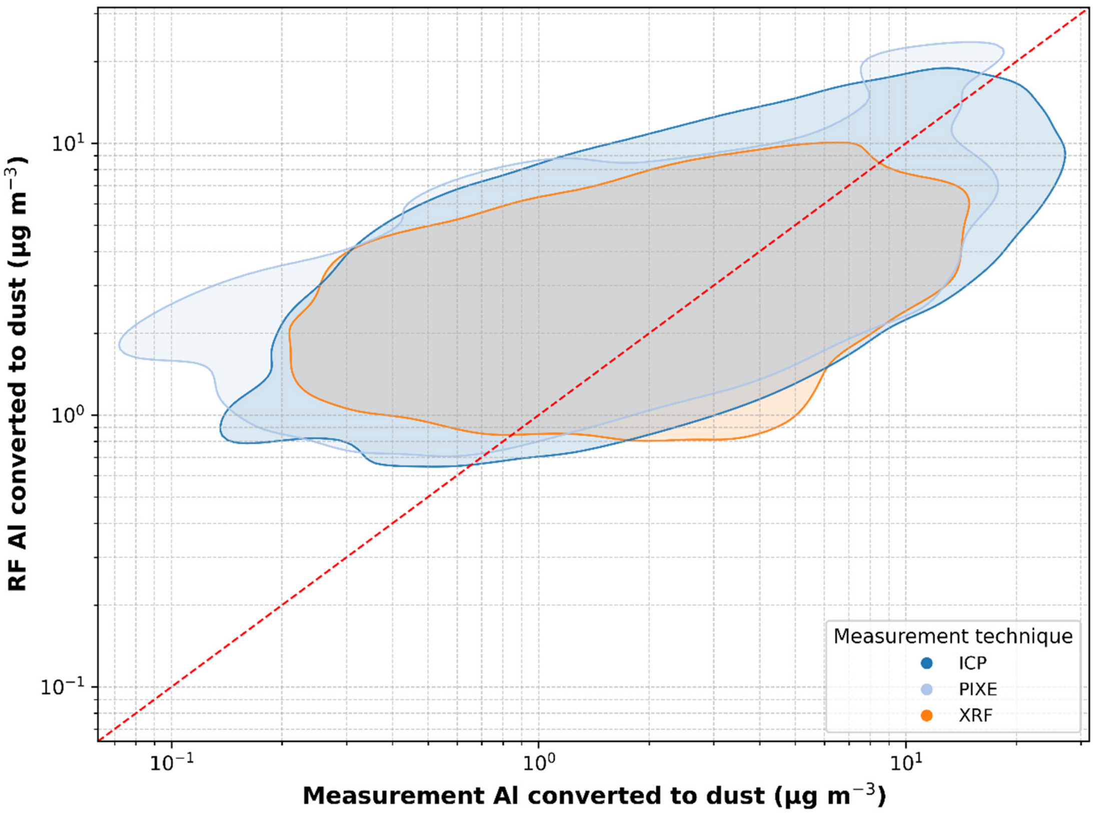

Fig S13 | Density plot for 90% of predictions, grouped by technique, for the leave-1-out results.

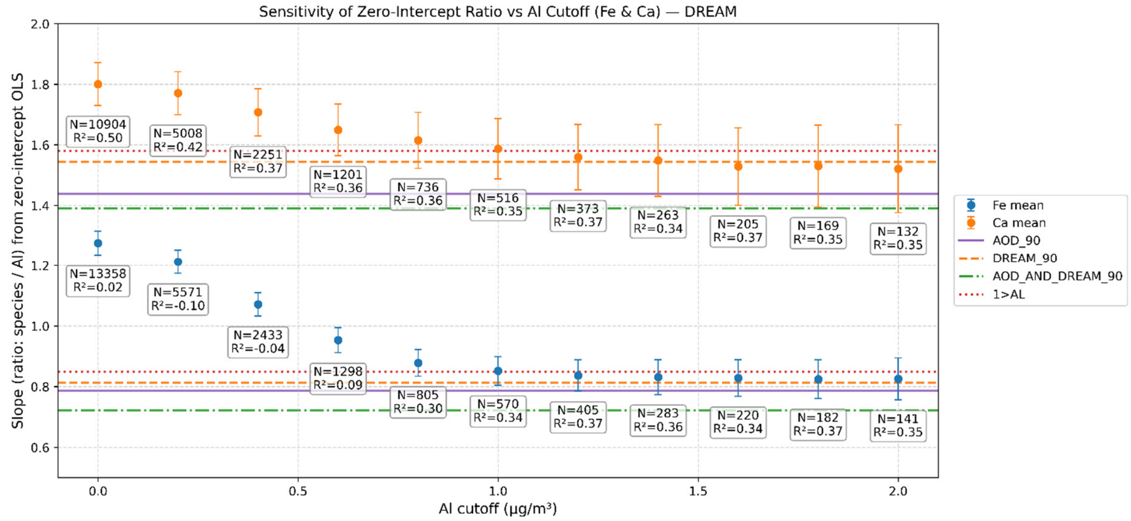

Fig S14 | Sensitivity of the mass-based elemental ratios of Fe/Al & Ca/Al for different Al cutoffs. The ratios for the cases where Al>1μg m^{-3}, and for the 90th percentile of DREAM surface dust concentration, dust AOD and both at the same time are shown as horizontal lines.

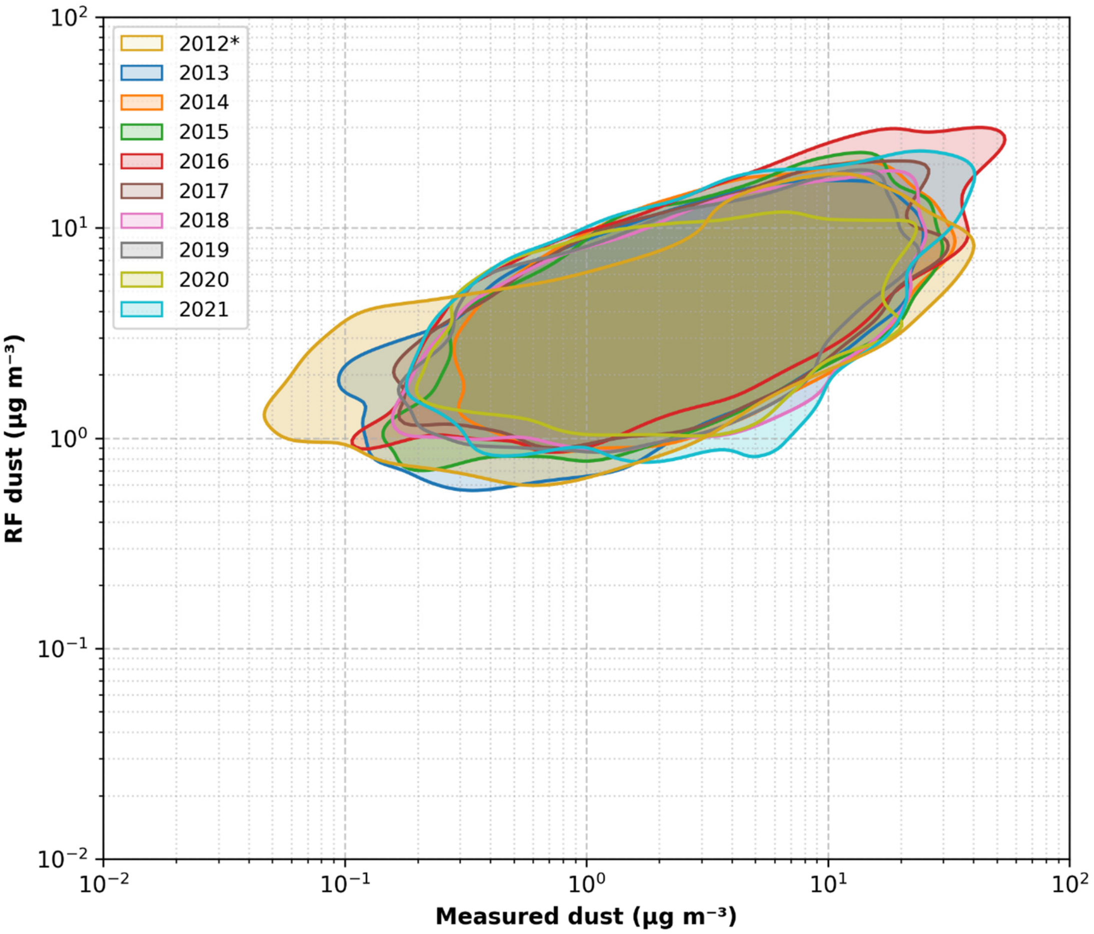

Fig S15 | Density plot for 90% of predictions, grouped by year, for the leave-1-year-out results (*2012 not included in the final model).
