# Supplementary material for: Rising dust pollution across Europe in a changing climate
Source: Nature. 2026 Jul 15;655(8123):647–54. doi: 10.1038/s41586-026-10743-w (PMC13372676; doi:10.1038/s41586-026-10743-w)
Supplement: Supplementary file 2 — Peer Review file [file 41586_2026_10743_MOESM2_ESM.pdf]

nature portfolio

Peer Review File

Rising dust pollution across Europe in a changing climate

Corresponding Author: Dr Petros Vasilakos

Any redactions in this file are there to maintain patient confidentiality, the confidentiality of unpublished data, or to remove third-party material.

This file contains all reviewer reports in order by version, followed by all author rebuttals in order by version.

Version 0:

Reviewer comments:

Referee #1

(Remarks to the Author)
This manuscript aims at both quantifying the short-term (2012-2021) variability of dust concentrations over Europe and also the long-term trends over the period from 1750 to present.
The novelty consists of the aggregation of different sources of information that are:
atmospheric mineral data from 103 rutal and urban sites and 18500 daily measurements in total, machine learning together with ice core records and dust long-term trends in European dust concentrations. The authors also discuss the climatic drivers associated with this variability.
I find this work is original and significant.
It helps quantifying the PM2.5 and PM10 baseline caused by dust over Europe (one of the priorities when assessing particulate concentrations over the region) and the methods used are convincing.
The conclusions drawn from this study are reliable with the following caveat.

With regards to the long term trends in Ca2+ estimated to have rise by 110% from the preindustrail (1750-1850) to the past decade (2010-2020), the authors should word it carefully as it reflects the measurements of 3 cores from a single geographical location in the Alps which is Colle Gnifetti. I regard this trend as credible since analysis at more sites published by Kok et al. 2023 (reference 5 in this manuscript) published in NEE, also discuss a similar albeit less intense long-term trends over the XXth century.

I have several minor comments that could help still improve the manuscript that is already in good form. In particular, good care was put in having a good description in the Methods.

Minor points:
- It would be useful to give the correlation coefficients associated with Fig. S2a and S2b. This would help you make the point that the Random Forest (RF) algorithm predicts the dust fields more precisely than a state-of-the-art model such as the DREAM model.

- Fig. captions S6 and S7 need to be more precise and give an exact description of what is shown.

- I understood that Figs. S8 and S9 are the results of the RF algorithm, it should be clearly stated in the Figure caption of these two figures.

- THe figure caption for Fig. S7 is incomplete. Please write full sentences to describe what is depiected in this figure

- Showing 2 decimals for the mean concentration of dust (ug m-3) is a bit provocative. You have an incredible confidence in the RF alrorithm if you trust it to the second decimal. Already having one decimal would be optimistic, I would not show a second decimal.

- In all the occurrences of the following unit: ug m-3 year-1 you have a space missing between m-3 and year-1 please correct all such occurrences

- Please replace 'yearly change in concentration during background' with 'yearly concentration change for background conditions'

- Figure S10: Avoid acronyms in Figure captions, a figure caption should be understood without reading the main text, hence write 'Palmer Drought Severity Index' instead of 'PDSI'.

- Same applies for Figure S4, RF can stand for many things and is generally used for Radiative Forcing when considering aerosols, here you to refer to Random Forest Algorithm.

- Line 378-379 you could indicate to the reader that you are giving the set of parameters used to optimize the Random Forest Algorithm in the lines below.

Referee #2

(Remarks to the Author)
In this continental scale study, the authors developed a random forest model using measured dust meta ratios to estimate daily dust PM10 concentrations in Europe from 2012 to 2021. The authors then reported the temporal trend and spatial characteristics of transported dust events, their impact on regional air quality, and the associated mortality burden. As dust transported from Africa pose an increasing threat to air quality and public health in Europe, this study is timely and policy relevant. While the results presented here may appeal to readers interested in understanding the health impact of environmental risk factors driven by climate change such as transported dust, there are major technical concerns regarding the dust PM10 model development and performance. These validity of the technical approach as well as the quality of the predicted dust PM10 concentration put the robustness of the study findings in doubt. This manuscript in its current form is not suitable for publication. Specific comments are provided below.

Major comments:
• Elemental ratios for dust PM10 concentration reconstruction. Dust PM10 concentrations in this study, as in many other previous studies, are reconstructed using element concentrations and elemental ratios. Specifically, Al was selected as the key tracer for transported dust and zero intercept linear regression models were built to estimate the Si:Al, Ti:Al, Ca:Al, and Fe:Al ratios. This step is crucial to the values and quality of the estimated dust PM10 concentrations. There are multiple concerns here. First, the authors state that Al and Ti are ideal tracers for transported dust detection. However, the article that they cite is from 2001. There are more current citations regarding this, including some that indicate the use of multiple tracers (Al, Fe, Th, Ti) allows for more accurate estimates of dust and less uncertainty. Second, authors state that these elemental ratios align well with dust transport and Saharah source estimates from Chiapello et al. from 1997. It is likely that there are more recent reports of these values and authors should provide additional information regarding if and or how the ratios from the 1997 article are still acceptable to use.
Put these concerns aside, three technical issues emerge that put the validity of the simplified approach adopted in this study in doubt.
a. First, as shown in Fig 1b, each of the elemental ratios have a non-trivial scatter around the mean values. This uncertainty has major impacts on the estimated dust PM10 concentrations (line 160 - 164) but was not mentioned in any of the following analyses. While the authors state that their approach aligned well with some previously published material, it is important to include how different these ratios are in comparison and whether utilizing these ratios introduces any bias or uncertainty into the estimates of dust concentration.
b. Second, the authors provided no justification for the choice of zero intercept regression. It has the advantage of giving a neat elemental ratio, but usually the fit is worse than a standard simple linear regression with both intercept and slope. This tradeoff needs to be discussed in the paper.
c. Finally, the regression models were developed only for high Al concentrations (line 119). What is the rational behind this choice of 1 g/m3? How would the estimated ratios change with this selected threshold? This crucial sensitivity analysis is missing. In addition, the elemental ratio conversion yields dust estimate of 12.3 times the Al concentration (line 163). This translates into a dust PM10 concentration of above 12.3 g/m3 when the elemental ratios were estimated. According to Fig 4c, this level is near the maximum mean dust concentration across the entire study domain during the study period. In other words, the elemental ratio conversion does not apply to most dust episodes. The choice of this selected Al concentration threshold of 1 g/m3 doesn’t appear to be appropriate.

2. Random forest model for dust PM10 concentration prediction. A random forest model was trained to predict Al concentrations in the entire study domain in order to estimate dust PM10 concentrations. Authors used land use variables to inform the model, but there is little discussion regarding previously published works that point to these specific variables as important considerations to include in the model. Also, there is little discussion regarding as to whether variables like soil erodibility, soil moisture, and terrain roughness were considered and whether any seasonal vegetation changes were accounted for. There are also technical concerns related to model performance.
a. As shown in Fig S2, the cross-validation results indicate that RF model overestimate converted dust concentration by a factor of 2 or more at low to medium levels (e.g., below 10 g/m3). This bias becomes more pronounced at lower concentrations, suggesting that its ability to accurately characterize dust concentrations deteriorates with latitude. Without additional correction, its ability to detect subtle time trend is also questionable. For example, concentration increase for most of Europe was estimated at 0.05 g/m3 per year (line 211). No test results of the statistical significance of this trend were shown.
b. The authors point to Figure S4 as evidence that the model accurately depicts multi-year dust concentrations. While the figure illustrates the capture of the general trend, there seems to be substantial differences at times between the observed values and model estimates. There is no discussion regarding how this could impact the results or whether any correction or

calibration was done.
c. The authors claim that the RF model captures the plume paths and magnitude and point to Figure S6. However, it is difficult to determine this without a side-by-side comparison to the observed values. It is important to substantiate claim because authors suggest that the RF model is well-suited to analyzing dust in Europe and suggest that this model is superior to those previously published.

3. Health Impact assessment. As currently constituted, the health impact analysis is lacking detail and feels like an afterthought. To justify its inclusion, additional information is needed, including:

a. The authors mentioned that their information regarding all-cause mortality and hospitalizations comes from Stafoggia et al. 2016. Were any other sources of information considered? For example, how does the Stafoggia links to all-cause mortality compare with those from meta analyses such as that in Pouri et al, 2024 (https://doi.org/10.1016/j.scitotenv.2023.168945).
b. It is important to state that these estimated increases in risk are per 10 ug/m3 increase in exposure to dust PM10 and that the Stafoggia results are reported as lag 0-1 results for all-cause mortality and lag 0-5 results for respiratory hospitalizations. This is an important distinction to make, and authors need to more explicitly state how they accounted for this in their estimates.
c. The authors need to clarify where the information regarding adult vs. adolescent health impact comes from. From a cursory look at Staffogia et al. 2016, it seems that they separated out hospitalizations as individuals 0-14 and individuals aged 15 years and older. This delineation does not follow accepted definitions of adults vs. children or adolescents.
d. If there is truly a potential impact on these health outcomes, more information is needed regarding methods and whether there has been change over time, which would help to make the case for the link to climate change made by the authors in the beginning paragraphs.

Minor comments:
• The authors mention the use of data from multiple sources that were harmonized to a 10km x 10km spatial resolution, but very little detail is given regarding the specifics. Also, because authors include a health component, it is necessary to utilize methods for population weighting, which are not apparent from the text. Authors repeat the interpolation using a population weighting approach and more complete methods need to be included in the supplement.
• The authors state that the RF model effectively captures major and minor dust intrusions, but little information is given regarding how they assess this. How was this determined?
• The article also gives information regarding the PM2.5 to PM10 ratios. However, there is little explanation of how this pertains to the model or was used to inform estimations. Please justify the inclusion of information regarding the processing time for the models. This seems out of place and should be excluded from the main text of the manuscript.
• Throughout, authors do not spell out multiple abbreviations and acronyms. Since Nature reaches a larger audience, it is important to introduce the elemental abbreviations as well as the acronyms for DREAM, CHIMERE, CORINE, etc.
• Figure 1b seems unnecessary since this information is easily gleaned from the text and should be excluded or moved to the supplement.
• Throughout, some of the color schemes are either difficult to read or do not reflect the underlying data. For example, the scheme used in Figure S6, S7, and S9 makes it difficult to see the country boundaries due to the use of black as the extreme high values.

Referee #3

(Remarks to the Author)
I co-reviewed this manuscript with one of the reviewers who provided the listed reports.

Referee #4

(Remarks to the Author)
I co-reviewed this manuscript with one of the reviewers who provided the listed reports.

Referee #5

(Remarks to the Author)
The manuscript entitled "Rising Dust Pollution Threatens European Air Quality in a Changing Climate" synthesizes multi-source data to develop a random forest (RF) model characterizing dust events across Europe. It further investigates the underlying mechanisms and potential impacts of dust uplift. While the findings provide valuable insights that could inform dust monitoring and mitigation strategies, several critical scientific gaps and methodological uncertainties remain. These issues must be adequately addressed before the study can be considered for publication in Nature.
1. Studies on the impact of climate change and dust on Europe has been relatively thorough, and similar conclusions are also quite abundant. The author needs to clarify whether the innovation of this study lies in the methodology or in the cognition. And highlight the key points where this study differs from or improves upon previous research. Such as:
Shao, Y., M. Klose, and K.-H. Wyrwoll (2013), Recent global dust trend and connections to climate forcing, J. Geophys. Res. Atmos., 118, 11,107–11,118, doi:10.1002/jgrd.50836.
Vautard, R., Bessagnet, B., Chin, M., and Menut, L. (2005). On the contribution of natural Aeolian sources to particulate matter concentrations in Europe: Testing hypotheses with a modelling approach. Atmos. Environ. 39, 3291-3303.

10.1016/j.atmosenv.2005.01.051.
Li, Tiantian et al (2025). Sand and dust storms: a growing global health threat calls for international health studies to support policy action, The Lancet Planetary Health, Volume 9, Issue 1, e34 - e40.
Ying Dai, Peter Hitchcock, Natalie M. Mahowald,et.al. Stratospheric impacts on dust transport and air pollution in West Africa and the Eastern Mediterranean. Nature Communications, 2022.7744
Clifford, H. M., Spaulding, N. E., Kurbatov, A. V., More, A., Korotkikh, E. V., Sneed, S. B., et al. (2019). A 2000 year Saharan dust event proxy record from an ice core in the European Alps. Journal of Geophysical Research: Atmospheres, 124, https://doi.org/10.1029/2019JD030725

2. The study exhibits significant methodological gaps, as it lacks several critical auxiliary analyses to substantiate its conclusions. Key supporting evidence—such as quantitative simulations of dust emission intensity in the Sahara Desert, trajectory modeling of European air currents, and satellite remote sensing data—is either missing or insufficiently utilized. For example, while the authors attribute the rise in European dust levels to Sahara Desertification, this claim would be substantially strengthened by robust satellite-based evidence, which currently appears underdeveloped in the analysis.

3. Data Harmonization and Comparability: A critical methodological concern pertains to the comparability of chemical composition data across multiple sampling sites. While the manuscript briefly acknowledges this issue (Lines 363-365), stating that 'great care was taken in curating the database by flagging data below detection limits and working with data providers to ensure high data quality,' this explanation remains insufficiently detailed. Specifically, the following critical questions remain unanswered: (1) What systematic approaches were implemented to address measurement discrepancies arising from heterogeneous analytical techniques? (2) What specific calibration protocols, normalization procedures, or data harmonization methods were employed to ensure cross-site comparability? (3) Were any quality control metrics or uncertainty estimates applied to account for potential inter-instrument variability?

4. Scientific Rationale and Ratio Interpretation: Several aspects of the study design need further elaboration. (1) Why was aluminum (Al) selected as the target variable for dust estimation? How were other crustal elements, such as silicon (Si) and iron (Fe)? (2) The reliability of key elemental ratios—such as Si/Al, Fe/Al, Ca/Al, and Ti/Al—is questionable. It is striking that these ratios appear to fall within such narrow and consistent ranges across numerous stations and time periods. Furthermore, these ratios lack contextual comparison with source-region dust compositions. Although the authors cite Chiapello et al., certain ratios, such as Fe/Al and Ca/Al, differ substantially from those reported in that study. A more rigorous evaluation of the stability and representativeness of these ratios is required to validate the study’s conclusions.

5. This study aims to reflect the impact of dust events, using the RF model to simulate Al concentrations, and further calculate dust concentrations. Why not directly use the monitored Al concentration? What is the significance of using the RF model to re-estimate the Al concentration, just for spatial analysis? The author also compared their modeled concentrations with monitoring.

6. Model Evaluation and Uncertainty: The reliability and predictive accuracy of the RF model need to be more thoroughly demonstrated. Currently, the manuscript lacks quantified performance metrics (e.g., R^2, RMSE, MAE) in the “Model Performance” section. Moreover, Figure S4 suggests notable discrepancies between observed and modeled Al concentrations, particularly a tendency for the model to underestimate high values and overestimate low values. This pattern is especially evident during extreme dust episodes, which the model appears unable to capture effectively. These limitations should be explicitly discussed.

7. The use of DREAM-modeled dust as an input variable raises methodological concerns. Given that DREAM is driven by meteorological factors, and these same meteorological variables are also directly input into the RF model, there is a risk of information redundancy or collinearity. How did the authors address potential overlaps between direct and indirect representations of meteorological drivers?

8. Using spatio-temporal correlation analysis to investigate climate change's driving mechanisms and impacts on European dust. However, this methodological approach faces substantial critiques regarding its capacity to authenticate causal relationships and credibly elucidate underlying physical processes

Version 1:

Reviewer comments:

Referee #1

(Remarks to the Author)
The authors took into consideration the limitation for their study that I stated and added several sentences to the text to reflect it. All the minor improvements requested were addressed and I did not have methodological remarks contrary to the second reviewers that had many more requeststs for justification.
THerefore, the authors answered my comments.

Referee #2

(Remarks to the Author)
The authors have sufficiently addressed my comments and concerns with explanation and additional analysis. I don't have any additional comments.

Referee #3

(Remarks to the Author)
I co-reviewed this manuscript with one of the reviewers who provided the listed reports.

Referee #5

(Remarks to the Author)
The author has made substantial revisions and additions to the manuscript, addressing most concerns. However, several issues still require clarification, especially regarding modeling methods:

1. Model Performance: The authors still do not directly provide key evaluation metrics for RF modeling. Given that r in Fig. S3 is 0.66, R^2 should be around 0.44. According to Fig. S2, the R^2 value at higher time resolutions is lower, and should be only around 0.28, which is not a particularly favorable indicator for machine learning. Furthermore, while the authors currently use leave-one-out cross-validation to assess the performance of the RF method at spatial sites, have they considered the generalization capability and performance evaluation of the RF model on time series data?

2. What is the time series range used for RF model training—2012 to 2021? What time resolution did the authors employ? Some datasets may have lower time resolution; how did the authors handle differences in time resolution across datasets?

3. I still believe the authors should reconsider the manuscript's innovative points and highlights—should the focus lean more toward methodology or cognition? Integrating models of well-defined physical processes to enhance the interpretability of machine learning is a current hot research direction. However, the current exploration of this methodology clearly lacks sufficient depth, which is an aspect I find particularly intriguing. Additionally, have the authors compared their work to existing studies? Modeling and prediction of airborne particulate matter are now well-established fields. Does this manuscript's model outperform current approaches in capturing dust pollution events?

4. Certain intuitive verifications remain necessary. For example, in Fig. S7, is there any spatial correlation analysis based on satellite data or observational and research findings?

Version 2:

Reviewer comments:

Referee #5

(Remarks to the Author)
The authors have adequately responded to my suggestions and questions with clarifications and further data. I have no further feedback.

Open Access This Peer Review File is licensed under a Creative Commons Attribution 4.0 International License, which permits use, sharing, adaptation, distribution and reproduction in any medium or format, as long as you give appropriate credit to the original author(s) and the source, provide a link to the Creative Commons license, and indicate if changes were made.
In cases where reviewers are anonymous, credit should be given to 'Anonymous Referee' and the source.
The images or other third party material in this Peer Review File are included in the article's Creative Commons license, unless indicated otherwise in a credit line to the material. If material is not included in the article's Creative Commons license and your intended use is not permitted by statutory regulation or exceeds the permitted use, you will need to obtain permission directly from the copyright holder.
To view a copy of this license, visit https://creativecommons.org/licenses/by/4.0/

<u>Referee #1 (Remarks to the Author):</u>

This manuscript aims at both quantifying the short-term (2012-2021) variability of dust concentrations over Europe and also the long-term trends over the period from 1750 to present. The novelty consists of the aggregation of different sources of information that are: atmospheric mineral data from 103 rutal and urban sites and 18500 daily measurements in total, machine learning together with ice core records and dust long-term trends in European dust concentrations. The authors also discuss the climatic drivers associated with this variability.

I find this work is original and significant.

It helps quantifying the PM2.5 and PM10 baseline caused by dust over Europe (one of the priorities when assessing particulate concentrations over the region) and the methods used are convincing.

We thank the reviewer for their favorable review – we hope they will find the updated manuscript significantly improved.

The conclusions drawn from this study are reliable with the following caveat. With regards to the long term trends in Ca2+ estimated to have rise by 110% from the preindustrail (1750-1850) to the past decade (2010-2020), the authors should word it carefully as it reflects the measurements of 3 cores from a single geographical location in the Alps which is Colle Gnifetti. I regard this trend as credible since analysis at more sites published by Kok et al. 2023 (reference 5 in this manuscript) published in NEE, also discuss a similar albeit less intense long-term trends over the XXth century.

This is an insightful comment and we agree that the statement should be worded with greater caution given the limited number of ice-core sites. We have revised the text accordingly (lines 338–340), which now reads:

The trend derived from the three Colle Gnifetti cores is consistent with the multisite analysis of Kok et al. 2023[5], who report a similar but less pronounced increase over the 20th century.

I have several minor comments that could help still improve the manuscript that is already in good form. In particular, good care was put in having a good description in the Methods.

Minor points:

- It would be useful to give the correlation coefficients associated with Fig. S2a and S2b. This would help you make the point that the Random Forest (RF) algorithm predicts the dust fields more precisely than a state-of-the-art model such as the DREAM model.

The correlation coefficients are now shown on the plots, along with the fractional bias and RMSE.

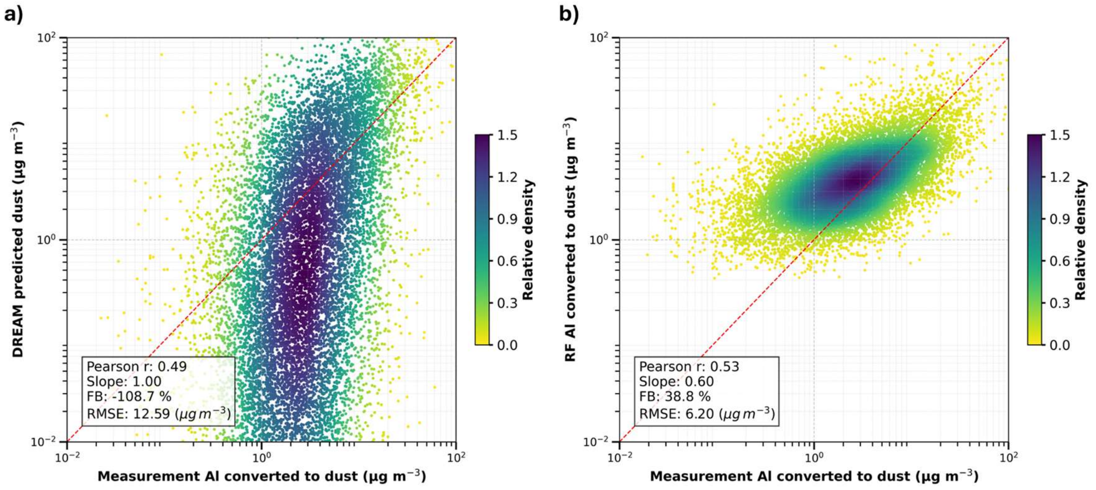

Fig S2 | Comparison between measured dust vs. (a) DREAM predicted dust, and (b) Random Forest (RF) predicted dust (b), along with relevant statistical metrics (Pearson correlation coefficient r, linear regression slope, fractional bias FB & root mean square error RMSE). Results are shown in a logarithmic scale and the color represents the relative density of measurements

- Fig. captions S6 and S7 need to be more precise and give an exact description of what is shown.

We thank the reviewer for the suggestion. The caption of Figure S6 (now Figure 7) has been revised for greater clarity and precision, while Figure S7 has been removed due to not being referenced in the main text. It now read as follows:

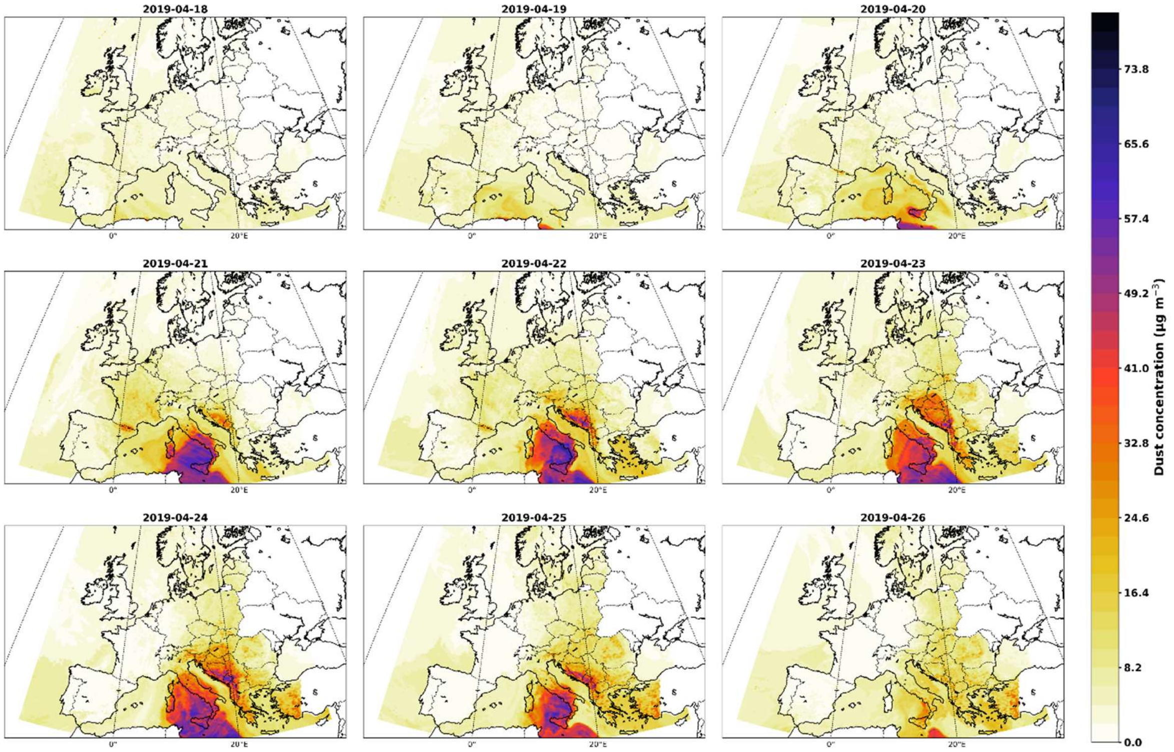

Fig S7 | Random forest–predicted dust concentrations (μg m^{-3}) showing the simulated transport trajectory of Saharan dust during the extreme event affecting the Balkans, as reported by Peshev et al. (2021), for the period 18 April 2019 (one day before the episode's onset) to 26 April 2019 (episode end).

- I understood that Figs. S8 and S9 are the results of the RF algorithm, it should be clearly stated in the Figure caption of these two figures.

We thank the reviewer for this helpful suggestion. The captions for Figures S8 and S9 have been revised to explicitly indicate that they present results from the random forest algorithm. They now read as follows:

Figure S8. Seasonal variation of random forest–predicted dust concentrations (μg m^{-3}), shown as 10-year monthly averages for the study domain.

Figure S9. Seasonal variation of random forest–predicted dust concentrations, expressed as monthly averages relative to the yearly maximum monthly concentration of the respective grid cell for the study domain.

- The figure caption for Fig. S7 is incomplete. Please write full sentences to describe what is depiected in this figure

We thank the reviewer for this careful reading. On checking the Supplementary Information we found that the figure in question is not referenced in the main text; to avoid confusion we have removed Fig. S7 from the updated Supplementary Information. The SI has been renumbered accordingly, and all cross-references have been checked and updated.

- Showing 2 decimals for the mean concentration of dust (ug m-3) is a bit provocative. You have an incredible confidence in the RF alrorithm if you trust it to the second decimal. Already having one decimal would be optimistic, I would not show a second decimal.

We agree that reporting to the second decimal overstates the precision of the random forest predictions. Accordingly, all relevant plots have been updated to show only one decimal.

- In all the occurrences of the following unit: ug m-3 year-1 you have a space missing between m-3 and year-1 please correct all such occurrences

All instances across the text have been corrected.

- Please replace 'yearly change in concentration during background' with 'yearly concentration change for background conditions'

We replaced all instances.

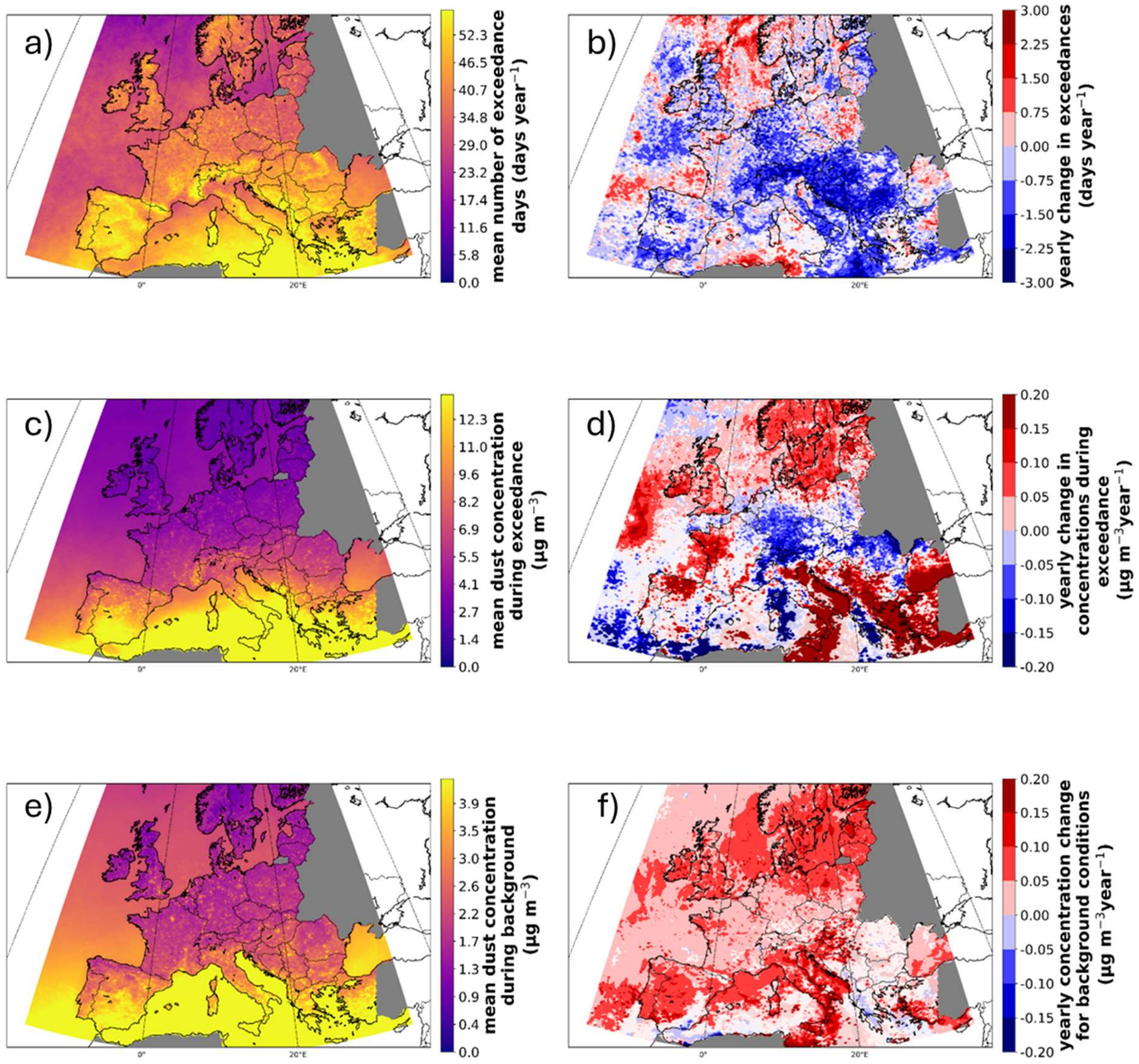

Fig 4 | Dust episodes and their trends in Europe during the period 2012-2021: mean number of days where a dust exceedance occurs (a) and trend (b), mean dust exceedance concentrations (c) and trend (d) & mean dust concentrations during background (e) and trend (f). Concentrations are reported for PM_{10} in μg m^{-3} and their corresponding trends in μg m^{-3} year^{-1}, while the number of exceedance days is reported as days with their corresponding trend reported as days year^{-1}. A trend is defined as the mean of a 100 times bootstrapped linear regression for the variable of interest, and shown on the map if there is no sign change between the 25th and 75th percentile of the bootstrap results. Areas where a sign change occurs are denoted with white (for regional means considered zero), while areas where no land use data exists in gray.

- Figure S10: Avoid acronyms in Figure captions, a figure caption should be understood without reading the main text, hence write 'Palmer Drought Severity Index' instead of 'PDSI'.

We now spell the full name of NAO and PDSI in the updated caption of Figure S10 (now Figure S11).

Fig S11 | Spatiotemporal correlation between December-March averaged North Atlantic Oscillation (NAO) and annual Palmer Drought Severity Index (PDSI) between 1901 and 2021.

- Same applies for Figure S4, RF can stand for many things and is generally used for Radiative Forcing when considering aerosols, here you to refer to Random Forest Algorithm.

We thank the reviewer for this helpful comment. The abbreviation "RF" has now been replaced with "Random Forest" in Figure S4 and all other SI figures to avoid any ambiguity. All updated instances are found below.

Fig S2 | Comparison between measured dust vs. (a) DREAM predicted dust, and (b) Random Forest (RF) predicted dust (b), along with relevant statistical metrics (Pearson correlation coefficient r, linear regression slope, fractional bias FB & root mean square error RMSE). Results are shown in a logarithmic scale and the color represents the relative density of measurements.

Fig S4 | SHAPLEY plot for the top 10 in importance model variables (a), and Random Forest (RF) predicted variable weights (b).

Fig S5 | Timeseries for the sites with largest number of daily measurements in the dataset, for the measurement derived dust (solid line) and the Random Forest (RF) model derived dust (dashed pink line).

Fig S7 | Random forest–predicted dust concentrations (μg m^{-3}) showing the simulated transport trajectory of Saharan dust during the extreme event affecting the Balkans, as reported by Peshev et al. (2021), for the period 18 April 2019 (one day before the episode's onset) to 26 April 2019 (episode end).

Fig S8 | Seasonal variation of random forest–predicted dust concentrations (μg m^{-3}), shown as 10-year monthly averages over the study domain.

Fig S9 | Seasonal variation of random forest–predicted dust concentrations, expressed as normalized monthly averages relative to the yearly maximum over the study domain.

- Line 378-379 you could indicate to the reader that you are giving the set of parameters used to optimize the Random Forest Algorithm in the lines below.

We thank the reviewer for this suggestion. We have clarified the text to guide the reader, and the parameters are now explicitly listed in lines 517–518:

"(parameters used in this work are n_estimators=150, max_depth=None, criterion='friedman_mse', bootstrap=True, random_state=66)."

<u>Referee #2 (Remarks to the Author):</u>

In this continental scale study, the authors developed a random forest model using measured dust meta ratios to estimate daily dust PM10 concentrations in Europe from 2012 to 2021. The authors then reported the temporal trend and spatial characteristics of transported dust events, their impact on regional air quality, and the associated mortality burden. As dust transported from Africa pose an increasing threat to air quality and public health in Europe, this study is timely and policy relevant. While the results presented here may appeal to readers interested in understanding the health impact of environmental risk factors driven by climate change such as transported dust, there are major technical concerns regarding the dust PM10 model development and performance. These validity of the technical approach as well as the quality of the predicted dust PM10 concentration put the robustness of the study findings in doubt. This manuscript in its current form is not suitable for publication. Specific comments are provided below.

We thank the reviewer for their detailed and constructive feedback. We have carefully considered the concerns raised regarding the model development and performance. Based on the reviewer's comments, we conducted additional analyses to further evaluate model performance, validate our approach, and increase the robustness of the conclusions. All relevant sections have been updated accordingly considering all the reviewer's comments and suggestions, to clarify the technical methodology and provide stronger evidence supporting the reliability of the predicted dust PM10 concentrations.

Major comments:

• Elemental ratios for dust PM10 concentration reconstruction. Dust PM10 concentrations in this study, as in many other previous studies, are reconstructed using element concentrations and elemental ratios. Specifically, Al was selected as the key tracer for transported dust and zero intercept linear regression models were built to estimate the Si:Al, Ti:Al, Ca:Al, and Fe:Al ratios. This step is crucial to the values and quality of the estimated dust PM10 concentrations. There are multiple concerns here. First, the authors state that Al and Ti are ideal tracers for transported dust detection. However, the article that they cite is from 2001. There are more current citations regarding this, including some that indicate the use of multiple tracers (Al, Fe, Th, Ti) allows for more accurate estimates of dust and less uncertainty. Second, authors state that these elemental ratios align well with dust transport and Saharah source estimates from Chiapello et al. from 1997. It is likely that there are more recent reports of these values and authors should provide additional information regarding if and or how the ratios from the 1997 article are still acceptable to use.

We thank the reviewer for raising these important points.

Regarding the choice of tracer, we acknowledge that a multi-tracer approach could in principle reduce uncertainty. However, such an approach would substantially limit the usable dataset, as most monitoring sites do not report all suggested tracers making it impossible to train the random forest model consistently across regions. Silicon (Si) would be an ideal single tracer due to its strong association with transported dust, but Si measurements are less abundant and are often affected by artifacts from quartz fiber filters. In locations where both Al and Si are measured, they are highly correlated, supporting the use of Al as a reliable and practical transported dust proxy. We also note that several of the additional tracers proposed in recent literature (e.g., Th) are associated with high analytical uncertainties and are not available. Since the success of the model depends on its ability to learn from a broad and coherent observational dataset, it is crucial to rely on widely available and consistently measured elements. We now clarify this in the main text (lines 185–190):

For quantifying dust, we opted for a single-tracer approach rather than a multi-tracer one, as most locations lack comprehensive tracer data, preventing consistent training of the random forest model. While Si would be an ideal tracer due to its clear association with transported dust, Si measurements are notably less abundant than Al due to common sampling methodologies using quartz filters. Given the strong correlation between Al and Si where both are available, we rely on Al as a dust proxy in the RF model (Fig. E1).

Regarding elemental ratios, we conducted an updated literature review to contextualize our results. Few studies report elemental ratios for dust arriving in Europe, with notable datasets including Marconi et al. (2014) from Lampedusa and Loskot et al. (2024) from the Czech Republic. Their ratios are consistent with ours within the reported uncertainty ranges. Furthermore, our ratios are within the variability of North Africa source estimates (Liu et al., 2022). These comparisons are

now shown in Fig. 1b, along with their corresponding citations, and the text has been updated in lines 143-147 to read:

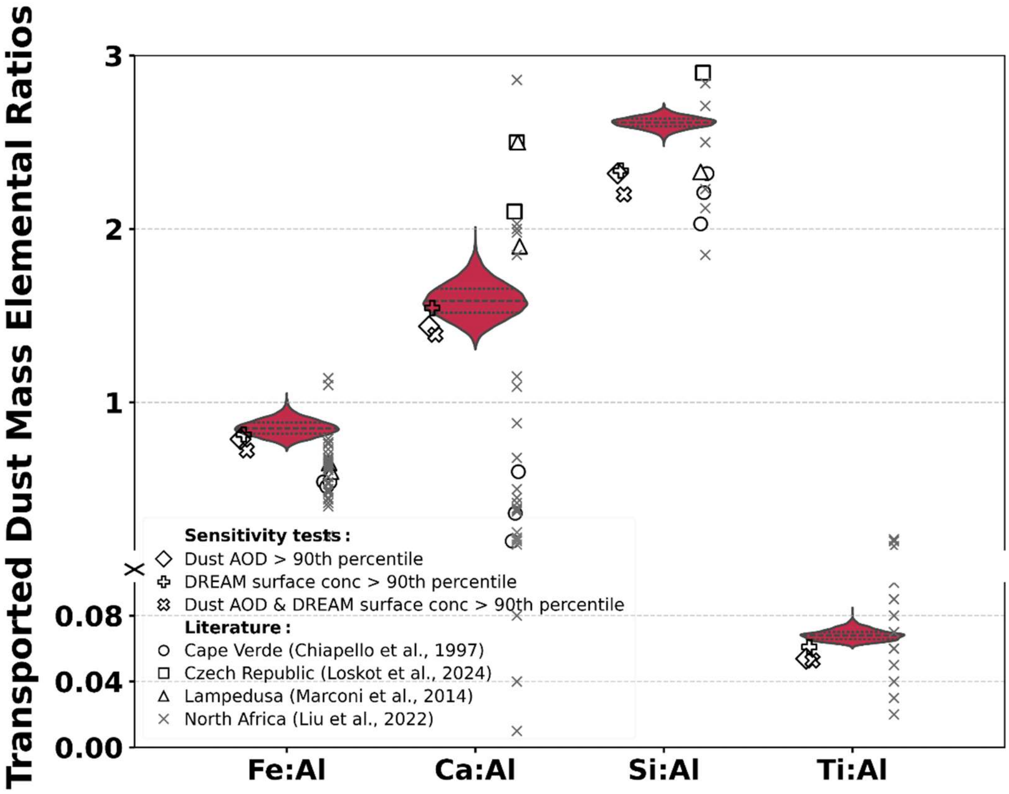

Fig 1 | Dust observations and elemental ratios of transported dust in Europe. a) average aluminium concentrations for 103 locations in Europe. The size of the dot corresponds to the number of daily measurements, while the color to the average concentration of *Al* at this location. High *Al* concentrations show the large impact of transported dust on air quality in Southern Europe. b) Violin plots of elemental ratios of transported dust, estimated via bootstrapped zero-intercept linear regressions applied to daily data with elevated transported dust concentrations (aluminium concentrations >1 $\mu g\,m^{-3}$ for Fe:Al and Ca:Al). The diamonds, crosses and x's correspond to filtering to the 90th percentile of dust optical depth, DREAM dust surface concentration and both. Circles, squares and triangles represent literature values from Chiapello et al 1997[46], Loskot et al. 2024[48], Marconi et al. 2014[47] for dust arriving to Europe, while North Africa source estimates from Liu et al., 2022[44] are denoted with the gray x's.

These elemental ratios align well with reported values for dust transported to Europe[1-5] and North Africa source estimates[3], acknowledging variability in elemental composition depending on the emission area, particularly for Calcium. Despite this variability, given the extensive spatial and temporal coverage of our dataset across urban and rural environments,

these ratios provide the most robust characterization of the average elemental composition of transported dust in Europe to date[6].

Put these concerns aside, three technical issues emerge that put the validity of the simplified approach adopted in this study in doubt.

a. First, as shown in Fig 1b, each of the elemental ratios have a non-trivial scatter around the mean values. This uncertainty has major impacts on the estimated dust PM10 concentrations (line 160 - 164) but was not mentioned in any of the following analyses. While the authors state that their approach aligned well with some previously published material, it is important to include how different these ratios are in comparison and whether utilizing these ratios introduces any bias or uncertainty into the estimates of dust concentration.

We thank the reviewer for pointing this out. We quantified the impact of uncertainty in the average elemental ratios—defined here as one standard deviation from the bootstrap results shown in Fig. 1b (Si:Al 2.61±0.033, Ti:Al , 0.068±0.003, , Ca:Al 1.58±0.099, and , Fe:Al 0.85±0.052)—on the dust estimates using uncertainty propagation based on the formula: 2.2Al + 2.49Si + 1.63Ca + 1.94Ti + 2.42Fe. This results in a total dust estimate of (13.5 ± 0.377) × Al. Notably, the largest contributions to the overall uncertainty stem from Fe and Ca, which are also influenced by local sources.

Information regarding the uncertainties in elemental ratios has been added to a new section in the Methods section, titled "Uncertainty of elemental ratios and dust estimates", lines 448-475. Errors have been also added on all numbers reported in the text.

Uncertainty of elemental ratios and impact on dust estimate

We quantified the impact of uncertainty in the elemental ratios—defined here as one standard deviation from the bootstrap results shown in Fig. 1b (Si:Al 2.61±0.033, Ti:Al , 0.068±0.003, , Ca:Al 1.58±0.099, and , Fe:Al 0.89±0.052)—on the dust estimates using uncertainty propagation based on the formula: 2.2Al + 2.49Si + 1.63Ca + 1.94Ti + 2.42Fe. This results in a total dust estimate of (13.5 ± 0.377) × Al. Notably, the largest contributions to the overall uncertainty stem from Fe and Ca, which are also influenced by substantial local sources. Zero-intercept fits were used for element–element plots (e.g., Ca concentration vs. Al concentration) to estimate dust-phase mass-based elemental ratios under the assumption that—after blank correction and removal of non-dust contributions—both analytes originate from the same dust endmember. In that case, when the dust contribution vanishes (Al → 0), the co-emitted element should also vanish (Ca → 0), implying a physically meaningful intercept of zero. In addition, we tested a linear model with an intercept using bootstrap analysis and found that zero lies within one standard deviation of the mean intercept (intercept ± σ_intercept), supporting the use of a zero-intercept model. Given this result, along with the physical consistency and practical advantages of a zero-intercept regression—such as enabling the use of average ratios to

estimate other components—we opted for the zero-intercept model. A summary table of the relevant statistics is provided in the Supplement (Table S2).

Elemental ratios on days with low amounts of transported dust can be substantially affected by local sources. With a rather high Aluminium concentration threshold of >1 ug/m3 for determining the transported dust elemental ratios, we aimed at minimizing the impact of local sources on the elemental ratios. In addition, we performed a sensitivity analysis varying the threshold showing that the elemental ratios vary with cutoffs above 0.5 $\mu g\ m^{-3}$ for Ca:Al and 0.75 $\mu g\ m^{-3}$ for Fe:Al within the 1 standard deviation of the value obtained with a cutoff of 1 ug/m3. Further, we investigated model-driven criteria for dust episodes instead of using a static Aluminium concentration cutoff using the DREAM and optical depth values and only including data points that were higher than the 90th percentile in those (each alone or both at the same time), as a proxy for dust events. This approach resulted in similar values (Fig. 1b & S13).

Additionally, as mentioned in our previous response, we performed an updated literature with the comparisons shown in the updated Fig. 1b

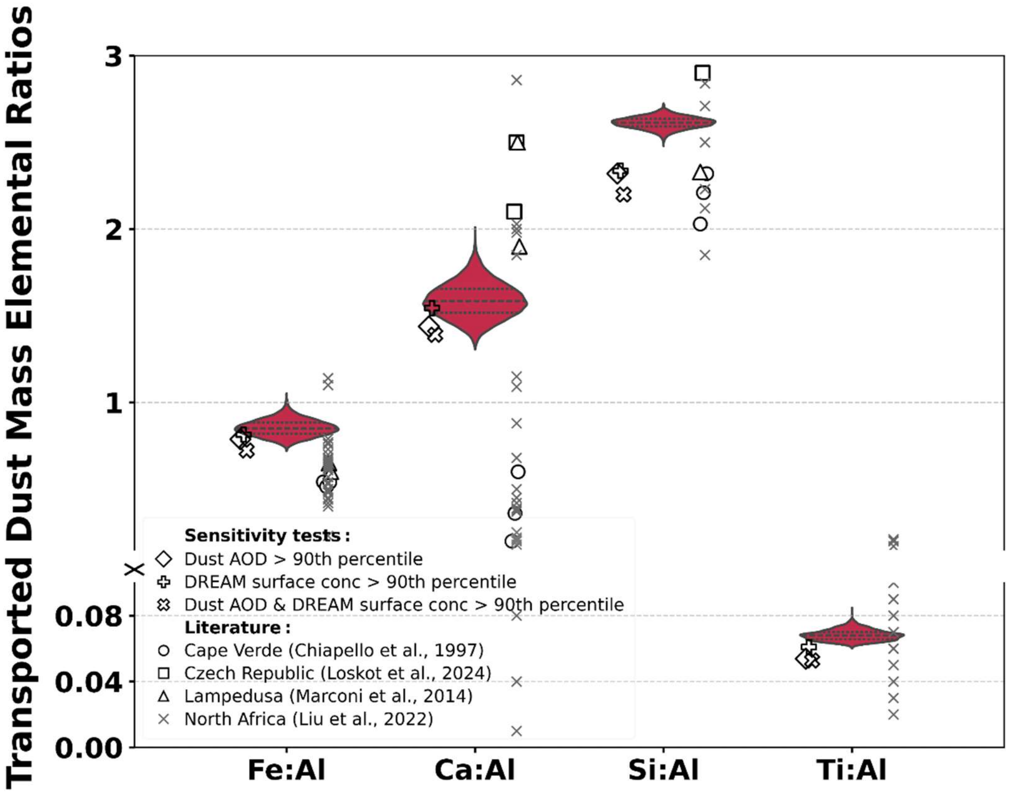

Fig 2 | Dust observations and elemental ratios of transported dust in Europe. a) average aluminium concentrations for 103 locations in Europe. The size of the dot corresponds to the number of daily measurements, while the color to the average concentration of *Al* at this location. High *Al* concentrations show the large impact of transported dust on air quality in Southern Europe. b) Violin plots of elemental ratios of

transported dust, estimated via bootstrapped zero-intercept linear regressions applied to daily data with elevated transported dust concentrations (aluminium concentrations >1 μg m^{-3} for Fe:Al and Ca:Al). The diamonds, crosses and x's correspond to filtering to the 90th percentile of dust optical depth, DREAM dust surface concentration and both. Circles, squares and triangles represent literature values from Chiapello et al 1997[46], Loskot et al. 2024[48], Marconi et al. 2014[47] for dust arriving to Europe, while North Africa source estimates from Liu et al., 2022[44] are denoted with the gray x's.

b. Second, the authors provided no justification for the choice of zero intercept regression. It has the advantage of giving a neat elemental ratio, but usually the fit is worse than a standard simple linear regression with both intercept and slope. This tradeoff needs to be discussed in the paper.

We thank the reviewer for raising this point. We used zero-intercept fits for element–element plots to estimate mass-based elemental ratios under the assumption that—after blank correction—both analytes originate from the same dust endmember. In that case, when the dust contribution vanishes (e.g. Al → 0), the co-emitted element should also vanish (e.g. Ti → 0), implying a physically meaningful intercept of zero. In addition, we tested a linear model with an intercept using bootstrap analysis and found that zero lies within one standard deviation of the mean intercept (intercept ± σ_intercept), supporting the use of a zero-intercept model. Given this result, along with the physical consistency and practical advantages of a zero-intercept regression—such as enabling the use of average ratios to estimate other components—we opted for the zero-intercept model. A summary table of the relevant statistics is provided in the Supplement and the justification for the choice is; the Methods section "Uncertainty of elemental ratios and impact on dust estimates" (posted in our response to the previous comment), includes this information.

Table S2 | Slopes and intercept (with corresponding uncertainties, defined as one standard deviation for a 10000-round bootstrap) for the comparison of the zero intercept (forced origin) and intercept models.

specie	forced origin slope	linear regression slope	linear regression intercept
**Fe**	0.85±0.052	0.81±0.14	0.11±0.23
**Ca**	1.58±0.099	1.46±0.29	0.29±0.47
**Si**	2.61±0.033	2.34±0.16	0.09±0.25
**Ti**	0.068±0.003	0.069±0.013	-0.016±0.022

c. Finally, the regression models were developed only for high Al concentrations (line 119). What is the rational behind this choice of 1μg/m3? How would the estimated ratios change with this selected threshold? This crucial sensitivity analysis is missing. In addition, the elemental ratio conversion yields dust estimate of 12.3 times the Al concentration (line

163). This translates into a dust PM10 concentration of above 12.3 µg/m3 when the elemental ratios were estimated. According to Fig 4c, this level is near the maximum mean dust concentration across the entire study domain during the study period. In other words, the elemental ratio conversion does not apply to most dust episodes. The choice of this selected Al concentration threshold of 1µg/m3 doesn't appear to be appropriate.

This is an important point; we clarify that the 1 µg m^{-3} Al cutoff is only used to determine the elemental ratios for dust reconstruction; it is NOT used to detect dust episodes, which are identified by comparing daily values to background concentrations. Elemental ratios on days with low dust amounts are influenced by local sources, and applying the >1 µg m^{-3} threshold helps minimize this influence when estimating the transported dust elemental ratios.

To address the reviewer's concern, we performed a sensitivity analysis by varying the Al threshold. The results show that the estimated elemental ratios remain within one standard deviation of the values obtained using the 1 µg m^{-3} cutoff for Ca:Al (thresholds >0.5 µg m^{-3}) and Fe:Al (thresholds >0.75 µg m^{-3}). This demonstrates that our results are robust to reasonable changes in the cutoff.

Additionally, we tested a model-driven criterion for defining dust episodes using DREAM model output (dust surface concentration) and satellite reanalysis dust optical depth, selecting only days above the 90th percentile for these indicators. This approach produced similar elemental ratios, further supporting the robustness of our method. The results of this analysis are included in an updated Figure 1 with a reference in the text.

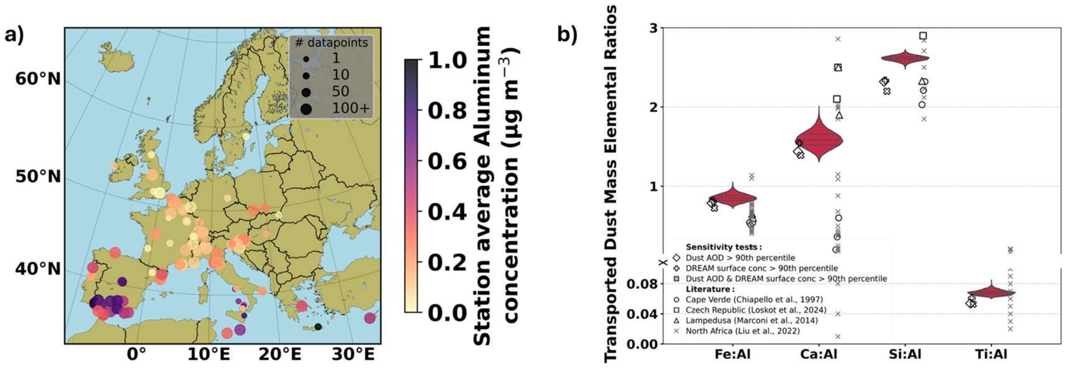

Fig 3 | Dust observations and elemental ratios of transported dust in Europe. a) average aluminium concentrations for 103 locations in Europe. The size of the dot corresponds to the number of daily measurements, while the color to the average concentration of *Al* at this location. High *Al* concentrations show the large impact of transported dust on air quality in Southern Europe. b) Violin plots of elemental ratios of transported dust, estimated via bootstrapped zero-intercept linear regressions applied to daily data with elevated transported dust concentrations (aluminium concentrations >1 µg m^{-3} for Fe:Al and Ca:Al). The diamonds, crosses and x's correspond to filtering to the 90th percentile of dust optical depth, DREAM dust surface concentration and both. Circles, squares and triangles represent literature values from Chiapello et al 1997[46],

Loskot et al. 2024[48], Marconi et al. 2014[47] for dust arriving to Europe, while North Africa source estimates from Liu et al., 2022[44] are denoted with the gray x's.

To provide full transparency, we have added a new subsection in the Methods, "Uncertainty of elemental ratios and impact on dust estimates", and corresponding Supplementary Information plot (S13) to describe the sensitivity analyses and quantify uncertainty associated with the Al threshold.

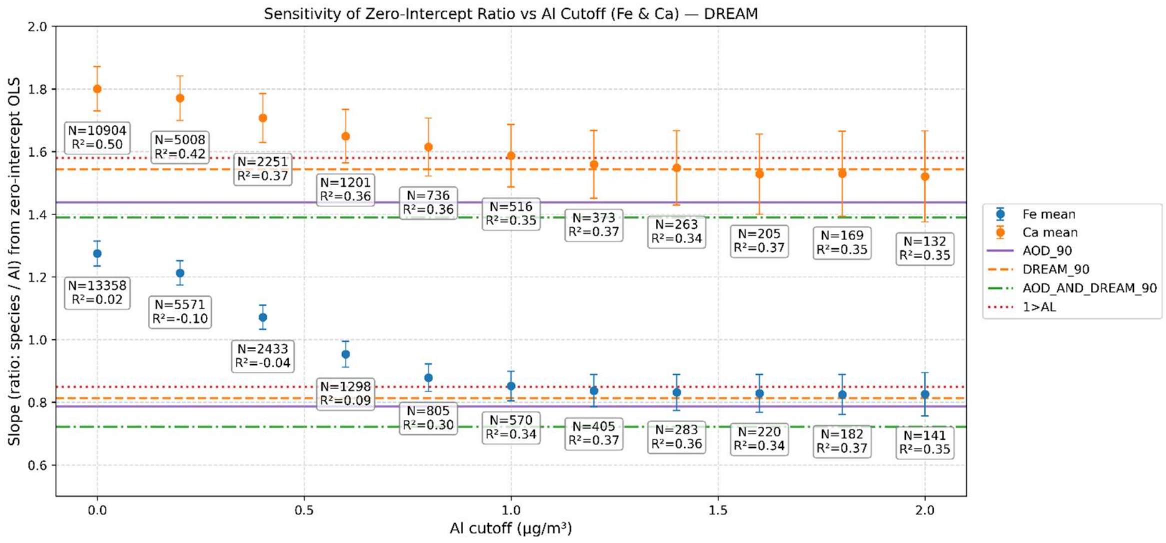

Fig S13 | Sensitivity of the mass-based elemental ratios of Fe/Al & Ca/Al for different Al cutoffs. The ratios for the cases where Al>1μg m^{-3}, and for the 90th percentile of DREAM surface dust concentration, dust AOD and both at the same time are shown as horizontal lines.

2. Random forest model for dust PM10 concentration prediction. A random forest model was trained to predict Al concentrations in the entire study domain in order to estimate dust PM10 concentrations. Authors used land use variables to inform the model, but there is little discussion regarding previously published works that point to these specific variables as important considerations to include in the model. Also, there is little discussion regarding as to whether variables like soil erodibility, soil moisture, and terrain roughness were considered and whether any seasonal vegetation changes were accounted for. There are also technical concerns related to model performance.

We thank the reviewer for raising these points. We would like to clarify that the dominant predictors in the random forest model are the physical dust modeling outputs (DREAM), reanalysis dust AOD, and meteorological variables, whereas local land-use variables play a comparatively minor role (Figure S4). As already noted in the manuscript (lines 219–223), this indicates that most dust-related elements are dominated by long-range transport rather than local resuspension. Local land-use variables were nevertheless included to account for potential contributions from resuspended dust, which has been suggested to increase with urbanization and proximity to roads. However, our results show that, at the European scale, transported dust controls dust concentrations, with local variables exerting only a weak influence on model predictions.

We agree that the additionally suggested local variables (soil erodibility, soil moisture, terrain roughness) can be important for local dust emissions. To assess their impact, we used data from the European Soil Database (https://esdac.jrc.ec.europa.eu/content/european-soil-database-derived-data). Coverage was incomplete for some regions (e.g. the Balkans), which limited map generation, but data were available for all stations used in model training and evaluation. We found that including these variables did not improve model performance and had negligible importance in the RF model (<0.01%). Consequently, these variables were not included in the final analysis. A new figure has been added to the supplement to present this finding:

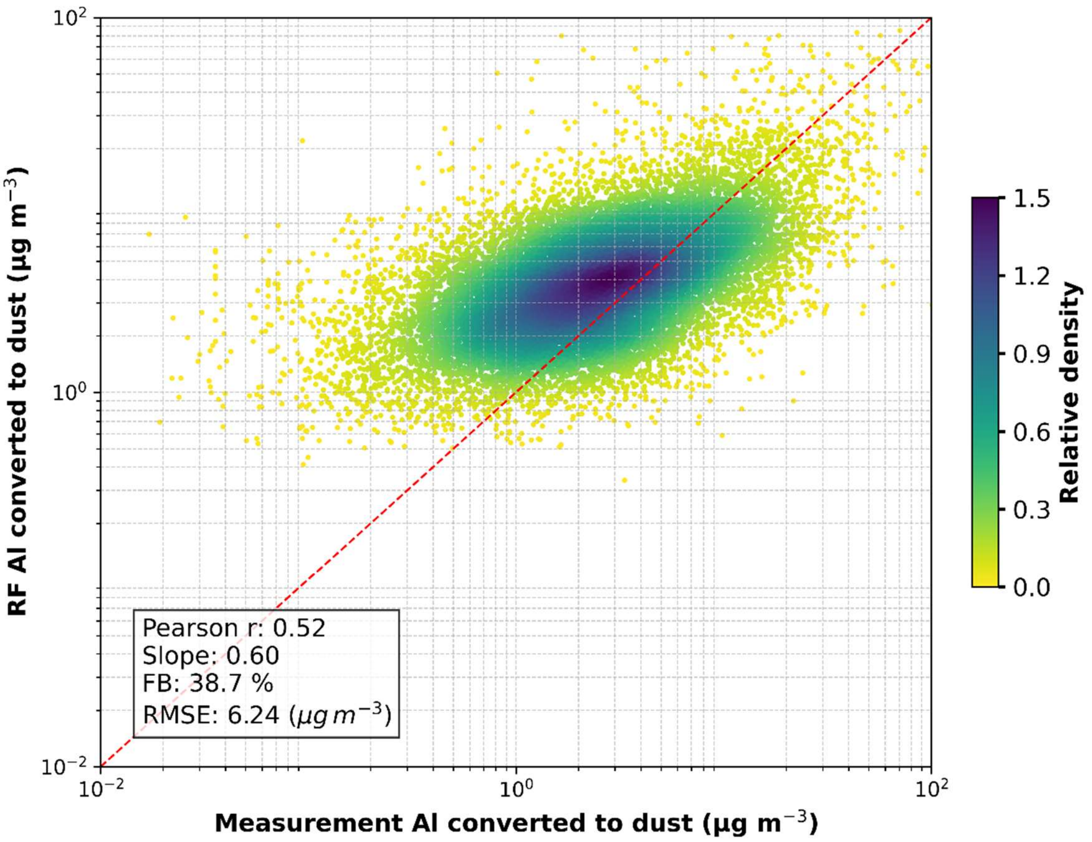

Fig S10 | Model training results when all available soil variables from the European Soil Database (https://esdac.jrc.ec.europa.eu/content/european-soil-database-v2-raster-library-1kmx1km, last accessed: 05.11.2025) are added, including soil moisture & erodibility. Also shown are relevant statistical metrics (Pearson correlation coefficient r, linear regression slope, fractional bias FB & root mean square error RMSE). Results are shown in a logarithmic scale and the color represents the relative density of measurements.

Vegetation is already accounted for in the model via the natural green land use variable; however, seasonal vegetation changes were not explicitly included. This clarification has been added to the Methods of the manuscript, under the Model inputs section (lines 531-538).

Finally, Coordination of information on the environment (CORINE) annual land use data at 200m resolution for entire Europe were also used as RF models inputs for the training data set (i.e. stations) including the fractions of 14 land use types[8] (e.g. urban fraction, natural green, agriculture, barren land), population density[9], altitude[10], and road length and category[11]. Vegetation is already accounted for in the model via the natural green land use variable; however, seasonal vegetation changes were not explicitly included. A comprehensive list of variables can be found at the supplementary information (Table S4).

a. As shown in Fig S2, the cross-validation results indicate that RF model overestimate converted dust concentration by a factor of 2 or more at low to medium levels (e.g., below 10 μg/m3). This bias becomes more pronounced at lower concentrations, suggesting that its ability to accurately characterize dust concentrations deteriorates with latitude. Without additional correction, its ability to detect subtle time trend is also questionable. For example, concentration increase for most of Europe was estimated at 0.05 μg/m3 per year (line 211). No test results of the statistical significance of this trend were shown.

The reviewer is correct in pointing out that there is a bias at lower daily concentrations. This, however, should not impact the model's ability to discern yearly trends. When aggregating the leave-1-out validation results on a yearly basis the bias is much less pronounced, as shown below and in the new Figure S3 in the updated version of the SI.

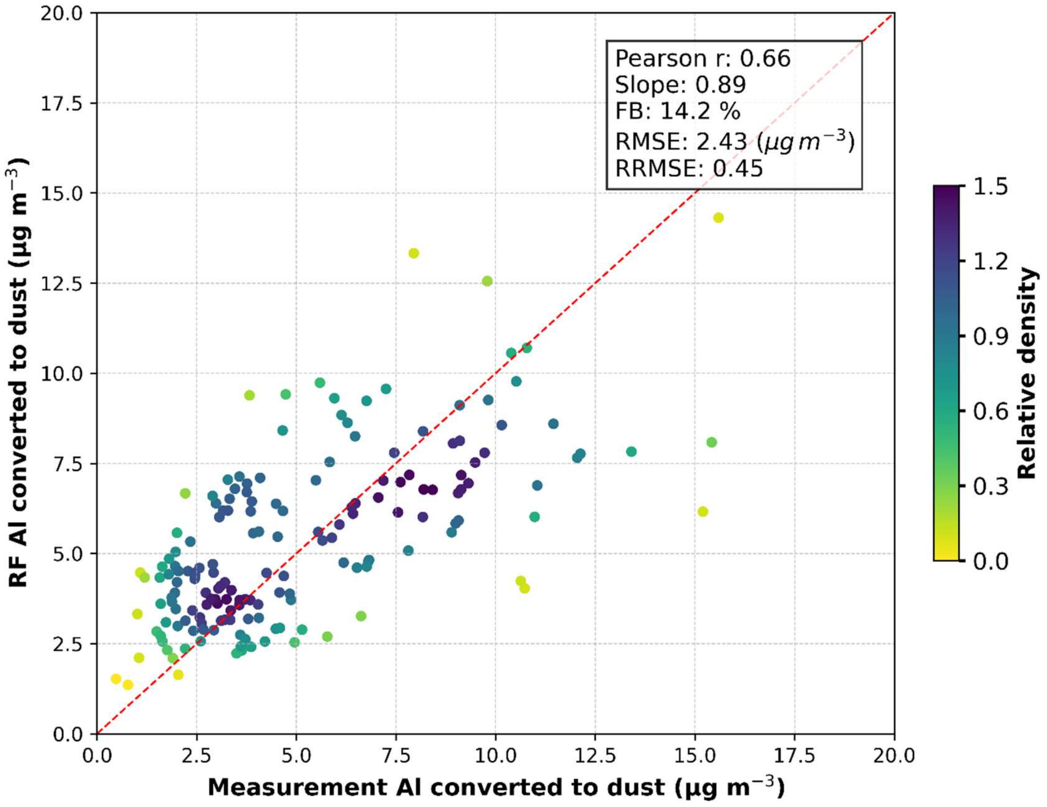

Fig S3 | Leave-1-out results aggregated on a yearly basis, with relevant statistical metrics (Pearson correlation coefficient r, linear regression slope, fractional bias FB, root mean square error RMSE and relative root mean square error RRMSE).

Similar behavior is seen for the sites with the longest timeseries in Fig. S6, where no time-dependent bias is observed in the model results, indicating that the model is capable of capturing yearly trends accurately.

While the robustness of the trends was not discussed in detail in the text, we validated the trends through bootstrapped linear regression, where we consider a gridcell's trend significant if there is no sign change between the 25th and 75th percentile of the bootstrap results (Figs 3, 4 captions). It has now been explicitly mentioned throughout the text in addition to the clarifications in the figure captions.

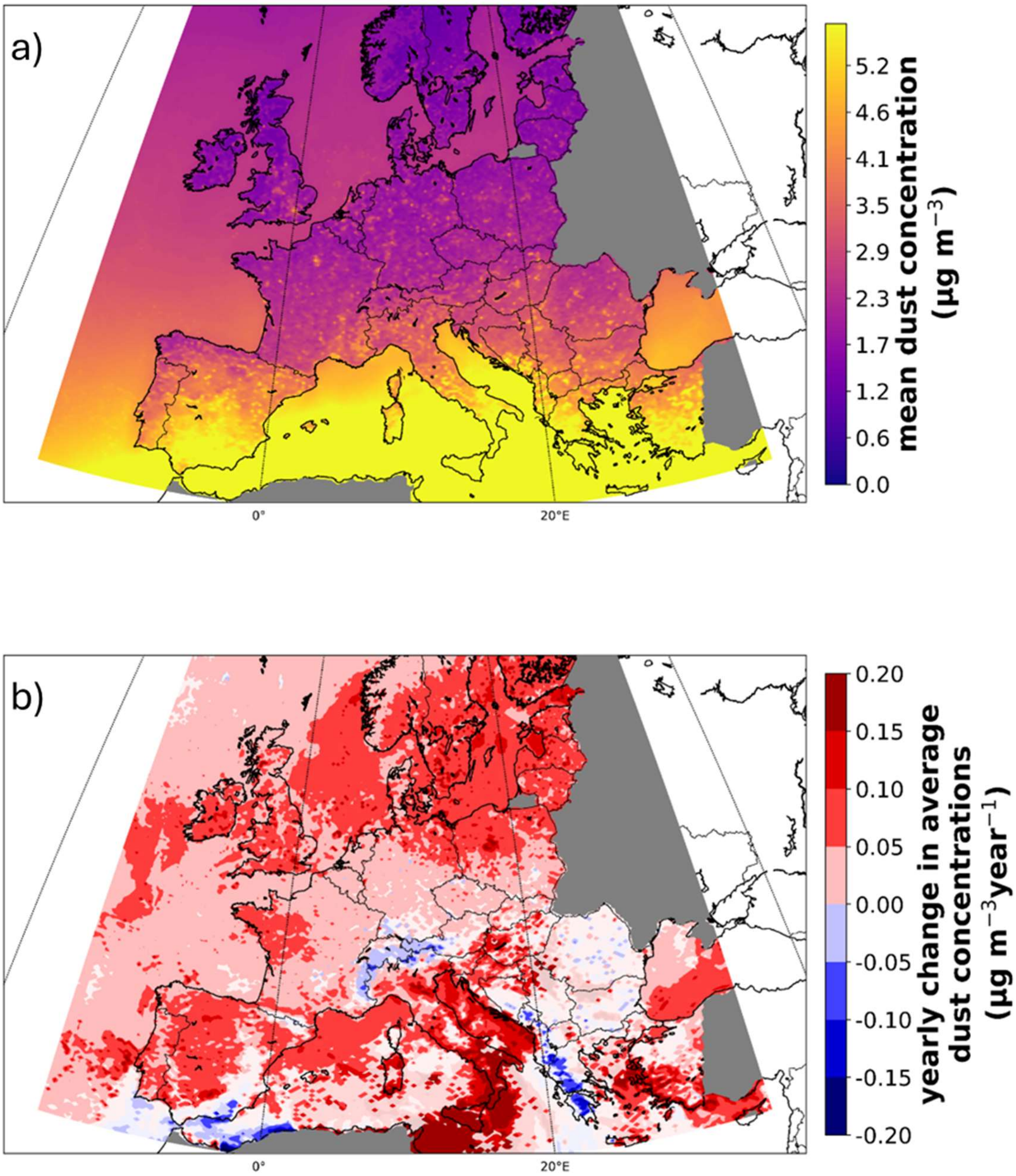

Fig 3 | Dust concentrations and their changes in Europe during the period 2012-2021: mean dust concentrations (a) and trend (b). Concentrations are reported in μg m^{-3} and their corresponding trends in μg m^{-3}year^{-1}. A trend is defined as the mean of a 100 times

bootstrapped linear regression for the variable of interest, and shown on the map if there is no sign change between the 25th and 75th percentile of the bootstrap results. Areas where a sign change occurs are denoted with white (for regional means considered zero), while areas where no land use data exists are denoted in gray.

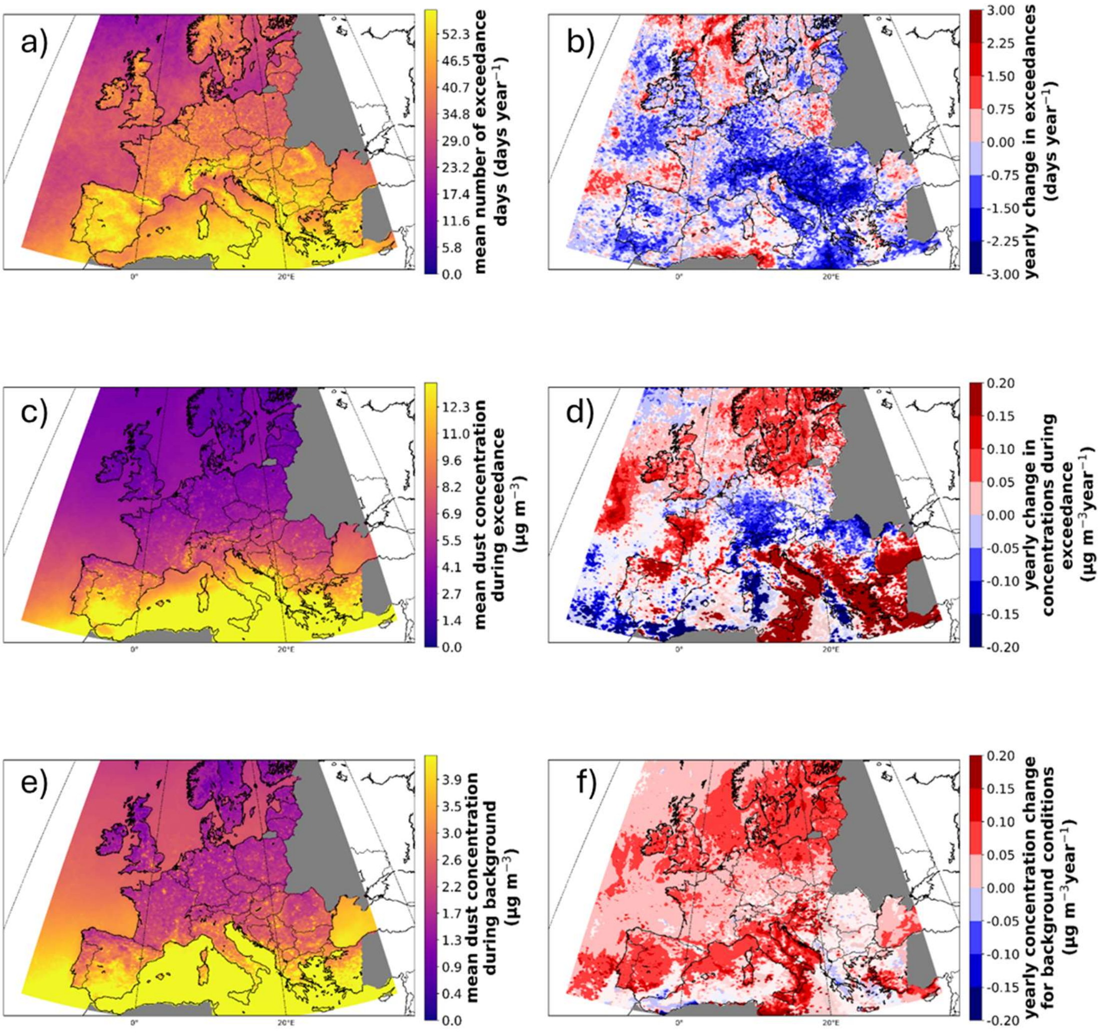

Fig 4 | Dust episodes and their trends in Europe during the period 2012-2021: mean number of days where a dust exceedance occurs (a) and trend (b), mean dust exceedance concentrations (c) and trend (d) & mean dust concentrations during background (e) and trend (f). Concentrations are reported for PM_{10} in µg m^{-3} and their corresponding trends in µg m^{-3}year^{-1}, while the number of exceedance days is reported as days with their corresponding trend reported as days year^{-1}. A trend is defined as the mean of a 100 times bootstrapped linear regression for the variable of interest, and shown on the map if there is no sign change between the 25th and 75th percentile of the bootstrap results. Areas where a sign change occurs are denoted with white (for regional means considered zero), while areas where no land use data exists in gray.

b. The authors point to Figure S4 as evidence that the model accurately depicts multi-year dust concentrations. While the figure illustrates the capture of the general trend, there seems to be substantial differences at times between the observed values and model estimates. There is no discussion regarding how this could impact the results or whether any correction or calibration was done.

The reviewer is correct in pointing out that there are sometimes differences between calculated and random forest based dust concentrations. However, these differences do not result into a temporally dependent bias as shown in Fig. S3 & S6. Therefore, potential differences in observed values and model estimates should not have an appreciable impact on the yearly trends that this manuscript discusses.

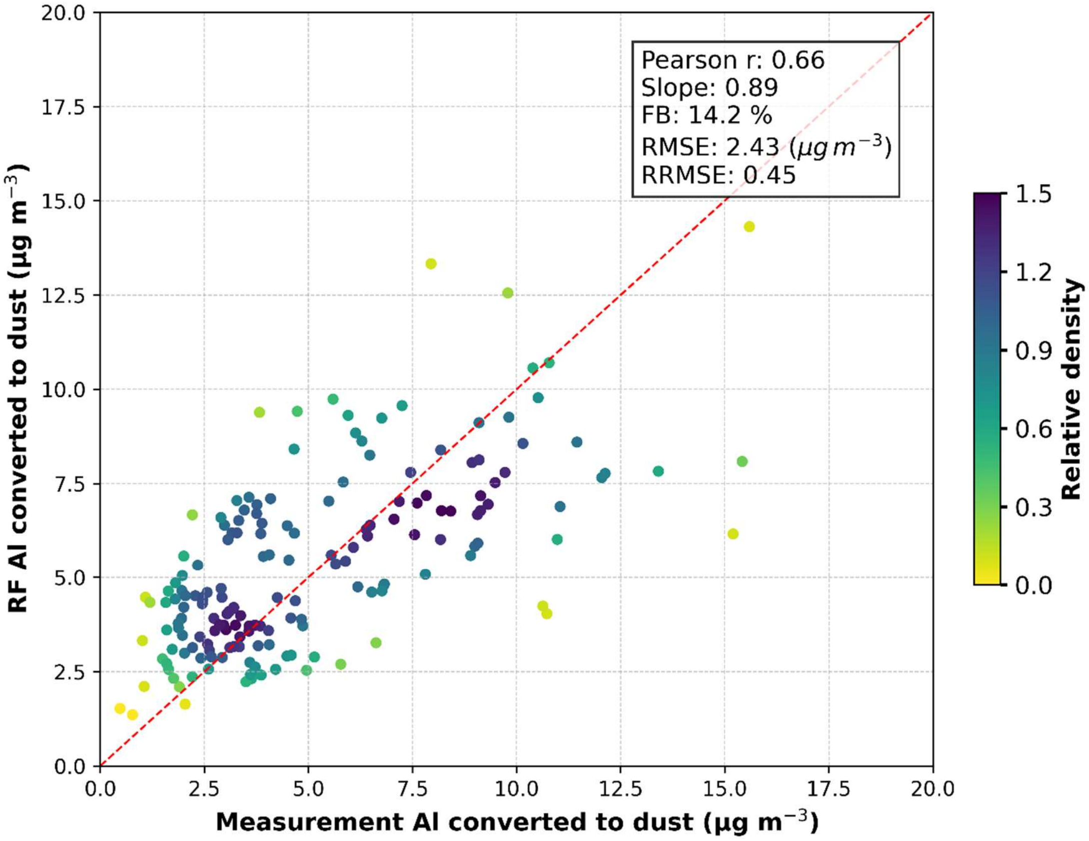

Fig S3 | Leave-1-out results aggregated on a yearly basis, with relevant statistical metrics (Pearson correlation coefficient r, linear regression slope, fractional bias FB, root mean square error RMSE and relative root mean square error RRMSE).

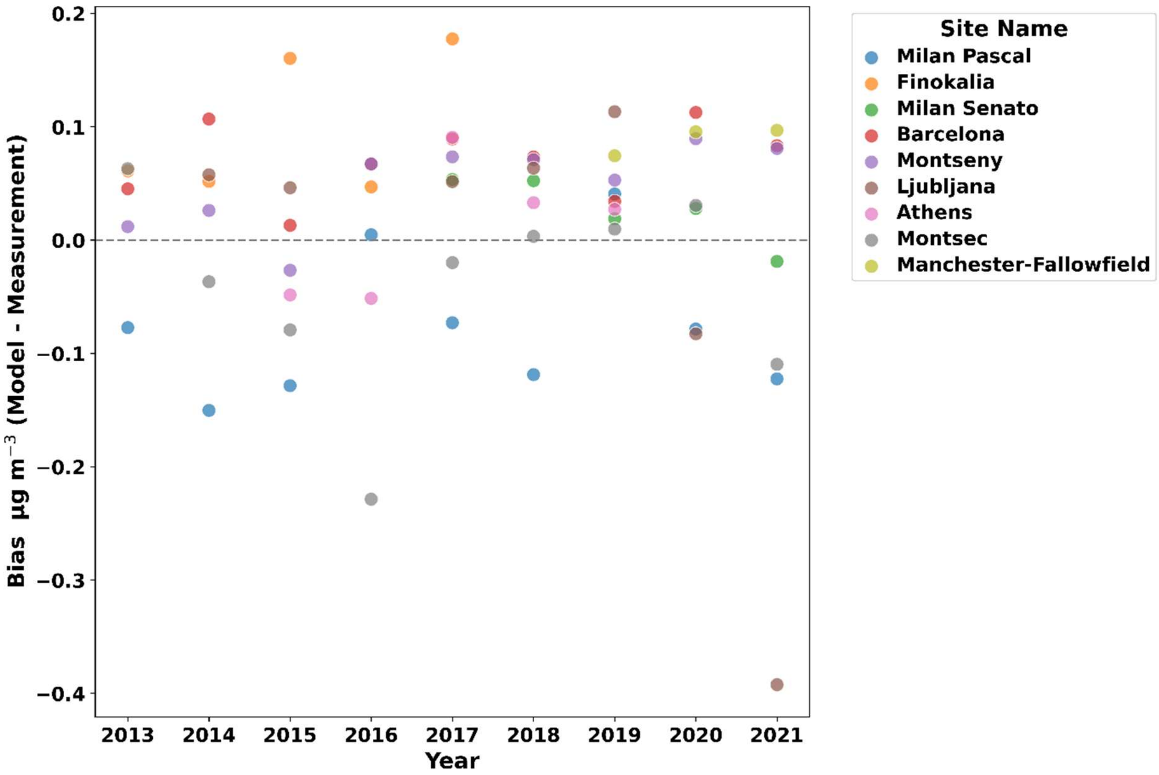

Fig S6 | Temporal bias (yearly model average value minus yearly measurement value) for the stations with the largest number of daily measurements (same as in Fig. S4).

c. The authors claim that the RF model captures the plume paths and magnitude and point to Figure S6. However, it is difficult to determine this without a side-by-side comparison to the observed values. It is important to substantiate claim because authors suggest that the RF model is well-suited to analyzing dust in Europe and suggest that this model is superior to those previously published.

We thank the reviewer for this comment and for highlighting the need to substantiate the model's performance. To clarify, the superior performance of our random forest (RF) model in capturing dust concentrations is demonstrated in the updated Figure S2, which compares the performance of RF to the physical DREAM model. This figure now includes fractional bias, RMSE, and Pearson correlation values, showing that the RF model significantly outperforms one of the best-performing state-of-the-art physical models.

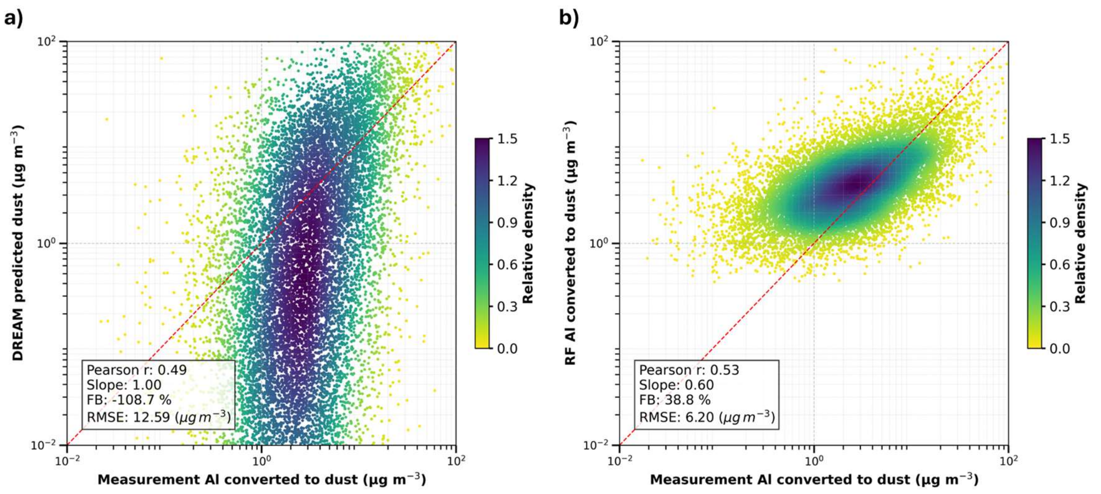

Fig S2 | Comparison between measured dust vs. (a) DREAM predicted dust, and (b) Random Forest (RF) predicted dust (b), along with relevant statistical metrics (Pearson correlation coefficient r, linear regression slope, fractional bias FB & root mean square error RMSE). Results are shown in a logarithmic scale and the color represents the relative density of measurements

Additionally, as shown in Figure S3 & S6, the RF model exhibits no significant temporal bias, enabling robust analysis of yearly trends. Figure S7, focusing on the extreme dust episode reported by Peshev et al. (2022), illustrates that the model accurately captures the timing, trajectory, and ground-level concentrations of a single event. Comparison to Figures 5 and 11 in Peshev et al. (2022) shows close agreement in plume path and ground-level concentrations (30–80 μg m^{-3}), supporting the model's suitability for analyzing dust frequency and intensity across Europe. We have updated the manuscript text to clarify these comparisons and to substantiate the description of Figure S7 accordingly in lines 213-216.

As a study case, the RF model captures the plume path and magnitude of the most severe and heavily studied Saharan dust events that hit the Balkans in April 2019, showing close agreement in plume path and ground-level concentrations (30–80 μg m^{-3})[64], supporting the model's suitability for analysing dust frequency and intensity across Europe.

We believe Figures S2, S3, S5, S6 & S7 demonstrate that the RF approach developed here provides a significant advancement over existing methodologies for European dust analysis.

3. Health Impact assessment. As currently constituted, the health impact analysis is lacking detail and feels like an afterthought. To justify its inclusion, additional information is needed, including:

a. The authors mentioned that their information regarding all-cause mortality and hospitalizations comes from Stafoggia et al. 2016. Were any other sources of information

considered? For example, how does the Stafoggia links to all-cause mortality compare with those from meta analyses such as that in Pouri et al, 2024 (https://doi.org/10.1016/j.scitotenv.2023.168945).

We thank the reviewer for raising this important point. While the meta-analysis by Pouri et al. (2024) was considered at an early stage, it was not used as the primary source because it pools data from many locations worldwide, including studies from East Asia and the USA. Populations in these regions may experience different dust types and compositions, which can influence the observed health effects.

In contrast, Stafoggia et al. (2016) provides pooled estimates from multiple European locations—including both Western and Eastern Mediterranean cities—making it directly relevant to our European-focused study. Given this, we based our primary assessment on the parameterization reported by Stafoggia et al.

That said, we acknowledge the value of comparing our estimates to the more recent meta-analysis. Accordingly, we repeated the mortality calculations using the relative risks reported in Pouri et al. and have included this comparison in the Extended Data. This provides readers with a direct evaluation of how the Stafoggia-based and Pouri-based estimates differ for our study domain. The updated Extended Data section "Health impacts from short-term exposure to dust" reads as follows (lines 576-595):

Health impacts from short-term exposure to dust

In order to estimate the increase in mortality due to short-term exposure to dust, we first calculated the fractional increase in mortality due to dust, based on the increase in risk for all-cause mortality (IR) due to acute dust exposure provided by Stafoggia et al[84], per 10 μg m^{-3} increase in dust exposure. The corresponding IRs were 0.65%±0.02% for all-cause mortality with a 0-1 lag day, 0.7%±0.02% for respiratory hospitalizations of the older than 15 age group with 0-5 lag days and 2.47%±0.07% for respiratory hospitalizations for 0 to 14 age group with 0-5 lag days. This increase is the population weighted to account for the differences in population density. Assuming an exponential dose-response function, the percent increase in mortality or hospitalisations for a given grid area then becomes (Equation 6):

$$\%\ increase\ in\ mortality = 100\% * \sum_{i=1}^{\#points} \frac{population\ of\ cell\ i}{total\ population} * \left(1 - \frac{1}{e^{IR*(exceedance\ concentration_i)}}\right) \quad (6)$$

where *#points* is equal to the total number of grid cells in the area, *population of cell i* is the total population within the grid cell, *IR* is the increase in risk for all-cause mortality and

exceedance concentration$_i$ is the average exceedance concentration over the decade for that grid cell. For this calculation the South is defined as all the land surface of Europe below, and including, Milan (45° N), for which we calculate an increase of 0.67%±0.02% in all-cause mortality for exceedance days, a 0.73%±0.04% increase in daily respiratory hospitalizations among the older than 15 age group and a 2.55%±0.07 rise in daily respiratory hospitalizations among the 0 to 14 age group. The mortality calculations were repeated using the IR's from Pouri et al. 2024[85] and shown in Extended data table 1.

Extended Data Table 1 | Epidemiological analysis: Increase in all cause, cardiovascular and respiratory mortality using the relative risks reported in Stafoggia et al. 2016 and Pouri et al. 2024. For the case of all-cause mortality Pouri et al. 2024 report the IR for PM_{10} during dust day rather than desert PM as Stafoggia.

	**Stafoggia et al. 2016**	**Pouri et al. 2024**
**Natural/All-cause**	0.67%±0.02	1.97%±0.06
**Cardiovascular**	1.14%±0.03	1.07%±0.03
**Respiratory**	1.33%±0.04	1.06%±0.03

b. It is important to state that these estimated increases in risk are per 10 ug/m3 increase in exposure to dust PM10 and that the Stafoggia results are reported as lag 0-1 results for all-cause mortality and lag 0-5 results for respiratory hospitalizations. This is an important distinction to make, and authors need to more explicitly state how they accounted for this in their estimates.

The reviewer is correct – we corrected this oversight in lines 577 to 583 as follows:

In order to estimate the increase in mortality due to short-term exposure to dust, we first calculated the fractional increase in mortality due to dust, based on the increase in risk for all-cause mortality (IR) due to acute dust exposure provided by Stafoggia et al[84], per 10 µg m-3 increase in dust exposure. The corresponding IRs were 0.65%±0.02% for all-cause mortality with a 0-1 lag day, 0.7%±0.02% for respiratory hospitalizations of the older than 15 age group with 0-5 lag days and 2.47%±0.07% for respiratory hospitalizations for 0 to 14 age group with 0-5 lag days.

c. The authors need to clarify where the information regarding adult vs. adolescent health impact comes from. From a cursory look at Staffogia et al. 2016, it seems that they separated out hospitalizations as individuals 0-14 and individuals aged 15 years and older. This delineation does not follow accepted definitions of adults vs. children or adolescents.

We now clarify that the separation is age based rather than the adult/non-adult split in accordance with the original paper. It is now explicitly mentioned that the

analysis refers to individuals belonging either to the 0 to 14 or older than 15 years. Lines 407 to 413 now read as follows:

We estimate that short-term exposure to such dust concentrations would be associated with a 0.67%±0.02% contribution to daily mortality and a 0.73%±0.04% contribution to daily respiratory hospitalizations during dust events among patients older than 15 years[84] (see Methods section, using global relative risk coefficients[85] leads to similar results presented in Table E1). The impact on younger patients is even more pronounced, with a contribution of 2.47%±0.07% in daily respiratory hospitalizations during dust events, highlighting more severe impacts of dust on vulnerable populations.[84]

d. If there is truly a potential impact on these health outcomes, more information is needed regarding methods and whether there has been change over time, which would help to make the case for the link to climate change made by the authors in the beginning paragraphs.

The reviewer is correct – in our analysis, the increase in dust concentrations observed over the past decade did not translate into measurable rises in health outcomes. However, if this trend persists, it could indeed lead to additional health impacts in the future. More importantly, our results highlight that current dust levels are already associated with a 0.67%±0.02% increase in mortality and substantially hinder progress toward achieving the European target air quality standards for $PM_{2.5}$ and PM_{10}—accounting for approximately 30% of their annual limit values.

To avoid any confusion, we have revised the text in the manuscript as follows in lines 413-416:

While the increase in dust concentrations observed during the 10-year study period was not sufficient to cause substantial changes in health outcomes, continued rises in dust levels could exacerbate these health risks.

Minor comments:

• The authors mention the use of data from multiple sources that were harmonized to a 10km x 10km spatial resolution, but very little detail is given regarding the specifics. Also, because authors include a health component, it is necessary to utilize methods for population weighting, which are not apparent from the text. Authors repeat the interpolation using a population weighting approach and more complete methods need to be included in the supplement.

We thank the reviewer for this valuable comment. Regarding the health impact assessment, we confirm that all health-related results were population-weighted, as described in Formula 2 of the Methods section.

The input datasets used in this study originated from multiple sources and came at different native spatial resolutions, which required harmonization to a common resolution. For instance, the DREAM surface dust concentration model outputs were

available at a resolution of 36 × 36 km, while land-use data were at finer scales. To ensure consistency, all datasets were first harmonized to the common 10 × 10 km grid using cubic interpolation; an appropriately fine resolution for a large-scale phenomenon. We now provide information about the inputs in Tables S3 & S4.

Applying population weighting during the interpolation of individual inputs would not have been appropriate, as the datasets differ in resolution and nature (e.g., meteorological, chemical, and demographic). Instead, population weighting was applied at the endpoint stage—after dust concentrations had been estimated—yielding a more representative and accurate estimate of population exposure.

Table S3 | Model variables used in this study.

Variable	Resolution (km)	Type	Source
**10-meter wind speed**	5.5	reanalysis	CERRA sub-daily regional reanalysis
**10-meter wind direction**	5.5	reanalysis	CERRA sub-daily regional reanalysis
**2-meter temperature**	5.5	reanalysis	CERRA sub-daily regional reanalysis
**total precipitation**	5.5	reanalysis	CERRA sub-daily regional reanalysis
**Dust aerosol optical depth at 550 nm (duaod550)**	83.325	reanalysis	CAMS global reanalysis (EAC4)
**DREAM**	36.663	forecast	WMO Barcelona Dust Regional Center

• The authors state that the RF model effectively captures major and minor dust intrusions, but little information is given regarding how they assess this. How was this determined?

The statement has been reformulated to be more accurate, with mentions of intrusions removed in accordance with comments from other reviewers as well. Lines 199 to 203 now read:

The Random Forest (RF) model effectively captures daily dust concentrations, yet overestimates concentrations below 0.1 μg m^{-3} and underestimates the highest concentrations above 10 μg m^{-3} (Fig. S2b), leading to an overall positive bias (fractional bias: 38.8%). When averaged annually, the model shows much better agreement with the measurements (fractional bias: 38.8%), making the model well-suited for long-term trend analysis (Fig. S3).

• The article also gives information regarding the PM2.5 to PM10 ratios. However, there is little explanation of how this pertains to the model or was used to inform estimations. Please justify the inclusion of information regarding the processing time for the models. This seems out of place and should be excluded from the main text of the manuscript.

This is a helpful comment and we acknowledge that our initial description of how the $PM_{2.5}$ concentrations were determined was not sufficiently clear. These

concentrations were directly derived based on $PM_{2.5}/PM_{10}$ ratios from observations at monitoring sites where both PM_{10} and $PM_{2.5}$ elemental measurements were available, rather than from the physical or Random Forest models. This observational approach inherently accounts for the average atmospheric processing time between emission, transport, and receptor, providing a realistic representation of the dust size distribution reaching the measurement sites.

We believe that including this information is important, as it allows for a direct comparison between estimated dust concentrations and the regulatory $PM_{2.5}$ air quality limit values. To clarify this point, the text in the main manuscript has been revised as follows in lines 132 to 134:

While dust is mostly present in the coarse mode, based on the observed $PM_{2.5}/PM_{10}$ elemental ratios at locations where both size fractions are available, we estimate that 27.7% of dust is present as $PM_{2.5}$.

In the updated version of the manuscript, we have also modified the sentence in the last paragraph as:

By 2021, our model shows that in Southern Europe dust already accounted for 31% of WHO[81] annual mean PM_{10} guideline values (15 µg m^{-3}) and 25.8% (calculated based on observed $PM_{2.5}/PM_{10}$ elemental ratios) of $PM_{2.5}$ guideline values (5 µg m^{-3}), significantly hindering these regions to comply with regulations.

• Throughout, authors do not spell out multiple abbreviations and acronyms. Since Nature reaches a larger audience, it is important to introduce the elemental abbreviations as well as the acronyms for DREAM, CHIMERE, CORINE, etc.

We thank the reviewer for pointing this out. Acronyms have now been spelled out upon first use wherever possible throughout the manuscript. We would like to note though that CHIMERE and SKIRON are not acronyms and thus cannot be spelled out.

• Figure 1b seems unnecessary since this information is easily gleaned from the text and should be excluded or moved to the supplement.

While the reviewer is correct that this information is included in the text, this figure still has added value since it offers a visualization of the variance of dust ratios in dust events. For an easier comparison with literature reported values we now provide additional information (literature values and results from sensitivity tests).

Next, we determine elemental ratios characteristic of transported dust by applying zero-intercept linear regression to a subset of data with high *Al* concentrations (here >1 µg m^{-3}; details in method section ‘ Uncertainty of elemental ratios and impact on dust estimate’) - indicative of elevated transported dust contributions - and find *Si*:*Al*, *Ti*:*Al*, *Ca*:*Al*, and *Fe*:*Al* ratios of 2.61±0.033, 0.068±0.003, 1.58±0.099, and 0.85±0.052 respectively (Fig. 1b). While standard linear regression would provide a better fit of the data it would not be physically consistent, since, for pure dust, if one specie was zero then all would have to be

zero and a non-zero intercept regression violates that principle. These elemental ratios align well with reported values for dust transported to Europe[42-46] and North Africa source estimates[44], acknowledging variability in elemental composition depending on the emission area, particularly for Calcium. Despite this variability, given the extensive spatial and temporal coverage of our dataset across urban and rural environments, these ratios provide the the most comprehensive observational characterization of the average elemental composition of transported dust in Europe to date[47].

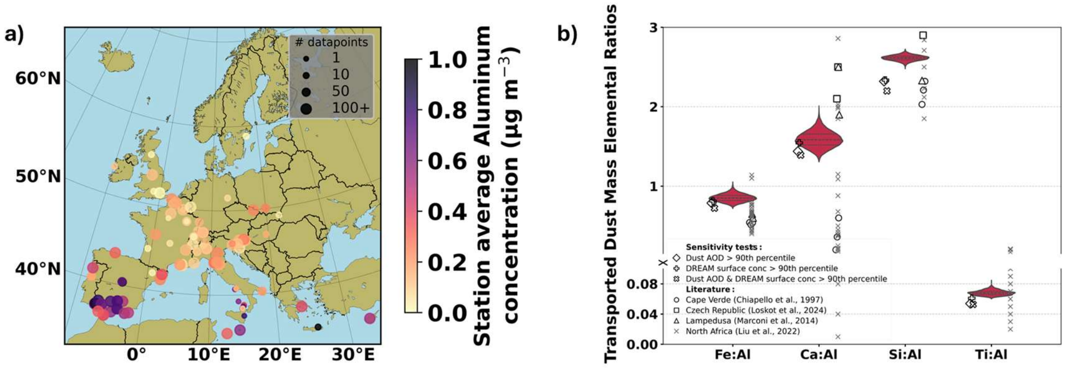

Fig 4 | Dust observations and elemental ratios of transported dust in Europe. a) average aluminium concentrations for 103 locations in Europe. The size of the dot corresponds to the number of daily measurements, while the color to the average concentration of *Al* at this location. High *Al* concentrations show the large impact of transported dust on air quality in Southern Europe. b) Violin plots of elemental ratios of transported dust, estimated via bootstrapped zero-intercept linear regressions applied to daily data with elevated transported dust concentrations (aluminium concentrations >1 μg m^{-3}). The diamonds, crosses and x's correspond to filtering to the 90th percentile of optical depth, DREAM concentration and both. Circles, squares and triangles represent literature values from Chiapello et al 1997[5], Loskot et al. 2024[7], Marconi et al. 2014[6] for dust arriving to Europe, while North Africa source estimates from Liu et al., 2022[3] are denoted with the gray x's.

• Throughout, some of the color schemes are either difficult to read or do not reflect the underlying data. For example, the scheme used in Figure S6, S7, and S9 makes it difficult to see the country boundaries due to the use of black as the extreme high values.

We apologize for this – plots have now been updated. Figure S7 has been removed due to not being used in the main text as another referee noted. Please find below the updated Figures

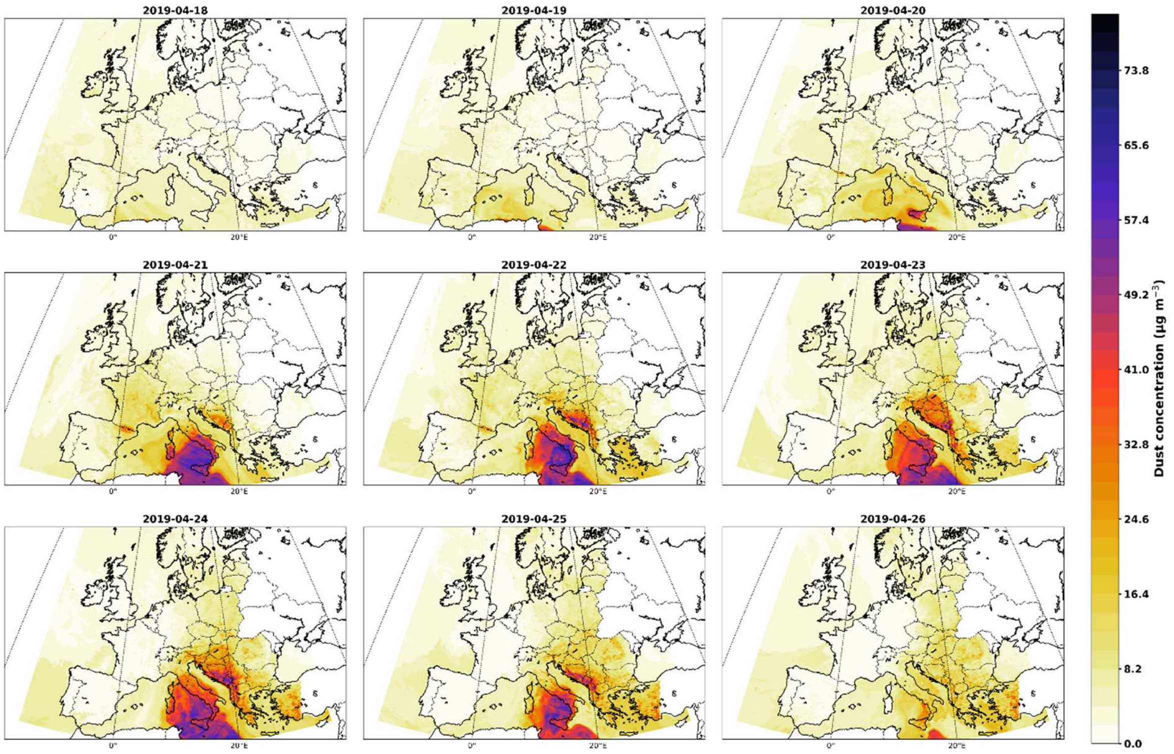

Fig S7 | Random forest–predicted dust concentrations (μg m^{-3}) showing the simulated transport trajectory of Saharan dust during the extreme event affecting the Balkans, as reported by Peshev et al. (2021), for the period 18 April 2019 (one day before the episode's onset) to 26 April 2019 (episode end).

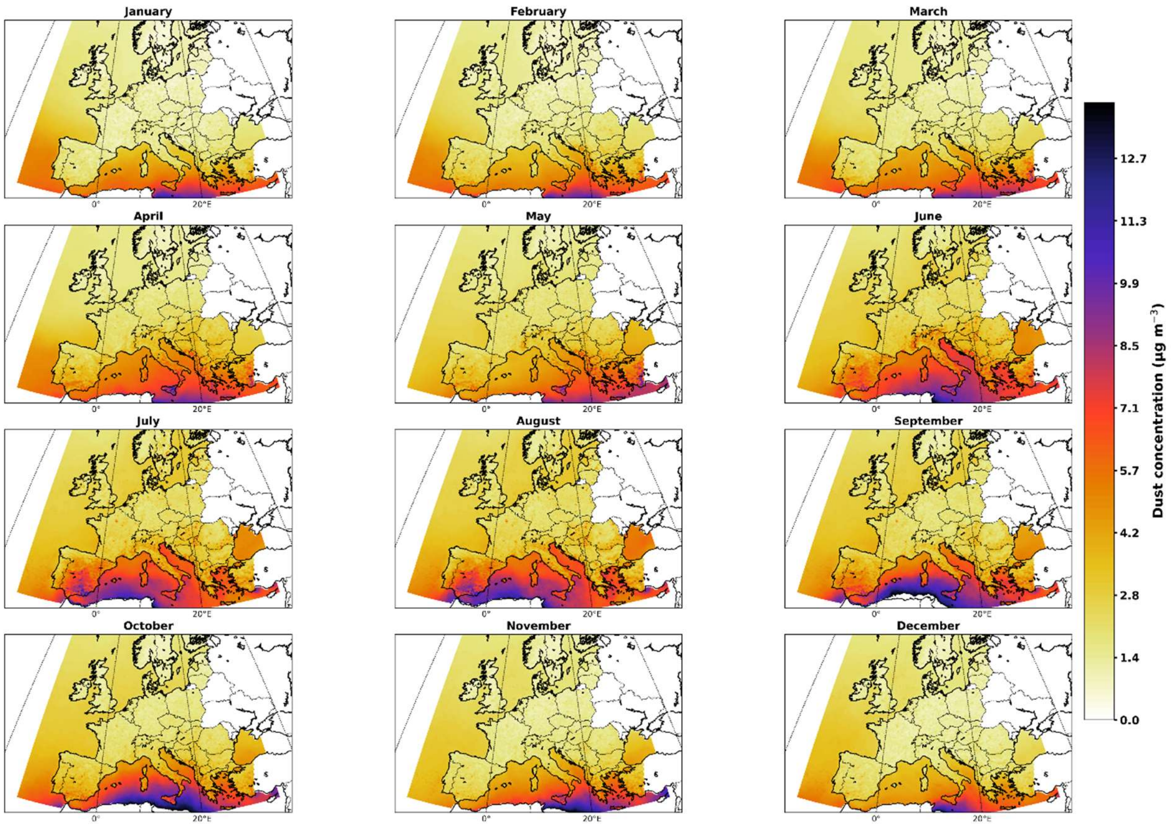

Fig S8 | Seasonal variation of random forest–predicted dust concentrations (μg m^{-3}), shown as 10-year monthly averages over the study domain.

Referee #3 & 4 (Remarks to the Author):

Review of manuscript by P. Vasalikos et al. ''Rising dust pollution threatens European air quality in a changing climate''

Reference: Nature ms 2025-04-10703

Note: You will find the review from [co-reviewer] in black letters and the review from [main reviewer] in blue letters.

The paper provides analysis of dust concentration and its spatio-temporal distribution for the period 2012-2021 over Europe and leverages on a database of elemental compositional data, machine learning model and ice core data. The key results indicate that while the frequency of dust events has not increased, its severity has increased over Europe. It is suggested that atmospheric circulation patterns have affected variations in dust concentrations over the last decade. Analysis of data from an Alpine ice-core combined with different climate variables describing global, hemispheric, and regional climate patterns, such as the NAO or the MOI, support a significant increase in dust load in the last decade compared to 1750-1850 period as associated to desertification in Northern Africa. Analysis is done to relate a rise in mortality to dust events in Europe.

The paper is original in its combination of chemical datasets with machine learning approach to investigate extensively the dust distribution, trends, and relation to air quality in Europe and analyse the drivers of identified concentrations and tendencies. This is an important topic of relevance under climate changing conditions and air quality regulations. The context is described in a sufficient clear and balanced manner and the abstract and conclusions are appropriate.

We thank the reviewer for their kind comments.

Data and methodology are sound but the quality of data and the significance of results are difficult to assess for different reasons. First, not many details are provided in the methods and supplementary concerning measurement protocols for metal data, when different techniques are considered for the various sites. How the different datasets are harmonized and unbiased is of relevance when combing such a large dataset. It is mentioned "a great care in flagging data" but this is not sufficient to assess the data quality.

We thank the reviewer for this comment. As noted by another reviewer, the combination of datasets from different measurement techniques requires careful consideration. All elemental data used in this study were obtained using well-established methods in atmospheric aerosol research: X-ray Fluorescence (XRF, including Xact), Particle-Induced X-ray Emission (PIXE), and Inductively Coupled Plasma analyses (ICP). Numerous studies have demonstrated high inter-method comparability for Al, with differences typically <10% and correlation coefficients R^2

> 0.9 (e.g., Gini et al., 2021; Chiari et al., 2018; Lucarelli et al., 2011; Saitoh et al., 2002), supporting the direct comparability of these measurements for trend analysis.

After aggregation, we applied harmonization and quality control steps: values below the detection limit (Al < 0.003 µg m^{-3}) were removed; repeated or rounded data at detection limits were filtered; records with time resolution >1 day were excluded; and hourly data were averaged to daily means. Known biases in Xact-derived Al data were corrected using Ti concentrations and Ti/Al ratios derived from the harmonized database.

No additional calibration, normalization, or harmonization was applied beyond the providers' QA/QC procedures. To explicitly test for potential biases due to heterogeneous techniques, we grouped RF model performance by measurement method (XRF, PIXE, ICP, Fig. S12). Consistent performance across methods indicates no significant systematic bias.

Taken together, these steps—supported by literature evidence, harmonization procedures, and model-based evaluation—ensure that the dataset is robust, reliable, and suitable for the analyses presented in this study.

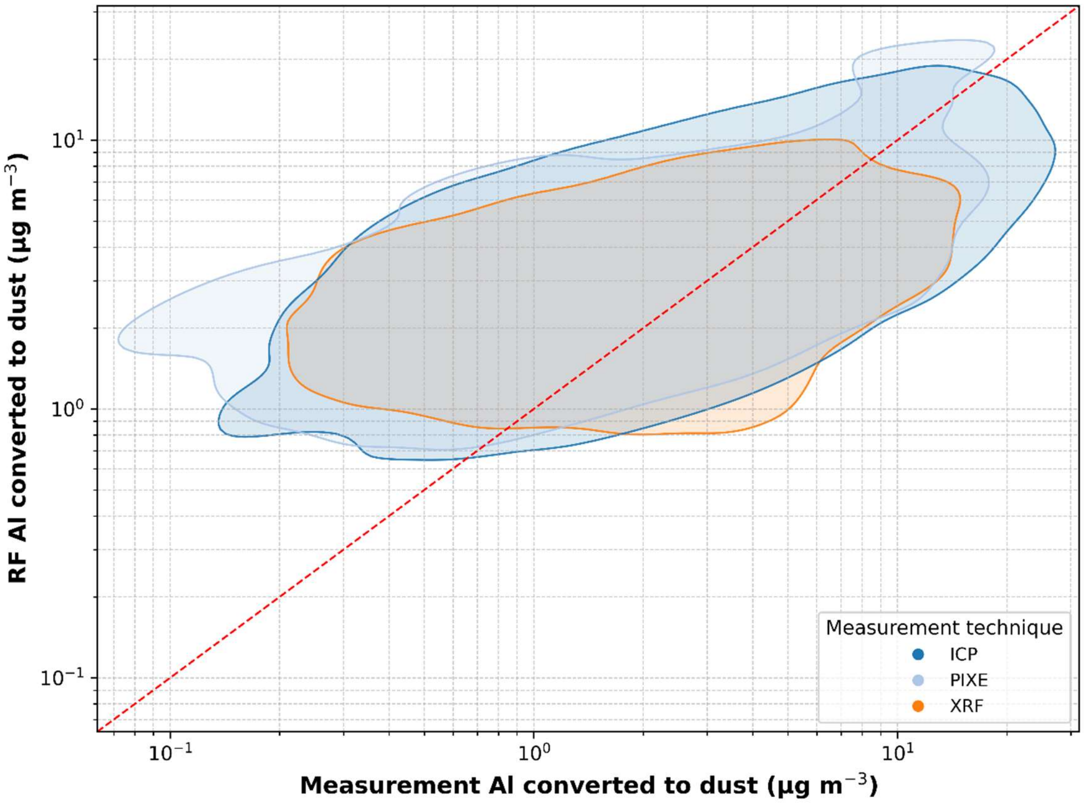

Fig S12 | Density plot for 90% of predictions, grouped by technique, for the leave-1-out results.

We have now amended the "Dataset and database construction" in the Method section as follows:

Dataset and database construction

We compiled an extensive database of daily average measurements of PM_{10} elements related to dust (Al, Ti, Si, Ca, Fe) from all over Europe, to estimate dust concentrations. The bulk of the data was compiled ad-hoc for this study, while some was extracted from the EBAS database[84]. The most frequently measured metal that is prevalent in mineral dust was aluminium; measurement data for other dust metals found purely in dust such as silicon and titanium were much scarcer. In contrast, local sources such as road dust as well as non-exhaust traffic and construction emissions, affect calcium and iron, rendering them unsuitable for estimating transported dust in this study. Metal concentrations were measured both with offline filter-based techniques (ICP, PIXE & offline XRF)[85] and online techniques (online XRF XACT)[86,87]. Al measurements from these techniques are directly comparable for the purposes of trend analysis, since numerous inter-laboratory comparison studies have shown that these techniques yield highly consistent results for particulate elemental concentrations[88-91]. In addition, grouping model random forest cross validation results by training technique (XRF-based, ICP-based or PIXE – Fig. S12), does not indicate any inherent biases between the techniques. Great care was taken in curating the database by flagging data below detection limits and working with data providers to ensure high data quality After data were aggregated from all providers various methods were employed to ensure data quality. Low concentration values at the detection limit were discarded. To that end, concentration values below a detection cutoff of Al = 0.003 $\mu g\ m^{-3}$ were removed. In addition, values that were repeated more than 3 times in sequence or repeated more than 5 times (at a third decimal precision) were interpreted as data that was replaced by the detection limit. Further data with a time resolution > 1 day were discarded, while hourly data were aggregated to daily averages.

A second aspect concerns the methodology related to the spatial distribution of used metal data (Fig. 1) which are unevenly distributed across Europe, but mostly representative of specific regions as Spain, France or Northern Italy. Other regions, in particular Eastern Europe and Scandinavia are not represented by the compositional dataset and makes difficult to evaluate the reliability of the RF approach to such large areas.

We thank the reviewer for raising this important point. We fully acknowledge that the spatial distribution of metal measurements across Europe is uneven, with dense coverage in Southern and Western Europe (e.g., Spain, France, Northern Italy) and sparse coverage in Northeastern Europe, the Balkans, and Scandinavia. Unfortunately, to our knowledge no long-term elemental measurements suitable for dust quantification exist for these regions during the study period.

At the same time, our dataset extends further than Fig. 1 may suggest: it spans eastward to Greece and Cyprus and northward to much of England. The regions that are underrepresented—particularly Northeastern Europe and Scandinavia—are climatologically those with the lowest Saharan dust influence, especially in comparison to southern Europe where dust episodes are more frequent, intense, and better constrained observationally. In this sense, the observational gaps coincide with areas where dust concentrations are inherently low, thereby reducing their impact on the RF model's predictive skill.

We also emphasize that insufficient compositional monitoring is a widely recognized issue. Eastern Europe in particular lacks observational data not only for metals but for many air-quality–relevant species. In line with the revised EU air quality directives, our study highlights the urgent need to establish additional long-term supersites—especially in undersampled regions—to ensure representative coverage of chemical composition and improve future large-scale analyses. We have now added a statement in the manuscript to explicitly acknowledge this limitation and to call for expanded measurements in Lines 120 to 124:

We note that the spatial coverage of metal measurements is uneven across Europe, with limited data availability in Northeastern Europe, the Balkans, and Scandinavia—regions that are also climatologically less affected by Saharan dust. This data limitation underscores the need for additional long-term supersite measurements in these areas, in line with current European air quality directives[41].

As third aspect, the discussion strongly lacks of uncertainty and statistical significance analysis. Statistical errors are not discussed or assessed at any point, which implies that most of the results are provided without evaluation of its statistical significance, uncertainty or fluctuation. This is an important lack that compromises the quality of the work all along the paper. Ex., the elemental ratios expected to represent dust transport (line 121) are provided without indication of uncertainty, which is not at a good scientific standard.

The analysis of the dust concentrations in Europe also lack any indication of regional fluctuations and data variability, As an example in line 210 the north and south Europe dust concentrations are provided as central values without indication of their variability, which affects the capacity to evaluate and assess their differences. Further example is the analysis of the increase in daily mortality, where a result value of +0.51% is provided but no indication of the robustness this value reported or discussed. For these reasons, while the reasoning and figure discussion and interpretation is sound, the reviewer evaluation is that this lack of consideration of uncertainty and the identified methodological aspects, the conclusions are not fully supported and the paper would require modifications to meet the required standards.

We thank the reviewer for pointing this oversight. We now provide values with the corresponding uncertainty/deviation when appropriate all throughout the text, including the calculations for acute health effects. In accordance with another reviewer's comment, quantifying the uncertainty of dust estimates can be done by calculating the uncertainty of the ratios (defined here as one standard deviation for the ratios of individual points) and substituting them in the dust formula (2.2Al+2.49Si+1.63Ca+1.94Ti+ 2.42Fe = 13.5Al). Specifically, the uncertainties for each ratio are $\sigma_{Ti:Al}$=0.003, $\sigma_{Si:Al}$=0.033, $\sigma_{Fe:Al}$=0.052, $\sigma_{Ca:Al}$=0.1, corresponding to a total dust uncertainty of 0.377, meaning that total dust belongs in the range of 13.12Al to 13.88Al. It should be noted that the greatest uncertainty comes from Fe and Ca that have substantial local contributions. This information has been added to a new section in the Methods section, titled "Uncertainty of elemental ratios", on lines 373-380, to investigate both the inherent uncertainty of the numbers provided in the

manuscript, as well as the assumptions we made in estimating dust concentrations from a single tracer.

Uncertainty of elemental ratios and impact on dust estimate

We quantified the impact of uncertainty in the elemental ratios—defined here as one standard deviation from the bootstrap results shown in Fig. 1b (Si:Al 2.61±0.033, Ti:Al , 0.068±0.003, , Ca:Al 1.58±0.099, and , Fe:Al 0.89±0.052)—on the dust estimates using uncertainty propagation based on the formula: 2.2Al + 2.49Si + 1.63Ca + 1.94Ti + 2.42Fe. This results in a total dust estimate of (13.5 ± 0.377) × Al. Notably, the largest contributions to the overall uncertainty stem from Fe and Ca, which are also influenced by substantial local sources. Zero-intercept fits were used for element–element plots (e.g., Ca concentration vs. Al concentration) to estimate dust-phase mass-based elemental ratios under the assumption that—after blank correction and removal of non-dust contributions—both analytes originate from the same dust endmember. In that case, when the dust contribution vanishes (Al → 0), the co-emitted element should also vanish (Ca → 0), implying a physically meaningful intercept of zero. In addition, we tested a linear model with an intercept using bootstrap analysis and found that zero lies within one standard deviation of the mean intercept (intercept ± σ_intercept), supporting the use of a zero-intercept model. Given this result, along with the physical consistency and practical advantages of a zero-intercept regression—such as enabling the use of average ratios to estimate other components—we opted for the zero-intercept model. A summary table of the relevant statistics is provided in the Supplement (Table S2).

Elemental ratios on days with low amounts of transported dust can be substantially affected by local sources. With a rather high Aluminium concentration threshold of >1 ug/m3 for determining the transported dust elemental ratios, we aimed at minimizing the impact of local sources on the elemental ratios. In addition, we performed a sensitivity analysis varying the threshold showing that the elemental ratios vary with cutoffs above 0.5 for Ca:Al and 0.75 for Fe:Al within the 1 standard deviation of the value obtained with a cutoff of 1 ug/m3. Further, we investigated model-driven criteria for dust episodes instead of using a static Aluminium concentration cutoff using the DREAM and optical depth values and only including data points that were higher than the 90th percentile in those (each alone or both at the same time), as a proxy for dust events. This approach resulted in similar values (Fig. 1b & S13).

The uncertainty analysis of the mass-based transported dust elemental ratios (main text and Fig. 1) was further expanded and now includes additional sensitivity tests and further literature values both from Europe as well as North Africa source estimates.

These elemental ratios align well with reported values for dust transported to Europe[42-46] and North Africa source estimates[44], acknowledging variability in elemental composition depending on the emission area, particularly for Calcium. Despite this variability, given the extensive spatial and temporal coverage of our dataset across urban and rural environments, these ratios provide the the most comprehensive observational characterization of the average elemental composition of transported dust in Europe to date[47].

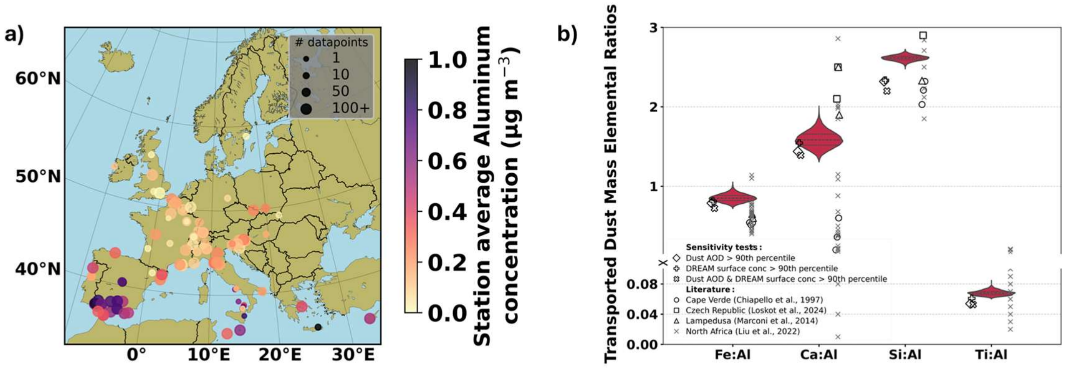

Fig 5 | Dust observations and elemental ratios of transported dust in Europe. a) average aluminium concentrations for 103 locations in Europe. The size of the dot corresponds to the number of daily measurements, while the color to the average concentration of *Al* at this location. High *Al* concentrations show the large impact of transported dust on air quality in Southern Europe. b) Violin plots of elemental ratios of transported dust, estimated via bootstrapped zero-intercept linear regressions applied to daily data with elevated transported dust concentrations (aluminium concentrations >1 μg m^{-3} for Fe:Al and Ca:Al). The diamonds, crosses and x's correspond to filtering to the 90th percentile of dust optical depth, DREAM dust surface concentration and both. Circles, squares and triangles represent literature values from Chiapello et al 1997[46], Loskot et al. 2024[48], Marconi et al. 2014[47] for dust arriving to Europe, while North Africa source estimates from Liu et al., 2022[44] are denoted with the gray x's.

In addition, we validated the trends through bootstrapped linear regression, where we consider a gridcell's trend significant if there is no sign change between the 25th and 75th percentile of the bootstrap results (Figs 3, 4 & 5 captions). It has now been explicitly mentioned throughout the text in addition to the clarifications in the figure captions. Further, we have now applied this type of uncertainty analysis also to the correlation maps highlighting the drivers of the short-term year-to-year variations in dust concentrations.

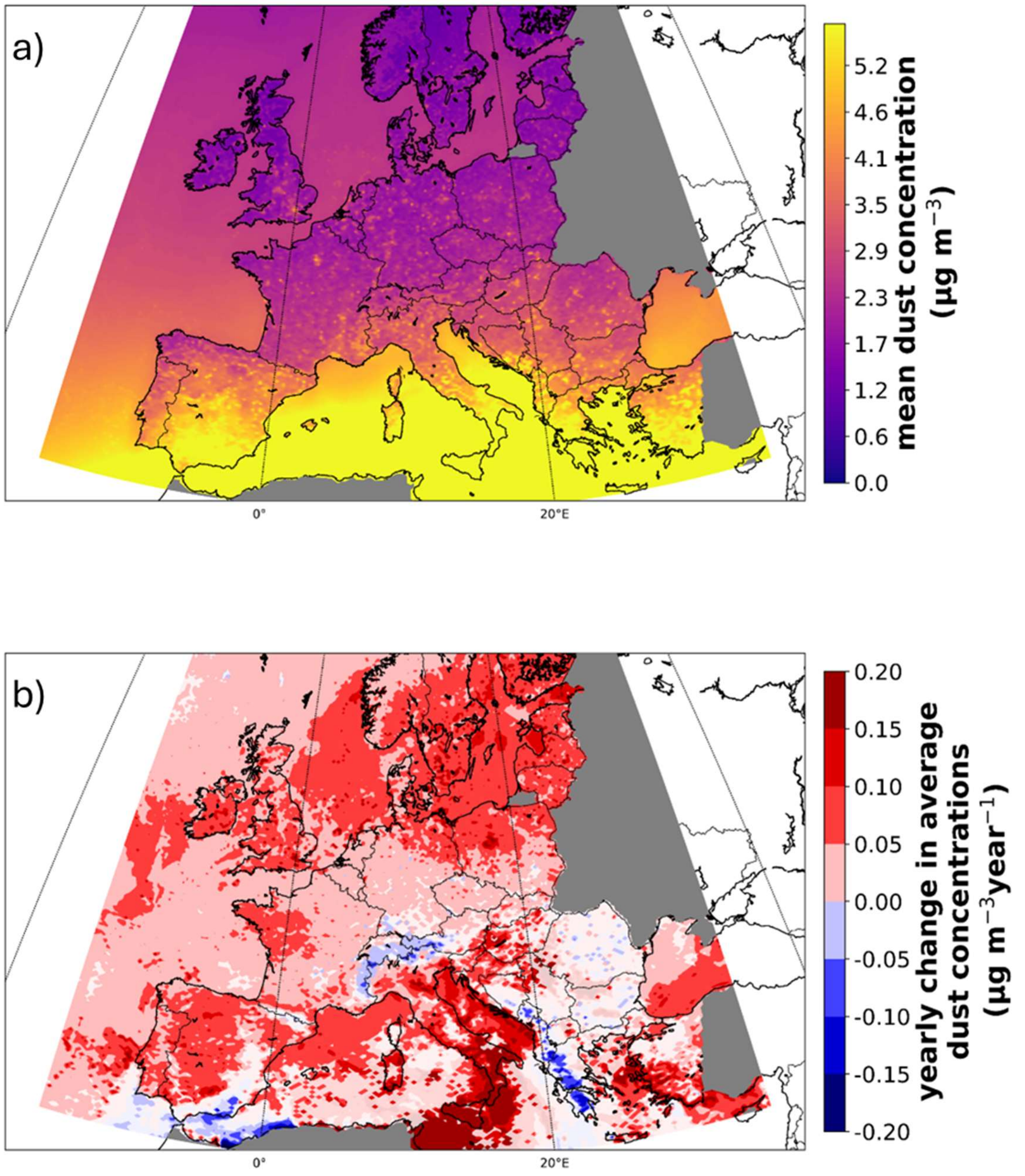

Fig 3 | Dust concentrations and their changes in Europe during the period 2012-2021: mean dust concentrations (a) and trend (b). Concentrations are reported in $\mu g\ m^{-3}$ and their corresponding trends in $\mu g\ m^{-3} year^{-1}$. A trend is defined as the mean of a 100 times bootstrapped linear regression for the variable of interest, and shown on the map if there is no sign change between the 25th and 75th percentile of the bootstrap results. Areas where a sign change occurs are denoted with white (for regional means considered zero), while areas where no land use data exists are denoted in gray.

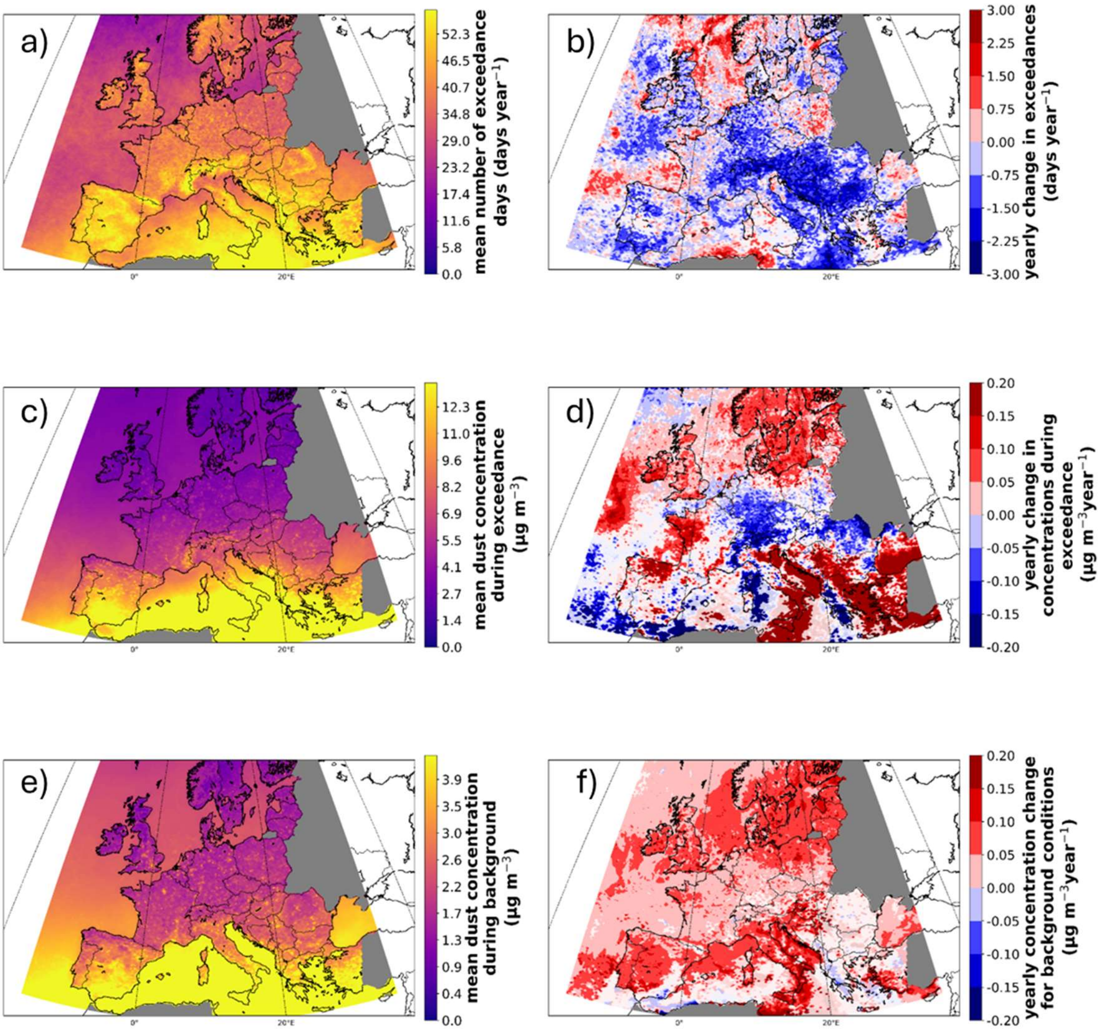

Fig 4 | Dust episodes and their trends in Europe during the period 2012-2021: mean number of days where a dust exceedance occurs (a) and trend (b), mean dust exceedance concentrations (c) and trend (d) & mean dust concentrations during background (e) and trend (f). Concentrations are reported for PM_{10} in µg m^{-3} and their corresponding trends in µg m^{-3}year^{-1}, while the number of exceedance days is reported as days with their corresponding trend reported as days year^{-1}. A trend is defined as the mean of a 100 times bootstrapped linear regression for the variable of interest, and shown on the map if there is no sign change between the 25th and 75th percentile of the bootstrap results. Areas where a sign change occurs are denoted with white (for regional means considered zero), while areas where no land use data exists in gray.

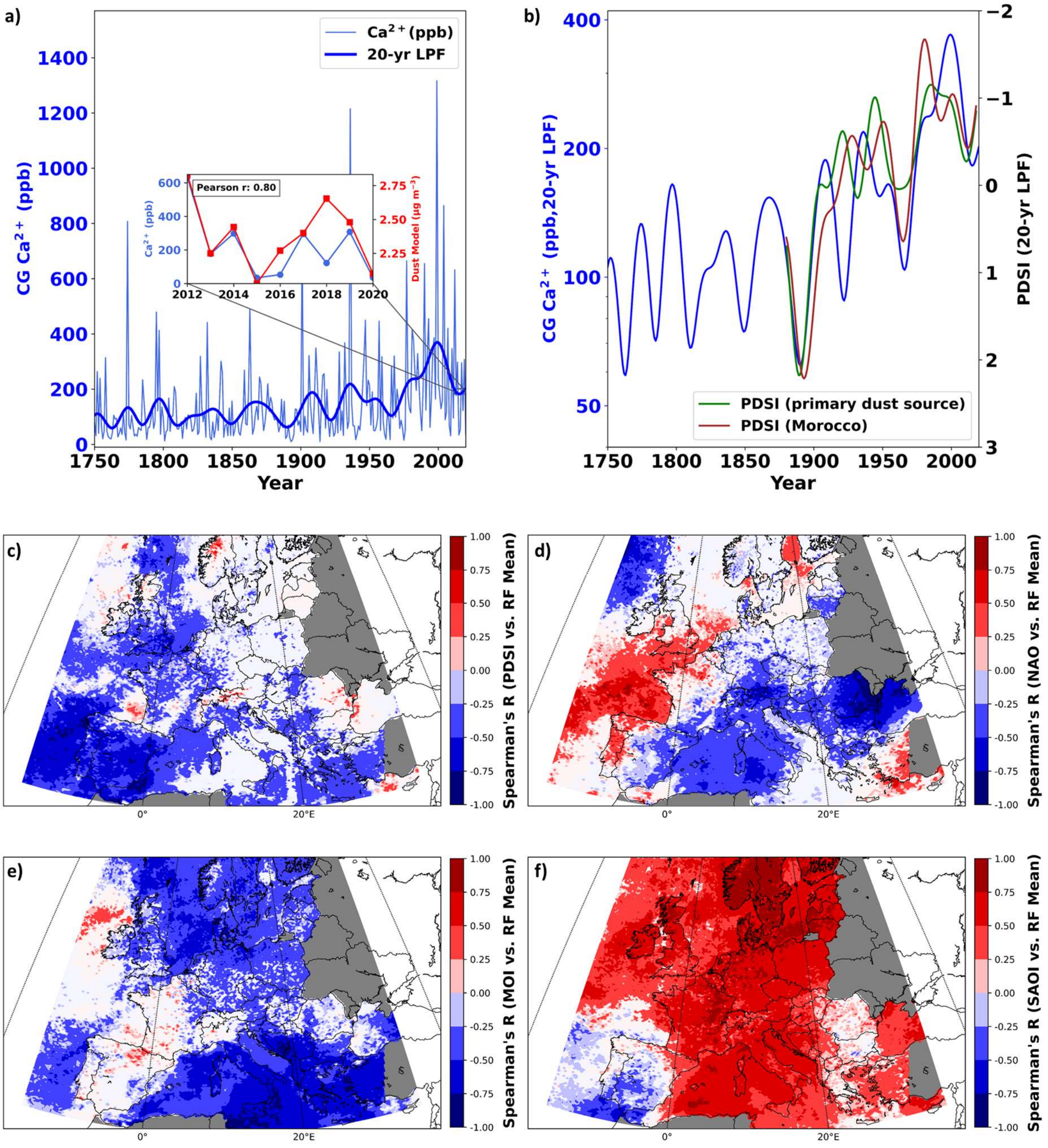

Fig 5 | Long-term climatological trends and short-term variations in dust: Timeseries of Colle Gnifetti ice core Ca^{2+} concentrations (annual: blue green line, 20-year low pass filtered: blue bold line) from 1750 to 2020 and comparison with model results for the period 2012-2020 (inset plot) (a), 20-year low pass filter Ca^{2+} concentrations (blue) and the Palmer Drought Severity Index (PDSI) in the major source region of dust for the Swiss-Italian Alps in the Northwest Sahara (-7.14 – 11.27°E, 32.15-35.66°N - green) and (-10 - 0°E, 30-35°N - brown) (b) along with the spatiotemporal correlation between annual mean dust concentrations in the period 2012-2021 and PDSI in the extended Sahara area (14.2° to 30.17° N, -9.15° to 32.52° E) (c), North Atlantic Oscillation Index (NAO) (d), Mediterranean Oscillation Index (MOI) (e) and Saharan Oscillation Index (SaOI) (f). Results for the correlations are presented as the mean of a 100 times bootstrapped linear regression for the variable of interest and Random Forest (RF) results, and shown on the

map if there is no sign change between the 25th and 75th percentile of the bootstrap results. Areas where a sign change occurs are denoted with white (for regional means considered zero), while areas where no land use data exists are denoted in gray.

We also updated the maintext such that the uncertainty of the mean dust concentrations and their trends (and their impacts on health assessment) is reported. and a paragraph on their calculation is added to the methods under 'Uncertainty of elemental ratios and dust estimate':

The uncertainty σ_{mean} in modelled yearly mean dust concentrations $dust_{mean}$ (including exceedance and background concentrations) is estimated by accounting for two main components: (1) the uncertainty in the derived dust/Al ratio (RE_{ratio} = 0.377/13.5=2.7%), and (2) the relative root mean square error of the modelled annual mean dust concentrations ($RRMSE_{annual\ mean}$ = 0.45; Fig. S3), as determined through a leave-one-out performance analysis against chemically derived dust concentrations from in-situ measurements (Eq. 1).

$$\sigma_{mean} = \sqrt{(RRMSE_{annual\ mean} * dust_{mean})^2 + (RE_{ratio} * dust_{mean})^2} = \sqrt{(RRMSE_{annual\ mean})^2 + RE_{ratio}^2} * dust_{mean} \quad (1)$$

The robustness of dust concentration trends over the 10-year study period was evaluated by bootstrapping the linear regression of annual dust concentration time series at each grid cell (100 bootstrap resamples). The reported trend t_{dust} represents the mean of the bootstrap-derived slopes, where grid cells where the interquartile range includes zero are masked in white (zero trends are assumed for regional means in such cases). The overall uncertainty $\sigma_{trend,conc}$ in the trend (Eq. 2) includes both uncertainty in the dust/Al ratio and the bootstrap-based variability in fitting the trend (RE_{boot}, domain mean relative uncertainty excluding gridcells with non-significant trends), while the precision in the yearly dust prediction is already accounted for by the latter (see Eq. 1):

$$\sigma_{trend,conc} = \sqrt{(RE_{ratio} * t_{dust})^2 + (RE_{boot} * t_{dust})^2} = \sqrt{(RE_{ratio})^2 + (RE_{boot})^2} * t_{dust} \quad (2)$$

with $\sqrt{(RE_{ratio})^2 + (RE_{boot})^2}$ equal to 0.5, 0.23 & 0.4 for the trend in mean, exceedance and non-exceedance concentrations respectively.

Analogously, we estimate the uncertainty σ_{ed} of the number of exceedance days and related trends. We use counting statistics to describe the uncertainty of the number of days ($RE_d = 0.17$, being the domain mean relative uncertainty based on $\sqrt{days}/days$) (Eq.3).

$$\sigma_{ed} = RE_d * days \quad (3)$$

The domain mean relative uncertainty $\sigma_{trend,day}$ (excluding gridcells with non-significant trends), in the trend of exceedance days t_{days} is estimated via the bootstrap-based relative variability (RE_{db}=0.27) in fitting the trend (Eq. 4), which also accounts for the precision in the yearly prediction in exceedance days (see Eq. 3).

$$\sigma_{trend,day} = RE_{db} * t_{days} \quad (4)$$

Detailed comments:

Line 86: "decrease in anthropogenic pollution" a reference is required here

An new appropriate reference has been added.

Carslaw, K. S., Boucher, O., Spracklen, D. V., Mann, G. W., Rae, J. G. L., Woodward, S., and Kulmala, M.: A review of natural aerosol interactions and feedbacks within the Earth system, Atmos. Chem. Phys., 10, 1701–1737, https://doi.org/10.5194/acp-10-1701-2010, 2010.

Line 88: this should read "solar and terrestrial infrared radiation"

We corrected the statement.

Line 99: here a need to have consistent datasets across Europe is mentioned, but the spatial representativeness of sites across Europe considered in the paper does not respond to this need

We have addressed a similar concern about the lack of sites in North -Eastern Europe and Scandinavia in an earlier comment. We note that the database presented in this manuscript, from 103 sites and totaling over 18'000 daily elemental concentrations, is far more extensive than any existing dataset, covering the whole of Western Europe, and Eastern Europe. A new statement has been added in lines 120-124:

We note that the spatial coverage of metal measurements is uneven across Europe, with limited data availability in Northeastern Europe, the Balkans, and Scandinavia—regions

that are also climatologically less affected by Saharan dust. This data limitation underscores the need for additional long-term supersite measurements in these areas, in line with current European air quality directives[37].

Line 127-128: “these ratios provide the most robust characterization of transported dust elemental composition in Europe to date” is this true considering the representativeness of measurement sites considered?

We thank the reviewer for raising this point. Our statement refers to the breadth and diversity of observational constraints rather than implying absolute completeness. Most previous studies reporting elemental ratios of transported dust rely on either a small number of sites, short campaigns, or measurements taken primarily in source regions. In contrast, our dataset compiles transported-dust elemental ratios from more than 100 monitoring sites across Europe, spanning a wide range of meteorological regimes, transport pathways, and receptor environments. This makes our characterization substantially more comprehensive and representative than what has been available to date.

To address this comment more explicitly, we have revised the manuscript and expanded Figure 1b to include a detailed comparison with published elemental ratios from both European receptor sites and North African/Middle Eastern source regions. The literature shows considerable variability across locations and methods, and our multi-site European dataset captures this variability while providing a continent-scale, observation-based synthesis that has not been previously available. We also now clarify in the text that our ratios are “most comprehensive observational characterization of the average elemental composition of transported dust in Europe to date**” reflecting scope rather than implying that coverage is uniform across Europe.**

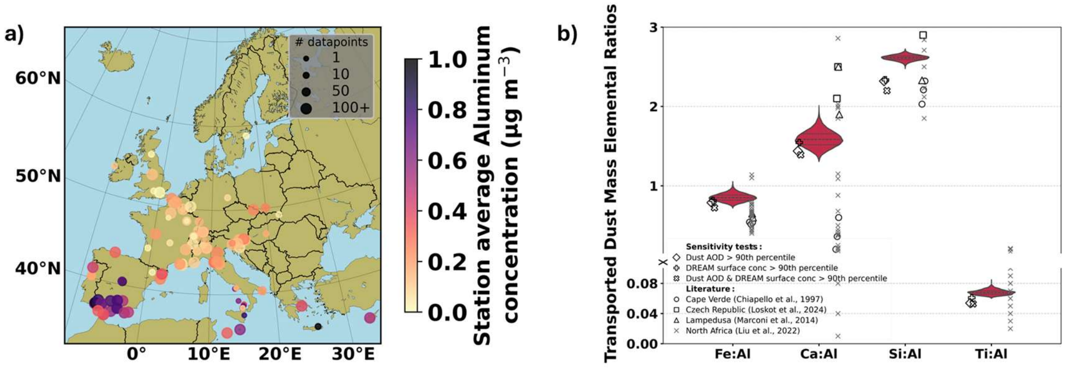

Fig 6 | Dust observations and elemental ratios of transported dust in Europe. a) average aluminium concentrations for 103 locations in Europe. The size of the dot corresponds to the number of daily measurements, while the color to the average concentration of *Al* at this location. High *Al* concentrations show the large impact of transported dust on air quality in Southern Europe. b) Violin plots of elemental ratios of transported dust, estimated via bootstrapped zero-intercept linear regressions applied to daily data with elevated transported dust concentrations (aluminium concentrations

>1 μg m^{-3} for Fe:Al and Ca:Al). The diamonds, crosses and x's correspond to filtering to the 90th percentile of dust optical depth, DREAM dust surface concentration and both. Circles, squares and triangles represent literature values from Chiapello et al 1997[46], Loskot et al. 2024[48], Marconi et al. 2014[47] for dust arriving to Europe, while North Africa source estimates from Liu et al., 2022[44] are denoted with the gray x's.

In addition to this, we have amended to text to reflect the reviewer's remarks and the results of the comparison, in lines 135-147:

Next, we determine elemental ratios characteristic of transported dust by applying zero-intercept linear regression to a subset of data with high *Al* concentrations (here >1 μg m^{-3}; details in method section ' Uncertainty of elemental ratios and impact on dust estimate') - indicative of elevated transported dust contributions - and find *Si*:*Al*, *Ti*:*Al*, *Ca*:*Al*, and *Fe*:*Al* ratios of 2.61±0.033, 0.068±0.003, 1.58±0.099, and 0.85±0.052 respectively (Fig. 1b). While standard linear regression would provide a better fit of the data it would not be physically consistent, since, for pure dust, if one specie was zero then all would have to be zero and a non-zero intercept regression violates that principle. These elemental ratios align well with reported values for dust transported to Europe[41-45] and Sahara source estimates[43], acknowledging variability in elemental composition depending on the emission area, particularly for Calcium. Despite this variability, given the extensive spatial and temporal coverage of our dataset across urban and rural environments, these ratios provide the the most comprehensive observational characterization of the average elemental composition of transported dust in Europe to date[46].

Line 149: "for local sources of dust such as windblown soil erosion, resuspension or agricultural activities" a reference is required here

We now cite the following three studies in the updated version of the manuscript.

Gillette, D. A. (1988). "Threshold Friction Velocities for Dust Production for Agricultural Soils." Journal of Geophysical Research-Atmospheres **93**(D10): 12645-12662.

Marticorena, B. and G. Bergametti (1995). "Modeling the Atmospheric Dust Cycle .1. Design of a Soil-Derived Dust Emission Scheme." Journal of Geophysical Research-Atmospheres 100(D8): 16415-16430.

Leung, D. M., Kok, J. F., Li, L., Okin, G. S., Prigent, C., Klose, M., Pérez García-Pando, C., Menut, L., Mahowald, N. M., Lawrence, D. M., and Chamecki, M.: A new process-based and scale-aware desert dust emission scheme for global climate models – Part I: Description and evaluation against inverse modeling emissions, Atmos. Chem. Phys., 23, 6487–6523, https://doi.org/10.5194/acp-23-6487-2023, 2023.

Line 168: "slightly overestimating concentrations below 0.1 μg m" and slightly underestimating" here it is necessary to be more quantitative

In line with a comment from another reviewer, the addition of Fig. S3 and pertinent statistics on Fig. S2 we have reformulated the statement in lines 198-203 to read:

The Random Forest (RF) model effectively captures daily dust concentrations, yet overestimates concentrations below 0.1 µg m^{-3} and underestimates the highest concentrations above 10 µg m^{-3} (Fig. S2b), leading to an overall positive bias (fractional bias: 38.8%). When averaged annually, the model shows much better agreement with the measurements (fractional bias: 14.2%), making the model well-suited for long-term trend analysis (Fig. S3).

Lincs 177–178: "The RF model captures the plums path and magnitude of the most severe and heavily studied Saharan dust events that hit the Balkans in April 2019"" more important is also to evaluate the capacity to represent moderate and low dust events

The reviewer is correct in pointing this out – indeed the RF model does a good job of approximating moderate and low dust events (dust concentrations < 20 µg m^{-3}) as evident by the multiyear site comparison in the new Fig. S3 and Fig. S5. We now mention this explicitly in the text in lines 211-213

It outperforms DREAM by not only capturing high dust episodes, but also moderate and low dust events (dust concentrations < 20 µg m^{-3}) (Fig. S5).

Line 180: please be more quantitative in defining low and high, concentrations

We thank the reviewer for pointing this out; we have now updated the statement so that it is also in line with the previous comment as well. Lines 217 to 218 now read

By comparison, the physical DREAM model exhibits a strong negative bias at concentrations below 20 µg m^{-3} and a tenfold positive bias at concentrations above 50 µg m^{-3} (Fig. S2a)

Line 210: temporal scale averages for these values and their uncertainties or statistical fluctuation have to be provided

We now clarify they refer to yearly average and provide the necessary uncertainties.

Yearly dust concentrations are predictably higher in southern Europe than in the North by a factor of 2.5 (5.28±2.65 µg m^{-3} vs 2.09±1.05 µg m^{-3}) (Fig 3a).

Uncertainties have also been added to all appropriate numbers mentioned in the text and further information has been added to the methods under 'Uncertainty of elemental ratios and dust estimate':

The uncertainty σ_{mean} in modelled yearly mean dust concentrations $dust_{mean}$ (including exceedance and background concentrations) is estimated by accounting for two main components: (1) the uncertainty in the derived dust/Al ratio (RE_{ratio} = 0.377/13.5=2.7%), and (2) the relative root mean square error of the modelled annual mean dust concentrations

($RRMSE_{annual\ mean}$ = 0.45; Fig. S3), as determined through a leave-one-out performance analysis against chemically derived dust concentrations from in-situ measurements (Eq. 1).

$$\sigma_{mean} = \sqrt{(RRMSE_{annual\ mean} * dust_{mean})^2 + (RE_{ratio} * dust_{mean})^2} = \sqrt{(RRMSE_{annual\ mean})^2 + RE_{ra}^2} * dust_{mean} \quad (1)$$

The robustness of dust concentration trends over the 10-year study period was evaluated by bootstrapping the linear regression of annual dust concentration time series at each grid cell (100 bootstrap resamples). The reported trend t_{dust} represents the mean of the bootstrap-derived slopes, where grid cells where the interquartile range includes zero are masked in white (zero trends are assumed for regional means in such cases). The overall uncertainty $\sigma_{trend,conc}$ in the trend (Eq. 2) includes both uncertainty in the dust/Al ratio and the bootstrap-based variability in fitting the trend (RE_{boot}, domain mean relative uncertainty excluding gridcells with non-significant trends), while the precision in the yearly dust prediction is already accounted for by the latter (see Eq. 1):

$$\sigma_{trend,conc} = \sqrt{(RE_{ratio} * t_{dust})^2 + (RE_{boot} * t_{dust})^2} = \sqrt{(RE_{ratio})^2 + (RE_{boot})^2} * t_{dust} \quad (2)$$

with $\sqrt{(RE_{ratio})^2 + (RE_{boo}\ )^2}$ equal to 0.5, 0.23 & 0.4 for the trend in mean, exceedance and non-exceedance concentrations respectively.

Analogously, we estimate the uncertainty σ_{ed} of the number of exceedance days and related trends. We use counting statistics to describe the uncertainty of the number of days ($RE_d =$ 0.17 , being the domain mean relative uncertainty based on $\sqrt{days}/days$) (Eq.3).

$$\sigma_{ed} = RE_d * days \quad (3)$$

The domain mean relative uncertainty $\sigma_{trend,day}$ (excluding gridcells with non-significant trends), in the trend of exceedance days t_{days} is estimated via the bootstrap-based relative

variability (RE_{db}=0.27) in fitting the trend (Eq. 4), which also accounts for the precision in the yearly prediction in exceedance days (see Eq. 3).

$$\sigma_{trend,day} = RE_{db} * t_{day} \quad (4)$$

Lines 212–214: it is not clear how a single episode in 2019 can relate to a 9-years statistics

The initial consideration was to suggest that the observed dust increases follow the Mediterranean transport pathway as did the 2019 episode. The current statement however is unclear and therefore we removed it.

Lines 215-216: consideration or discussion possible about the possible contribution of dust from high latitude regions, ex Iceland, in dust concentration in Northam Europe should be added

We thank the reviewer for mentioning this. The DREAM model used as input in the present study does include Icelandic dust, so a part of modelled dust in Northern Europe could potentially be coming from Iceland rather than the North Africa. We now mention it explicitly in the manuscript in lines 256 to 260:

A potential consideration for Northern Europe, is not only transported dust from the South, but also Icelandic dust. Iceland is the most important dust source for the high latitude areas of Europe, and also included in the model through DREAM[69], and therefore can contribute to the observed changes in concentration in Northern Europe in addition to North African dust.

Line 255: what is related or causes the background level increase should be discussed.

We thank the reviewer for raising this point. Compared to the clearer drivers behind the increase in dust concentrations during exceedance events in the North, the reasons for the rise in background dust concentrations are less well understood. However, the fact that this increase is observed both over land and sea suggests that it is more likely driven by enhanced long-range transport of dust rather than by local dust emissions or resuspension. In accordance with the suggestion from another reviewer we also conducted additional tests to gauge the importance of soil parameters in driving ambient transported dust concentrations (Fig. S10): These additional predictors did not improve the model performance and were not important predictors based on a SHAP analysis.

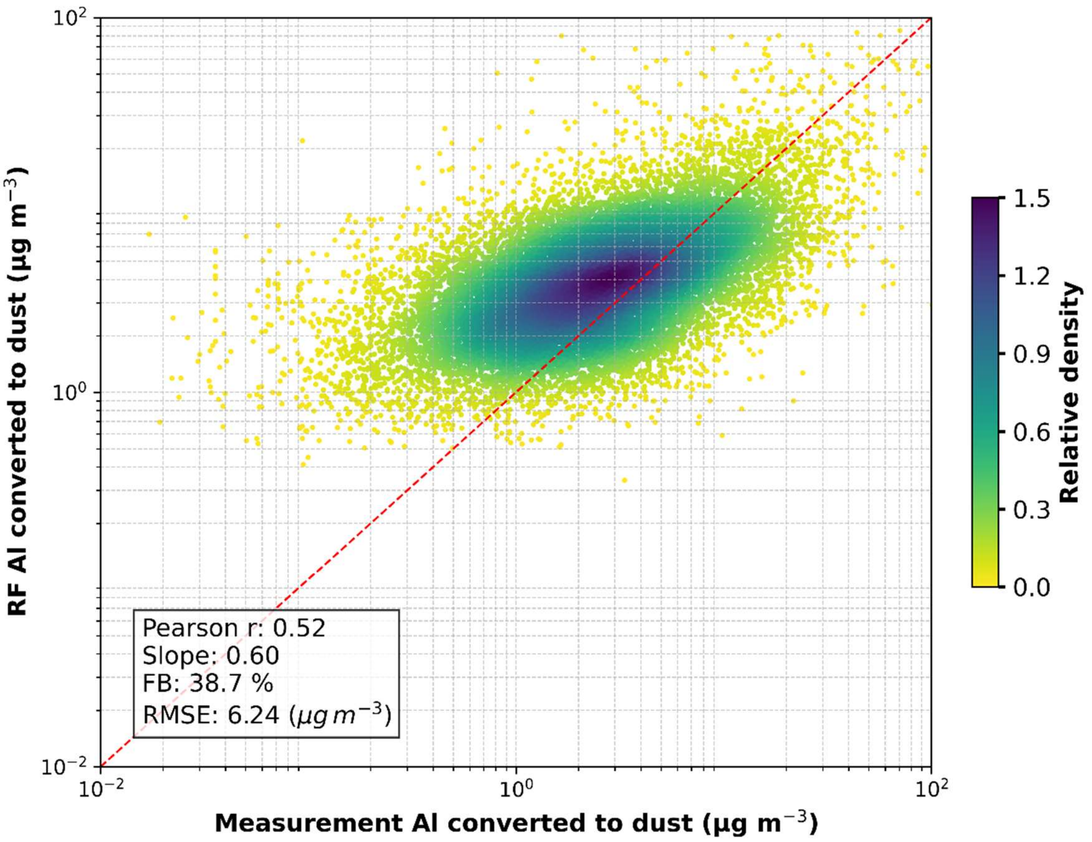

Fig S10 | Model training results when all available soil variables from the European Soil Database (https://esdac.jrc.ec.europa.eu/content/european-soil-database-v2-raster-library-1kmx1km) are added, including soil moisture & erodibility. Also shown are relevant statistical metrics (Pearson correlation coefficient r, linear regression slope, fractional bias FB & root mean square error RMSE). Results are shown in a logarithmic scale and the color represents the relative density of measurements.

We have updated the text accordingly in lines 301 to 307 to discuss potential drivers of the background increases

It should be noted that due to the relative nature of the exceedance metric, the background also includes dust events of concentrations below the threshold, and therefore it is expected that the observed increase is still attributed to transport. This interpretation is further supported by the fact that the increase during background conditions is observed over both land and sea —consistent with the spatial footprint expected for transported dust. Another potential driver is decreasing soil moisture due to warming, albeit the model predicted Al appears insensitive to perturbations of soil parameters (Fig. S10)

Line 269: reference number 61 cited at the end of this sentence refers to analysis of 2000-2007 period so it is not directly comparable with the 2012-2021 period

We thank the reviewer for mentioning this; while not directly comparable, the reference is valuable given that comprehensive studies investigating similar occurrences are scarce. We now acknowledge that it is not directly comparable to our study to avoid confusion. The text has been updated accordingly in lines 323 to 325

Similar trends have been observed in the past with satellite data for the period 2000 to 2007, with increased intrusion severity and slightly reduced frequency[70].

J.F. Kok, A scaling theory for the size distribution of emitted dust aerosols suggests climate models underestimate the size of the global dust cycle, Proc. Natl. Acad. Sci. U.S.A. 108 (3) 1016-1021, https://doi.org/10.1073/pnas.1014798108 (2011).

Referee #5 (Remarks to the Author):

The manuscript entitled "Rising Dust Pollution Threatens European Air Quality in a Changing Climate" synthesizes multi-source data to develop a random forest (RF) model characterizing dust events across Europe. It further investigates the underlying mechanisms and potential impacts of dust uplift. While the findings provide valuable insights that could inform dust monitoring and mitigation strategies, several critical scientific gaps and methodological uncertainties remain. These issues must be adequately addressed before the study can be considered for publication in Nature.

We thank the reviewer for their input and consideration. We conducted additional analyses to assess uncertainties and improve the quality of the manuscript based on the reviewer's comments.

1. Studies on the impact of climate change and dust on Europe has been relatively thorough, and similar conclusions are also quite abundant. The author needs to clarify whether the innovation of this study lies in the methodology or in the cognition. And highlight the key points where this study differs from or improves upon previous research. Such as: Shao, Y., M. Klose, and K.-H. Wyrwoll (2013), Recent global dust trend and connections to climate forcing, J. Geophys. Res. Atmos., 118, 11,107–11,118, doi:10.1002/jgrd.50836.

Vautard, R., Bessagnet, B., Chin, M., and Menut, L. (2005). On the contribution of natural Aeolian sources to particulate matter concentrations in Europe: Testing hypotheses with a modelling approach. Atmos. Environ. 39, 3291-3303. 10.1016/j.atmosenv.2005.01.051.

Li, Tiantian et al (2025). Sand and dust storms: a growing global health threat calls for international health studies to support policy action, The Lancet Planetary Health, Volume 9, Issue 1, e34 - e40.

Ying Dai, Peter Hitchcock, Natalie M. Mahowald,et.al. Stratospheric impacts on dust transport and air pollution in West Africa and the Eastern Mediterranean. Nature Communications, 2022.7744

Clifford, H. M., Spaulding, N. E., Kurbatov, A. V., More, A., Korotkikh, E. V., Sneed, S. B., et al. (2019). A 2000 year Saharan dust event proxy record from an ice core in the European Alps. Journal of Geophysical Research: Atmospheres, 124, https://doi.org/10.1029/2019JD030725

We thank the reviewer for highlighting the need to better emphasize the innovation of our study. While several previous works (e.g., Shao et al., 2013; Vautard et al., 2005; Li et al., 2025; Dai et al., 2022) have examined the links between dust, climate, and air quality, our study advances this field both methodologically and conceptually. Methodologically, we integrate the most extensive multi-year elemental dataset in Europe with satellite-based dust optical depth, land use, meteorology and outputs from a state-of-the-art physical dust model within a Random Forest

framework—yielding substantially improved performance over state-of-the-art chemical transport models and allowing a robust analysis of dust phenomenology (spatially resolved concentrations and temporal trends). Conceptually, our approach bridges the short-term climatology of dust, dominated by meteorology (NAO, SaOI, and MOI) with long-term variability inferred from ice-core records, revealing a consistent link between North African dryness and dust emissions. This comprehensive, data-driven framework provides the most spatially and temporally resolved assessment of transported dust in Europe to date and robust assessments of its implications for air quality and health.

Based on the reviewer's comment we have adapted the introduction, with references to previous publications, including those mentioned by the reviewer. The updated introductory text reads as follows from lines 84 to 117:

Mineral desert dust is a major contributor to total atmospheric particulate matter (PM) smaller than 10 µm (PM_{10}), with its influence expected to grow due to climate change[1-3] and the decrease in anthropogenic pollution due to air quality mitigation and Net Zero strategies in many regions[4]. Dust plays a central role in the Earth and Climate systems by interacting with solar and terrestrial infrared-radiation[5,6], serving as a cloud condensation and ice nuclei[7-10], supplying iron and other nutrients to ecosystems[11-13] – particularly over oceans – and influencing atmospheric acidity[11,14-16], which affects the partitioning of inorganic and organic vapors[17]. Desert dust outbreaks degrade air quality by raising PM_{10} and $PM_{2.5}$ (particulate matter (PM) smaller than 2.5 µm) levels[18,19], disrupt economic activities such as air traffic[20-24] and pose adverse health effects[25], including asthma exacerbation[26-28], stillbirths[29], increased mortality[30], and even the transport of pathogens[31]. Analyses of transported dust outbreaks from the Saharan and Middle East deserts, at specific European locations reveal a rising intensity and frequency in recent decades[3,32-35]. Regional and global modelling show that dust emissions and transport are tightly coupled to large-scale circulation patterns, including the North Atlantic Oscillation[36,37] (NAO) and stratospheric intrusions[35,38], which would modulate the frequency of dust intrusions over Europe. Long-term reconstructions from Alpine ice cores[39] and recent assessments of dust-related health risks[25] further highlight the growing significance of dust under a changing climate, likely linked to increasing North African droughts. However, due to the strong spatial and temporal variability in dust levels, it remains unclear whether this rise is consistent across Europe and whether desertification and aridity or shifts in atmospheric circulation are the main drivers behind this rise. Most existing studies rely on coarse resolution dust transport models, satellite proxies or total PM10 concentrations[40,41] to infer dust concentrations, approaches limited by uncertainties in emission parameterizations, spatial resolution, or the lack of observational constraints. Consequently, there is no continent-wide, observation-driven quantification of dust levels, trends, drivers and impacts on air quality and health.

Here, we address this gap by developing a data-driven, observation-constrained Random Forest (RF) model trained on the most extensive elemental dataset available in Europe. The model integrates multiple data products, including satellite-derived dust optical depth, land use information, and a state-of-the-art physical dust model. The resulting European-wide

daily dust PM10 concentrations for 2012–2021, combined with ice-core observations, enable a comprehensive characterization of both the short-term variability and long-term drivers of dust, identifying the climatic modes controlling its transport and assessing its contribution to current air quality limit values and associated mortality from short-term exposure.

2. The study exhibits significant methodological gaps, as it lacks several critical auxiliary analyses to substantiate its conclusions. Key supporting evidence—such as quantitative simulations of dust emission intensity in the Sahara Desert, trajectory modeling of European air currents, and satellite remote sensing data—is either missing or insufficiently utilized. For example, while the authors attribute the rise in European dust levels to Sahara Desertification, this claim would be substantially strengthened by robust satellite-based evidence, which currently appears underdeveloped in the analysis.

We thank the reviewer for this comment. While our study does not perform independent physical modeling of dust emissions, it fully incorporates key quantitative information on dust source intensity and transport as inputs to the Random Forest (RF) model. Specifically, satellite remote sensing data are used to generate daily dust optical depth fields at 550 nm, while the DREAM model provides physically based simulations of Saharan dust emissions and transport pathways throughout Europe. These datasets ensure that the RF model is informed by both source-level emissions and realistic atmospheric transport. Information about the model inputs used are now provided in the new Table S3 and in the "Model inputs" Methods section.

Table S3 | Model variables used in this study.

Variable	Resolution (km)	Type	Source
**10-meter wind speed**	5.5	reanalysis	CERRA sub-daily regional reanalysis
**10-meter wind direction**	5.5	reanalysis	CERRA sub-daily regional reanalysis
**2-meter temperature**	5.5	reanalysis	CERRA sub-daily regional reanalysis
**total precipitation**	5.5	reanalysis	CERRA sub-daily regional reanalysis
**Dust aerosol optical depth at 550 nm (duaod550)**	83.325	reanalysis	CAMS global reanalysis (EAC4)
**DREAM**	36.663	forecast	WMO Barcelona Dust Regional Center

The RF model has been extensively validated against ground-based measurements across Europe and against ice-core records, and it outperforms both the DREAM model and optical depth reanalysis in reproducing observed dust concentrations. Taken together, the use of satellite and physical model inputs, combined with rigorous validation, supports the reliability of our RF-based reconstructions for analyzing dust levels, variability, and drivers across Europe.

3. Data Harmonization and Comparability: A critical methodological concern pertains to the comparability of chemical composition data across multiple sampling sites. While the manuscript briefly acknowledges this issue (Lines 363-365), stating that 'great care was taken in curating the database by flagging data below detection limits and working with data providers to ensure high data quality,' this explanation remains insufficiently detailed. Specifically, the following critical questions remain unanswered: (1) What systematic approaches were implemented to address measurement discrepancies arising from heterogeneous analytical techniques? (2) What specific calibration protocols, normalization procedures, or data harmonization methods were employed to ensure cross-site comparability? (3) Were any quality control metrics or uncertainty estimates applied to account for potential inter-instrument variability?

This is a valuable comment and the presents the opportunity to clarify the data harmonization and comparability aspects of our analysis.

Previous intercomparisons of elemental measurement techniques. **All elemental data used in this work were obtained from established and widely adopted analytical methods in atmospheric aerosol research, namely X-ray Fluorescence (XRF, including the Xact instrument), Particle-Induced X-ray Emission (PIXE), and Inductively Coupled Plasma Mass Spectrometry (ICP). Numerous inter-laboratory comparison studies have shown that these techniques yield highly consistent results for particulate elemental concentrations, including aluminum (Al). For example, Gini et al. (2021) demonstrated excellent agreement (<10% difference) between PIXE and XRF for Al in PM10 samples, while Chiari et al. (2018) and Lucarelli et al. (2011) also reported strong correlations between these methods. Similarly, comparisons between ICP and PIXE/XRF (Saitoh et al., 2002) show $R^2 > 0.9$ for Al, confirming their cross-method reliability. Based on this body of literature, we consider Al measurements from these techniques directly comparable for the purposes of trend analysis.**

Data harmonization and quality control. **As detailed in the revised Methods section, harmonization and quality control procedures were applied after aggregating all datasets. Specifically, we removed values below the detection limit (Al < 0.003 μg m^{-3}), filtered out repeated or rounded data likely representing detection limits, excluded records with time resolutions >1 day, and aggregated hourly data to daily means. Xact-derived Al data were reconstructed using Ti concentrations and Ti/Al ratios derived from the harmonized database to correct known biases.**

Assessment of potential biases arising from heterogeneous analytical techniques. **As we have noted in the method section, we have worked with data providers to collect their**

quality ensured data, including metadata. The sampling sites and analysis techniques were not selected during this study but designed by the data providers for their specific cases and needs. This ensured the collection of a large, multi-site, multi-year dataset. Regarding the specific 3 questions raised by the reviewer, we confirm that no additional calibration, normalization, or harmonization procedures were applied beyond the quality assurance and calibration originally performed by the data providers. Each dataset was used as delivered, following the providers' internal QA/QC protocols. Accordingly, no inter-instrument correction factors were introduced, except for the harmonization steps now described in the updated Methods section (Lines 452–478), which primarily concern detection limit treatment, time resolution filtering, and reconstruction of Xact-derived Al data. Therefore, the dataset represents authentic, provider-level quality-assured measurements.

To further address the reviewer's concern, we have now explicitly evaluated the consistency of results across measurement techniques. In the revised Supplementary Information (Fig. S12), we present comparisons of measured versus RF-modeled dust concentrations, grouped by analytical method (XRF, PIXE, ICP). The coherent model performance across all methods indicates no significant systematic biases due to heterogeneity in analytical techniques.

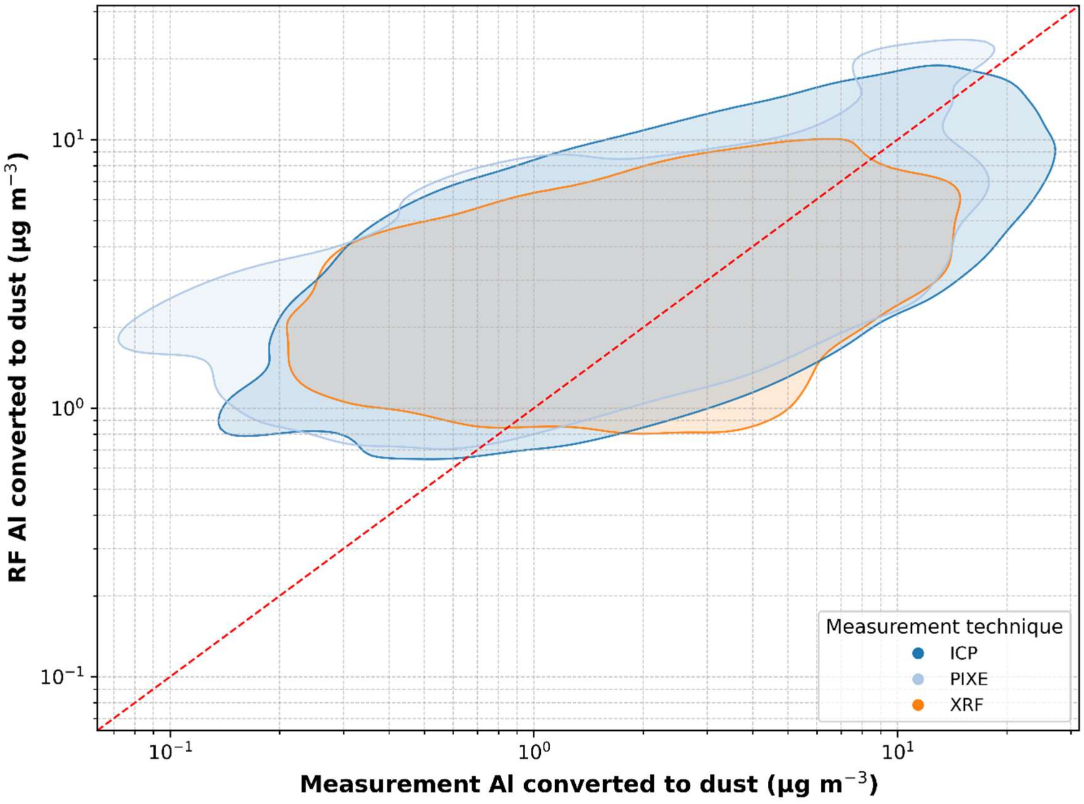

Fig S12 | Density plot for 90% of predictions, grouped by technique, for the leave-1-out results.

Overall, these steps—combined with the demonstrated inter-method comparability in the literature—support the robustness of the harmonized dataset used in this study.

We have now amended the "Dataset and database construction" in the Method section as follows to reflect this response:

Dataset and database construction

We compiled an extensive database of daily average measurements of PM_{10} elements related to dust (Al, Ti, Si, Ca, Fe) from all over Europe, to estimate dust concentrations. The bulk of the data was compiled ad-hoc for this study, while some was extracted from the EBAS database[86]. The most frequently measured metal that is prevalent in mineral dust was aluminium; measurement data for other dust metals found purely in dust such as silicon and titanium were much scarcer. In contrast, local sources such as road dust as well as non-exhaust traffic and construction emissions, affect calcium and iron, rendering them unsuitable for estimating transported dust in this study[66]. Metal concentrations were measured both with offline filter-based techniques (ICP, PIXE & offline XRF)[87] and online techniques (online XRF XACT)[88,89]. Al measurements from these techniques are directly comparable for the purposes of trend analysis, since numerous inter-laboratory comparison studies have shown that these techniques yield highly consistent results for particulate elemental concentrations[90-94]. In addition, grouping model random forest cross validation results by training technique (XRF-based, ICP-based or PIXE – Fig. S12), does not indicate any inherent biases between the techniques. Great care was taken in curating the database by flagging data below detection limits and working with data providers to ensure high data quality. After data were aggregated from all providers various methods were employed to ensure data quality. Low concentration values at the detection limit were discarded. To that end, concentration values below a detection cutoff of Al = 0.003 $\mu g\ m^{-3}$ were removed. In addition, values that were repeated more than 3 times in sequence or repeated more than 5 times (at a third decimal precision) were interpreted as data that was replaced by the detection limit. Further data with a time resolution > 1 day were discarded, while hourly data were aggregated to daily averages.

4. Scientific Rationale and Ratio Interpretation: Several aspects of the study design need further elaboration. (1) Why was aluminum (Al) selected as the target variable for dust estimation? How were other crustal elements, such as silicon (Si) and iron (Fe)? (2) The reliability of key elemental ratios—such as Si/Al, Fe/Al, Ca/Al, and Ti/Al—is questionable. It is striking that these ratios appear to fall within such narrow and consistent ranges across numerous stations and time periods. Furthermore, these ratios lack contextual comparison with source-region dust compositions. Although the authors cite Chiapello et al., certain ratios, such as Fe/Al and Ca/Al, differ substantially from those reported in that study. A more rigorous evaluation of the stability and representativeness of these ratios is required to validate the study's conclusions.

We thank the reviewer for this insightful comment. Aluminum (Al) was selected as the primary tracer because it is the most consistently measured crustal element across European monitoring networks. While silicon (Si) would in principle be an ideal tracer for transported dust, Si measurements are considerably sparser due

to the interference from quartz filter material. Importantly, in sites where both Si and Al are available, the two correlate strongly (Fig. E1), confirming that Al is a robust proxy for mineral dust in our dataset.

Other crustal elements, such as calcium (Ca) and iron (Fe), were not chosen as primary tracers due to their potentially strong local contributions. To further minimize local influence in the determination of elemental ratios, we restricted our ratio calculations to days with Al concentrations above 1 µg m^{-3}, representing transported dust conditions.

Regarding the elemental ratios (Si/Al, Fe/Al, Ca/Al, Ti/Al), we agree that assessing their stability and representativeness is essential. We would like to clarify that the violin plots in Figure 1 (now updated) represent the confidence intervals for *average ratios* obtained through a bootstrapping approach, and not the site-to-site and day-to-day data variability. We added a new supplementary figure (Fig. S13) showing additional sensitivity tests. Moreover, Figure 1 has been updated to include a detailed comparison with previously reported values, demonstrating that the average ratios determined in our study are within the range of literature values.

The revised text in the main manuscript (Lines 185-190 and 142–147) now explicitly explains the rationale for selecting Al, the treatment of ratio variability, and the comparison with existing studies. It reads as follows:

[…] For quantifying dust, we opted for a single-tracer approach rather than a multi-tracer one, as most locations lack complete tracer data, making it impossible to train the random forest model consistently. While silicon (Si) would be an ideal tracer due to its clear association with transported dust, Si measurements are notably less abundant than those of aluminum (Al). Given the strong correlation between Al and Si where both are available, we rely on Al as a transported dust proxy in the RF model (Fig. E1). […]

[…] These elemental ratios align well with reported values for dust transported to Europe[41-45] and North Africa source estimates[43], acknowledging variability in elemental composition depending on the emission area, particularly for Calcium. Despite this variability, given the extensive spatial and temporal coverage of our dataset across urban and rural environments, these ratios provide the most robust characterization of the average elemental composition of transported dust in Europe to date[46]. […]

The updated Figure 1 and its caption are pasted below.

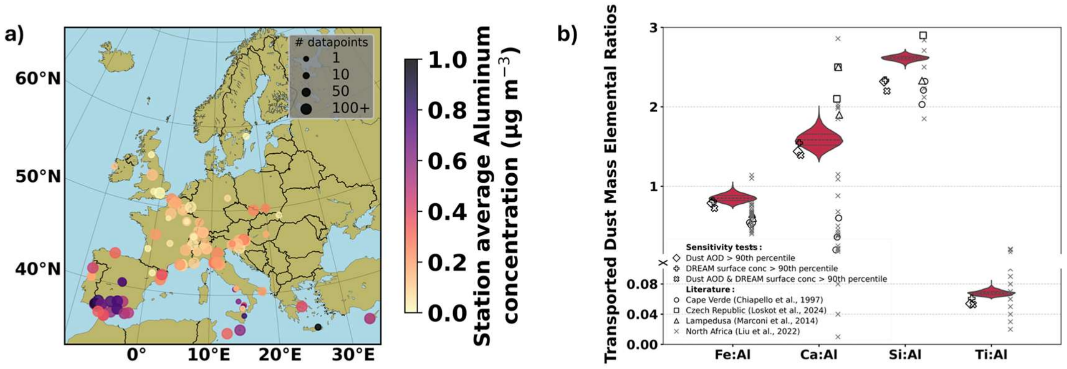

Fig 7 | Dust observations and elemental ratios of transported dust in Europe. a) average aluminium concentrations for 103 locations in Europe. The size of the dot corresponds to the number of daily measurements, while the color to the average concentration of *Al* at this location. High *Al* concentrations show the large impact of transported dust on air quality in Southern Europe. b) Violin plots of elemental ratios of transported dust, estimated via bootstrapped zero-intercept linear regressions applied to daily data with elevated transported dust concentrations (aluminium concentrations >1 µg m^{-3} for Fe:Al and Ca:Al). The diamonds, crosses and x's correspond to filtering to the 90th percentile of dust optical depth, DREAM dust surface concentration and both. Circles, squares and triangles represent literature values from Chiapello et al 1997[46], Loskot et al. 2024[48], Marconi et al. 2014[47] for dust arriving to Europe, while North Africa source estimates from Liu et al., 2022[44] are denoted with the gray x's

The new Figure S13 is presented below.

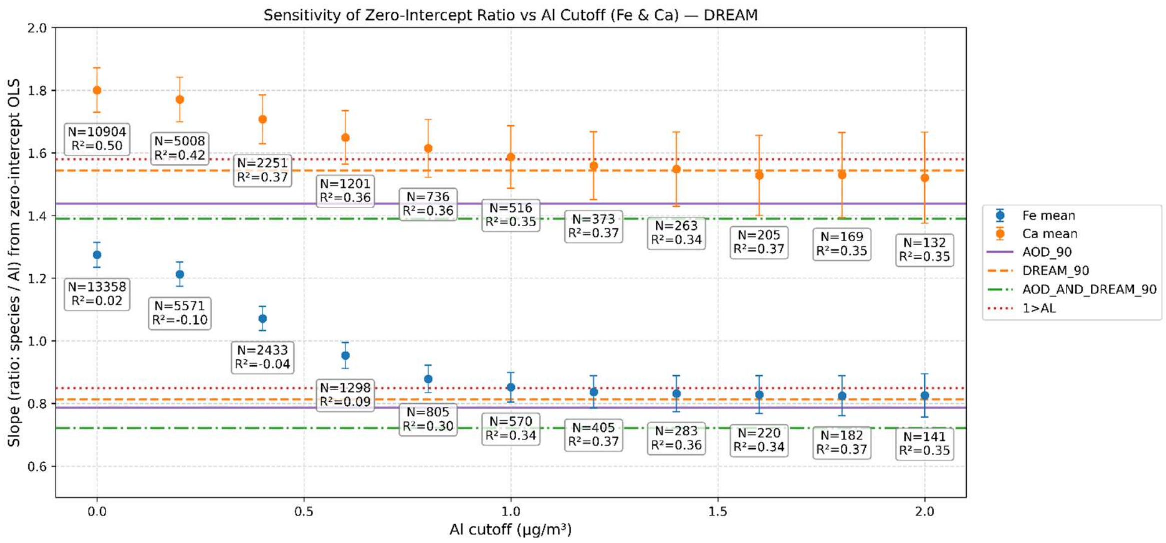

Fig S13 | Sensitivity of the mass-based elemental ratios of Fe/Al & Ca/Al for different Al cutoffs. The ratios for the cases where Al>1μg m^{-3}, and for the 90th percentile of DREAM surface dust concentration, dust AOD and both at the same time are shown as horizontal lines.

5. This study aims to reflect the impact of dust events, using the RF model to simulate Al concentrations, and further calculate dust concentrations. Why not directly use the monitored Al concentration? What is the significance of using the RF model to re-estimate the Al concentration, just for spatial analysis? The author also compared their modeled concentrations with monitoring.

This is an important comment. Directly using monitored Al concentrations is extremely prohibitive and would not allow us to capture spatial and temporal trends and patterns, as elemental measurements are often sparse—both in time (typically every few days or limited to specific months) and space. For continental scales as the ones presented in this work, physical modeling approaches have been generally used to provide spatial information. However, resulting concentration fields tend to be significantly less accurate than measured concentrations.

To overcome these limitations, we developed a data-driven Random Forest (RF) model trained on the available Al observations, while integrating physically based predictors such as AOD reanalysis, meteorology, land-use information, and outputs from a state-of-the-art dust model. This hybrid approach leverages the strengths of both measurements and modeling: it preserves the accuracy of observations while providing complete, high-resolution spatiotemporal fields of dust concentrations (Fig. S2).

We compare RF-modelling outputs to measurements for model validation using a leave-one-out-station cross-validation approach, demonstrating robust agreement with independent measurements.

Thus, the RF model is indispensable for quantifying continental-scale dust variability, identifying drivers of dust intrusions (Fig. 4), and reconstructing long-term spatiotemporal trends (Figs. 2–3) that could not be resolved from observations alone.

6. Model Evaluation and Uncertainty: The reliability and predictive accuracy of the RF model need to be more thoroughly demonstrated. Currently, the manuscript lacks quantified performance metrics (e.g., R^2, RMSE, MAE) in the "Model Performance" section. Moreover, Figure S4 suggests notable discrepancies between observed and modeled Al concentrations, particularly a tendency for the model to underestimate high values and overestimate low values. This pattern is especially evident during extreme dust

episodes, which the model appears unable to capture effectively. These limitations should be explicitly discussed.

We thank the reviewer for this valuable comment and for highlighting the need for explicit model performance metrics. We have now included quantitative evaluation parameters—r, slope of the fit, fractional bias, and RMSE—on the leave-one-station-out cross-validation results for both the RF and the DREAM models (Fig. S2).

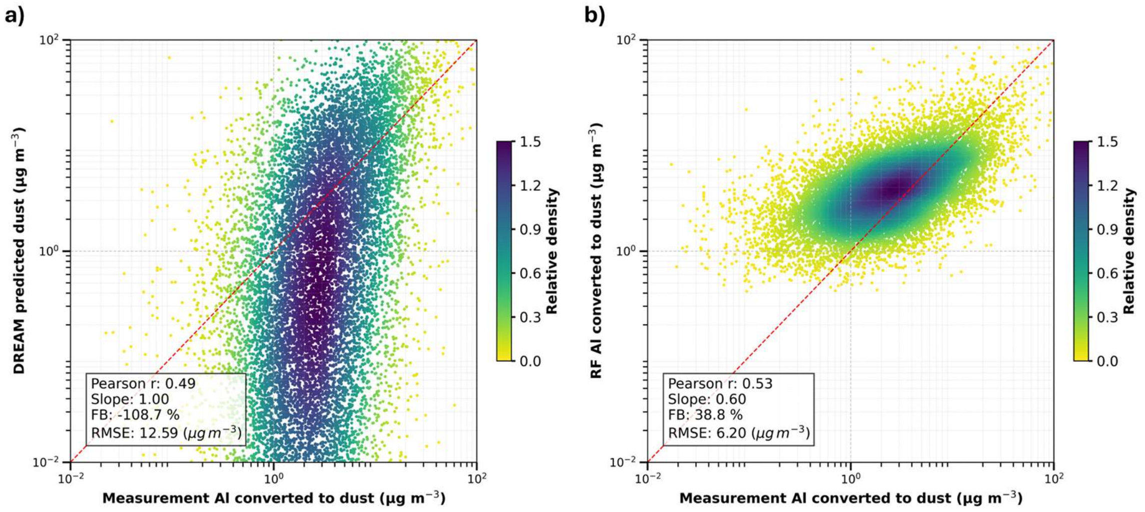

Fig S2 | Comparison between measured dust vs. (a) DREAM predicted dust, and (b) Random Forest (RF) predicted dust (b), along with relevant statistical metrics (Pearson correlation coefficient r, linear regression slope, fractional bias FB & root mean square error RMSE). Results are shown in a logarithmic scale and the color represents the relative density of measurements.

The reviewer is correct that the RF model shows a tendency to underestimate very high Al daily concentrations and overestimate low daily values, a pattern already noted in the manuscript. Nevertheless, the RF model represents a substantial improvement over the DREAM outputs, particularly in capturing the observed variability and overall concentration levels (see new Fig. S2). We have further demonstrated that the RF model accurately reproduces both the timing and magnitude of a well-documented dust event (lines 213-216 Fig. S7) and resolves most major dust episodes at long-term monitoring sites (Fig. S5).

To assess the model's robustness in representing long-term averages—the basis of several key conclusions—we evaluated its performance against annual mean concentrations. The RF predictions show a slope of 1, suggesting that our modeled dust levels are accurate (Fig. S3).

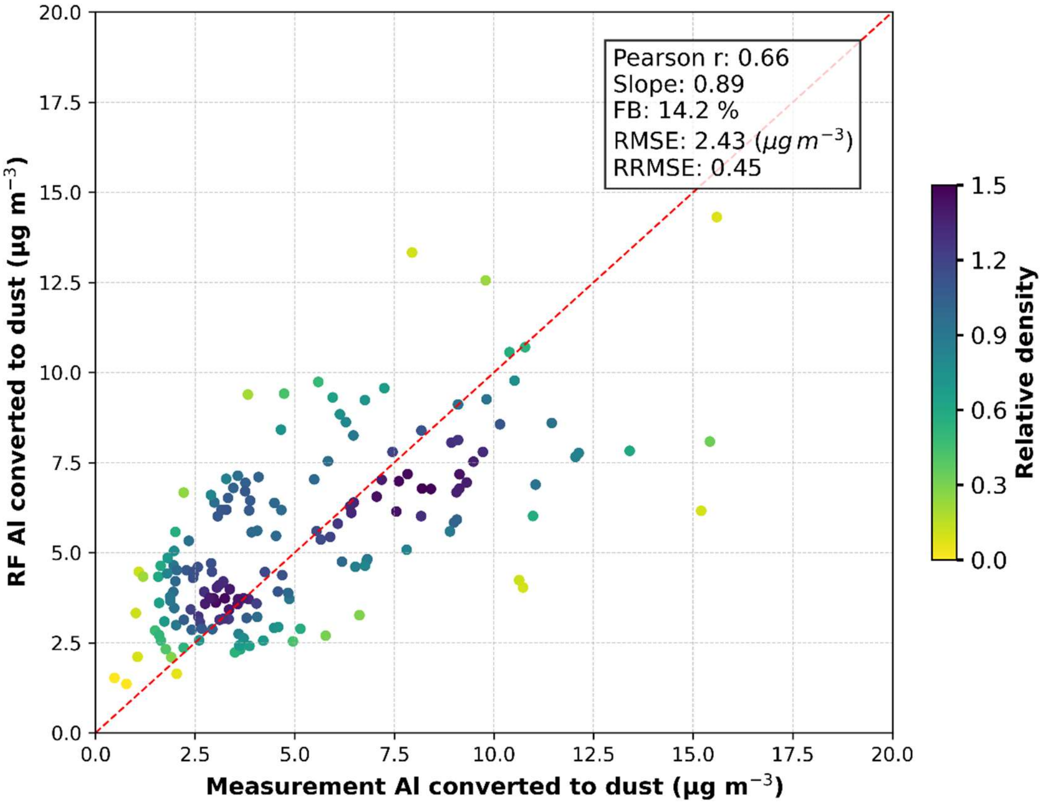

Fig S3 | Leave-1-out results aggregated on a yearly basis, with relevant statistical metrics (Pearson correlation coefficient r, linear regression slope, fractional bias FB & root mean square error RMSE).

We have expanded the discussion of model performance and uncertainty in the Methods of the revised manuscript to emphasize these points and to clarify both the strengths and limitations of the RF approach. The updated text reads as follows (lines 480-522):

Uncertainty of elemental ratios and dust estimate

We quantified the impact of uncertainty in the elemental ratios—defined here as one standard deviation from the bootstrap results shown in Fig. 1b (Si:Al 2.61±0.033, Ti:Al , 0.068±0.003, , Ca:Al 1.58±0.099, and , Fe:Al 0.89±0.052)—on the dust estimates using uncertainty propagation based on the formula: 2.2Al + 2.49Si + 1.63Ca + 1.94Ti + 2.42Fe. This results in a total dust estimate of (13.5 ± 0.377) × Al. Notably, the largest contributions to the overall uncertainty stem from Fe and Ca, which are also influenced by substantial local sources. Zero-intercept fits were used for element–element plots (e.g., Ca concentration vs. Al concentration) to estimate dust-phase mass-based elemental ratios under the assumption that—after blank correction and removal of non-dust contributions—both analytes originate from the same dust endmember. In that case, when the dust contribution vanishes (Al → 0), the co-emitted element should also vanish (Ca → 0),

implying a physically meaningful intercept of zero. In addition, we tested a linear model with an intercept using bootstrap analysis and found that zero lies within one standard deviation of the mean intercept (intercept ± $\sigma_{intercept}$), supporting the use of a zero-intercept model. Given this result, along with the physical consistency and practical advantages of a zero-intercept regression—such as enabling the use of average ratios to estimate other components—we opted for the zero-intercept model. A summary table of the relevant statistics is provided in the Supplement (Table S2).

Elemental ratios on days with low amounts of transported dust can be substantially affected by local sources. With a rather high Aluminium concentration threshold of >1 ug/m3 for determining the transported dust elemental ratios, we aimed at minimizing the impact of local sources on the elemental ratios. In addition, we performed a sensitivity analysis varying the threshold showing that the elemental ratios vary with cutoffs above 0.5 for Ca:Al and 0.75 for Fe:Al within the 1 standard deviation of the value obtained with a cutoff of 1 ug/m3. Further, we investigated model-driven criteria for dust episodes instead of using a static Aluminium concentration cutoff using the DREAM and optical depth values and only including data points that were higher than the 90th percentile in those (each alone or both at the same time), as a proxy for dust events. This approach resulted in similar values (Fig. 1b & S13).

7. The use of DREAM-modeled dust as an input variable raises methodological concerns. Given that DREAM is driven by meteorological factors, and these same meteorological variables are also directly input into the RF model, there is a risk of information redundancy or collinearity. How did the authors address potential overlaps between direct and indirect representations of meteorological drivers?

We thank the reviewer for raising this important point. The potential overlap between meteorological variables and the DREAM-modeled dust fields is a valid concern. In our framework, DREAM dust was used as a physically based indicator of both large-scale dust transport and emission potential—information that is complementary to local meteorological predictors. In the Figure below (not included in the manuscript), we analyze the correlation between DREAM outputs and individual local meteorological parameters showing weak to no correlations.

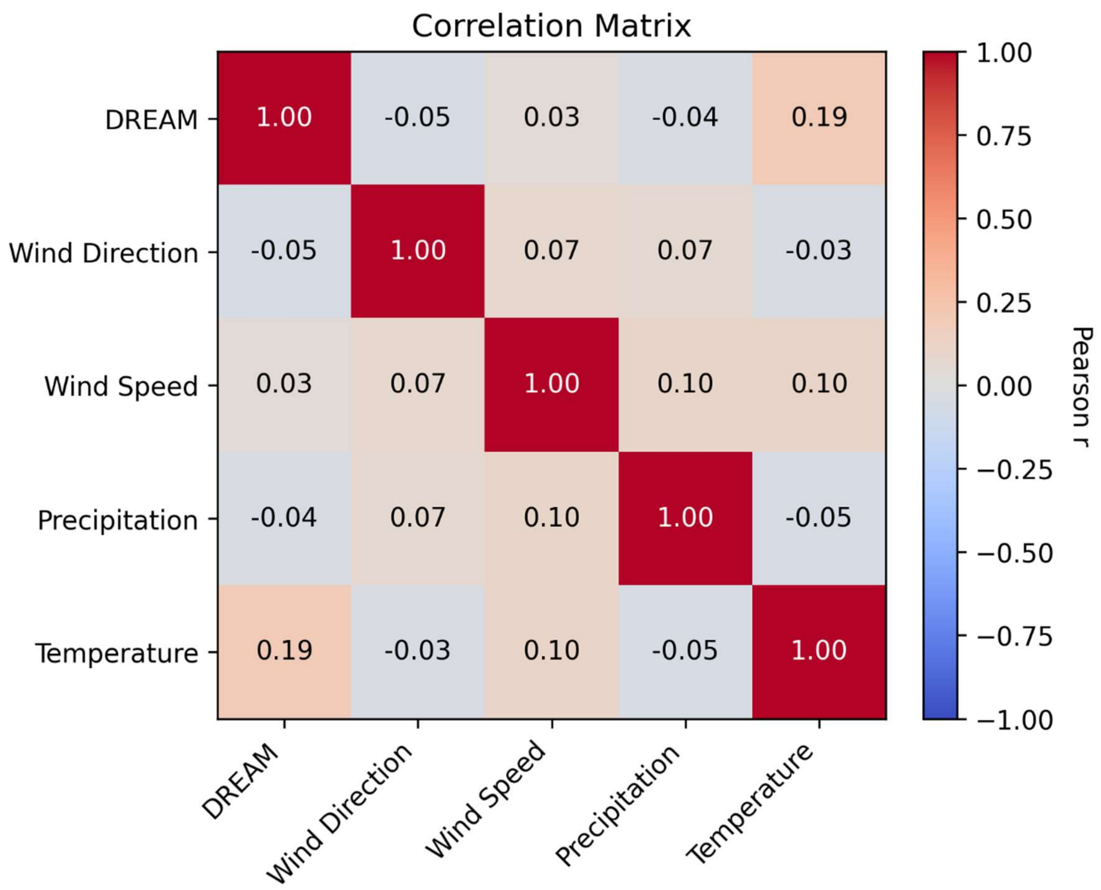

While some degree of correlation between these inputs may still exist, the RF algorithm is inherently robust to multicollinearity because it is a non-parametric ensemble method that randomly samples both features and data subsets during training. This limits the influence of correlated predictors and prevents model instability or overfitting.

We assessed potential redundancy between DREAM and meteorological predictors using SHAP and variable importance analyses (Fig S4). DREAM dust emerged as the most influential predictor (~30% of explained variance), while individual

meteorological variables contributed <5% each. This confirms that DREAM dust provides complementary, not overlapping, information—capturing emission and transport dynamics distinct from local meteorological effects.

We have updated the method section "Model inputs" of the new version of the manuscript as follows:

While RF is inherently resilient to potential collinearities[96], to evaluate potential redundancy between DREAM-modeled dust and meteorological predictors, we examined the contribution of each variable to the Random Forest model performance using both SHAP (Shapley Additive Explanations) values and variable importance metrics. DREAM dust emerged as the single most influential predictor, accounting for roughly 30% of the explained variance in model outputs, whereas individual meteorological variables (e.g., wind speed) contributed substantially less (<5% each). This dominance of DREAM dust in both metrics, combined with the distinct physical meaning of this variable — representing long-range dust emission and transport processes rather than local meteorology —demonstrates that it provides independent explanatory power rather than redundant information.

8. Using spatio-temporal correlation analysis to investigate climate change's driving mechanisms and impacts on European dust. However, this methodological approach faces substantial critiques regarding its capacity to authenticate causal relationships and credibly elucidate underlying physical processes.

We fully acknowledge that our analysis is correlation-based rather than a formal causal inference. However, correlation analyses are a well-established approach for identifying statistically robust linkages between climatic modes and pollution variability. In the revised manuscript, we have strengthened this analysis by retaining only statistically significant correlations in the Figures, ensuring robustness and avoiding misinterpretations. We have also carefully revised the text to avoid implying causality, while clearly emphasizing that the identified relationships represent strong and consistent statistical associations that provide valuable insights into the climatic drivers of dust variability over Europe. Changes to the manuscript read as follows:

Abstract: Intensified dust intrusions over the past decade are strongly linked to shifts in atmospheric circulation, while an ice-core record from the Swiss-Italian Alps reveals a 110% increase in dust concentrations since preindustrial times, most of it being associated with desertification in North Africa.

Results: To assess whether the observed decadal trends reflect a sustained long-term increase in dust concentrations, we analyzed Ca^{2+} concentration levels in Alpine ice cores from Colle Gnifetti (CG, Monte Rosa, 4450 m a.s.l., 45°55′46"N; 07°52′30"E) for the period 1750-2020. The dataset combines three cores - one drilled in 2021 and two parallel cores recovered in 2003[73-75]. Because Al concentration data were only available until 1990, Ca^{2+} was used as a proxy for dust, an appropriate choice given the strong correlation between Ca^{2+} and Al during the overlapping period ($r = 0.87$, 1750–1990) and the absence

of local sources at Colle Gnifetti. Although the ice core record reflects total dust deposition rather than atmospheric concentrations, it exhibits a consistent temporal pattern with the modeled dust concentrations over the nine overlapping years (2012–2020; inset Fig. 5a), including a slight decline over the past decade—consistent with modelled trends across the Alps (Fig. 3b). Over the long term, Ca^{2+} concentrations preserved in the ice archive increased by approximately 110% between the preindustrial period (1750–1850) and the past decade (2010–2020) (Fig. 5a). The trend derived from the three Colle Gnifetti cores is consistent with the multisite analysis of Kok et al. 2023[6], who report a similar but less pronounced increase over the 20th century. On interannual scales, Ca^{2+} concentrations in the ice core are correlated with the North Atlantic Oscillation (NAO; $R_s = 0.48$), consistent with the established influence of NAO-related circulation variability on dust transport[35,36,38,39,76]. In addition, the long-term trend in Ca^{2+} exhibits a strong negative correlation with the self-calibrating Palmer Drought Severity Index (PDSI)[77,78], which characterizes hydroclimatic variability over the primary dust source regions for the Swiss–Italian Alps, located in the northwestern Sahara (32.15°–35.66° N; −7.14°–11.27° E, following Coen et al. [79]). Drier conditions—corresponding to lower PDSI values—are associated with higher Ca^{2+} levels in the ice core ($R_s = -0.70$). When restricting the analysis to Moroccan dust sources, the correlation strengthens further ($R_s = -0.78$). These statistically robust associations indicate that long-term dust variability recorded in the Alpine archive is most strongly linked with persistent aridification trends in North Africa and with large-scale circulation variability, as represented by the NAO, which modulates precipitation and surface conditions over key dust source regions[37,80] (Fig. 5a,b).

In Figure 5d–f, we examine how circulation-oriented climate indices are statistically associated with dust inputs to Europe, alongside the dryness of the Sahara (Fig. 5c), considering a broad source region encompassing most of the Sahara (14.2°–30.17° N, −9.15°–32.52° E). The Saharan Oscillation Index (SaOI) represents the pressure difference between the Azores (37.79° N, −25.5° E) and Niamey (13.51° N, 2.10° E)[81], while the Mediterranean Oscillation Index (MOI)[82] corresponds to the normalized pressure difference between Algiers and Cairo. Both indices are closely related to the North Atlantic Oscillation (NAO)[81,82]. During 2012–2021, dust concentrations were strongly correlated with SaOI and anticorrelated with MOI (Fig. 5e,f), except in Western Europe, where correlations were weaker and occasionally of opposite sign. This pattern may reflect the dual influence of MOI: while low MOI values are associated with enhanced westward dust transport, they also coincide with higher precipitation over Western Europe[82], likely offsetting increases in surface dust concentrations through wet deposition. Increasing SaOI values were most strongly correlated with enhanced Saharan dust advection toward Europe, whereas declining MOI values were associated with reduced eastward transport and a relative strengthening of dust inflow to central and southern Europe. These combined circulation tendencies are most strongly linked to intensified southerly winds from North Africa, consistent with the spatial dust patterns shown in Figure 4d,f.

Similarly, the NAO exhibits a robust statistical relationship with dust transport. Positive NAO phases during 2012–2021 were associated with stronger cyclonic activity and westward transport, corresponding to higher dust levels over the Atlantic, France, and

Spain[66] (Fig. 5c), while stronger westerlies under these conditions coincided with reduced dust transport into central and eastern Europe. Over the Aegean Sea, elevated dust concentrations during positive NAO phases are most likely linked to intensified easterly winds in the far eastern Mediterranean[64].

Overall, the observed dust variability across Europe is most strongly correlated with concurrent trends in North African desertification and regional circulation patterns, emphasizing the dual influence of source-region aridity and large-scale atmospheric dynamics on dust transport toward Europe.

Added to Caption of Figure 5: This analysis is designed to reveal statistically significant associations, not causal relationships; however, the robustness of these correlations provides valuable insights into the drivers of the short-term variability in dust over Europe.

Referee #5 (Remarks to the Author):

The author has made substantial revisions and additions to the manuscript, addressing most concerns. However, several issues still require clarification, especially regarding modeling methods:

We thank the reviewer for their careful reassessment and for acknowledging the revisions made to the manuscript. In the revised version, we have further clarified the modeling methodology by expanding the Methods section and adding additional validation analyses. Below we address the remaining comments in detail.

1. Model Performance: The authors still do not directly provide key evaluation metrics for RF modeling. Given that r in Fig. S3 is 0.66, R^2 should be around 0.44. According to Fig. S2, the R^2 value at higher time resolutions is lower, and should be only around 0.28, which is not a particularly favorable indicator for machine learning. Furthermore, while the authors currently use leave-one-out cross-validation to assess the performance of the RF method at spatial sites, have they considered the generalization capability and performance evaluation of the RF model on time series data?

We thank the reviewer for their constructive comment regarding model evaluation and temporal generalization. First, we agree that key performance metrics should be reported more explicitly. To improve transparency, we have now added a table summarizing the main evaluation metrics for the RF model, including correlation coefficient (R), R^2, RMSE, and fractional bias (Table S5).

Table S5 | Summary statistics between the dust models

	**RF dust leave-1 year out**	**RF dust leave-1 station out**	**DREAM dust comparison**
**Pearson r**	0.54	0.52	0.49
**R^2**	0.29	0.27	0.24
**RMSE**	6.0	6.2	12.6
**MAE**	2.8	3.2	5.0
**Fractional Bias (FB)**	30.1	38.2	-109

At a daily resolution, the RF model shows moderate explanatory power (Figs. S2, S4) which is expected given the strong spatial and temporal variability of transported dust and the inherent stochastic nature of dust emissions and transport processes. Importantly, the RF model substantially outperforms the regional physical dust model DREAM, which was used as an input predictor (Fig. S2, Table S5). This comparison highlights the clear added value of the observationally constrained machine-learning framework in capturing dust variability at monitoring sites.

Furthermore, while the model performance at daily resolution is moderate, agreement improves substantially when data are aggregated to annual means, which are the focus of the trend analysis presented in the manuscript (Fig. S3). At this temporal scale, the RF

reconstruction reproduces the observed interannual variability more robustly, making it suitable for the analysis of decadal dust trends.

We also thank the reviewer for raising the important point regarding temporal generalization of the RF model. In addition to the leave-one-station-out cross-validation used to evaluate spatial transferability, we assessed the model's ability to generalize in time using a leave-one-year-out cross-validation. In this test, the model is trained on all years except one and evaluated on the withheld year, iterating across all available years within the training period with similar performance across all years (Table S6). The leave-one-year-out validation yields a correlation coefficient of R = 0.54, an RMSE of 6 μg m^{-3}, and a fractional bias of 30%. These results are comparable to the leave-one-station-out validation (R = 0.53, RMSE = 6 μg m^{-3}, fractional bias = 39%), indicating that the model exhibits similar generalization capability across both time and space, and that the results are not driven by year-specific overfitting.

Finally, the dust trends presented in the manuscript are derived from annual mean concentrations, which reduces short-term variability and enhances the robustness of the inferred temporal changes. The training period, cross-validation strategy, and treatment of the year 2012 (expanded in the next comment) have now been clarified in the revised Methods section.

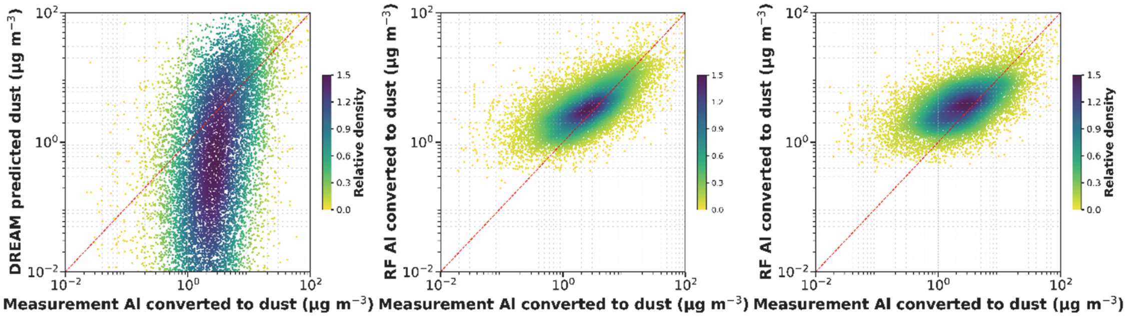

Fig S2 | Comparison between measured dust and DREAM (a), Random Forest leave-1-year-out (b) and leave-1-station-out (c). Statistical measures for this figure are shown in table S5.

The model performance section has been adjusted to reflect the additional validation analyses as follows:

Model performance

The model was optimized by hyperparameter testing, with the optimal parameters being n_estimators=150, max_depth=None, criterion='friedman_mse', bootstrap=True and random_state=66. The criterion for picking the optimal model was the average Pearson r value for all the sites from the leave one station out validation i.e. the model configuration that had the highest r across all sites. It was chosen over other absolute metrics such as RMSE, due to the measurements spanning many orders of magnitude (Figs S2, S3), which would lead to sites in the South with larger average concentrations, overpowering sites in the North. It's noteworthy that all configurations attempted yielded very similar results, indicating the method was insensitive to the hyperparameters.

A leave one year out validation i.e. removing an entire year from the training dataset and predicting it was also conducted yielding similar results to the leave one station out, indicating the model carries no inherent spatiotemporal biases (Table S5 & S6, Fig. S2).To further validate the model, and test its generalizability, we conducted an additional temporal validation for the year 2012 which was not included in the training of the model. In addition, these sites, with the exception of Athens and Finokalia were also not included in the training dataset, meaning that the model's spatial and temporal predictive abilities were tested at the same time. The year was not used as a predictor variable, preventing the model from learning or imposing artificial temporal trends. Model performance for the unused year 2012 is very similar to that of the other years used in training (Table S6, Fig. S14), reinforcing the model's robustness.

Model training is completed within less than 5 minutes, while producing dust fields for 10 years and for the entire domain takes 53 minutes on 60 processors (2 cores per processor).

2. What is the time series range used for RF model training—2012 to 2021? What time resolution did the authors employ? Some datasets may have lower time resolution; how did the authors handle differences in time resolution across datasets?

The RF model was trained using strictly daily observations (with the exception of XACT measurements that were in hourly resolution and subsequently aggregated to daily averages) from 2013–2021, while reconstructed daily dust concentrations cover the analysis period 2012–2021. The year 2012 therefore represents an out-of-sample prediction. Importantly, "year" was not included as a predictor variable, preventing the model from learning or imposing artificial temporal trends. Consequently, trends in reconstructed dust concentrations arise solely from variations in the meteorological and environmental predictors rather than from the model structure itself.

We have now included an additional validation of model performance for the year 2012 using newly collected observations. This independent evaluation shows performance consistent with that obtained for the other years in the dataset, with the description found in the Model performance section of the Methods (pasted above).

Table S6 | Summary performance statistics of the RF dust model for different years

Year Fold	# of datapoints	Pearson r	R^2	RMSE	MAE	Fractional Bias (FB)
**2012***	664	0.49	0.24	5.44	2.84	-0.62
**2013**	2678	0.48	0.24	4.76	2.61	23.30
**2014**	2697	0.50	0.25	6.71	3.18	20.58
**2015**	1903	0.51	0.26	6.30	3.00	17.59
**2016**	1285	0.58	0.33	10.11	3.79	31.09
**2017**	2163	0.58	0.33	5.75	2.69	30.56
**2018**	1860	0.58	0.33	6.27	3.11	24.35
**2019**	2679	0.52	0.27	4.95	2.57	34.49
**2020**	2137	0.60	0.36	5.14	2.35	21.06
**2021**	1066	0.53	0.28	6.14	3.21	29.89

*not included in final model.

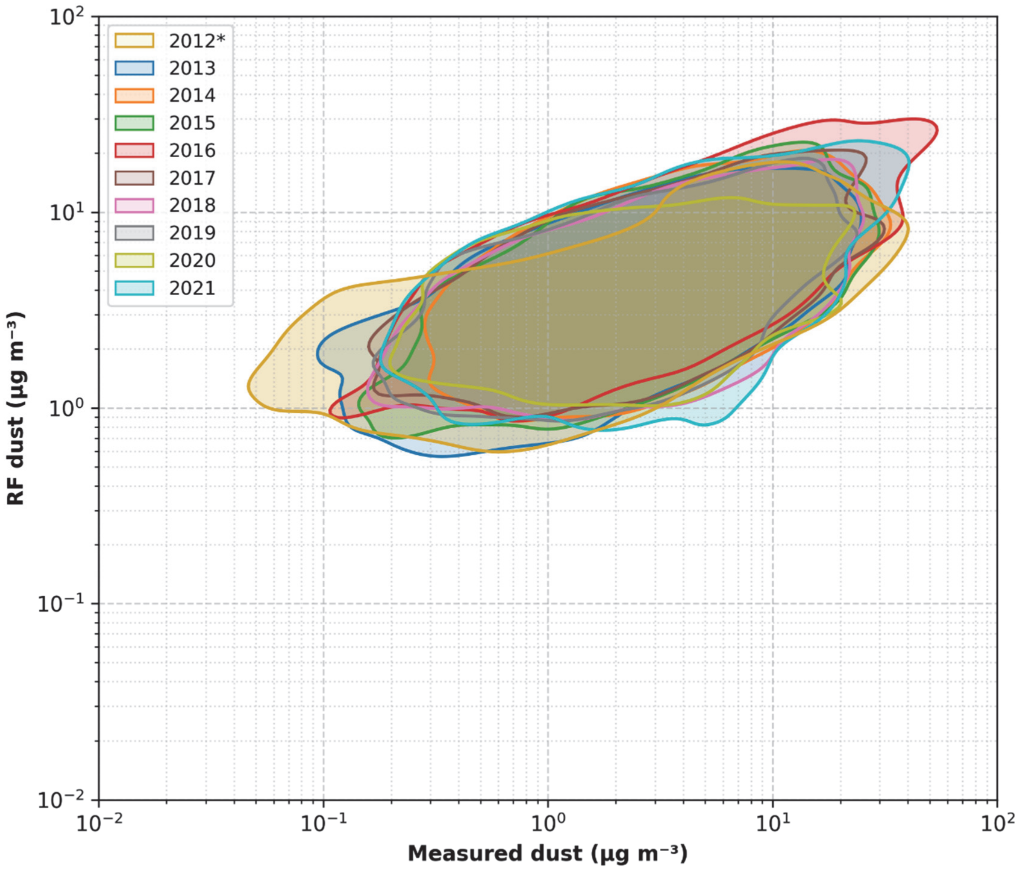

Fig S14 | Density plot for 90% of predictions, grouped by year, for the leave-1-year-out results (*2012 not included in the final model).

3. I still believe the authors should reconsider the manuscript's innovative points and highlights—should the focus lean more toward methodology or cognition? Integrating models of well-defined physical processes to enhance the interpretability of machine learning is a current hot research direction. However, the current exploration of this methodology clearly lacks sufficient depth, which is an aspect I find particularly intriguing. Additionally, have the authors compared their work to existing studies? Modeling and prediction of airborne particulate matter are now well-established fields. Does this manuscript's model outperform current approaches in capturing dust pollution events?

We thank the reviewer for this thoughtful comment. We agree that integrating physically grounded processes with data-driven approaches is an important and rapidly evolving direction.

In this work, the modeling framework plays a central role; however, the primary contribution is not the development of a new machine-learning methodology per se. Rather, the novelty lies in the construction and exploitation of what is, to our knowledge, the most comprehensive dataset to date of PM elemental composition, which we combine with physically based dust proxies from a transport model, satellite reanalysis, and meteorological variables to derive a spatially resolved reconstruction of daily mineral dust concentrations across Europe over a decade.

While machine-learning approaches for predicting total PM are indeed well established (especially given the widespread availability of total PM mass concentration measurements), far fewer studies have focused on reconstructing a specific PM component, particularly transported mineral dust, using such an extensive observational constraint. The limited availability of comprehensive elemental composition data has, until now, been the key bottleneck for this type of analysis.

We also note that current chemical transport models (e.g., SKIRON, MONARCH, CHIMERE, DREAM) exhibit known limitations in capturing the magnitude and variability of dust transport over Europe. As a benchmark, we compared the RF model developed here with the regional physical dust model DREAM. The RF model shows substantially improved agreement with surface observations relative to DREAM (Fig. S2, Table S5). This positioning, along with relevant comparisons to existing studies, is discussed in the manuscript (Introduction, lines 150–166).

Finally, an additional novelty is the integration of these reconstructed concentrations with atmospheric drivers and Alpine ice-core records, allowing us to understand both short-term variability and longer-term trends in European dust.

To improve clarity, we have revised the abstract and corresponding sections of the manuscript to better emphasize these points and more clearly articulate the scope and innovation of the study.

.

Mineral desert dust is a major contributor to total atmospheric particulate matter[1]. Desert dust outbreaks degrade air quality and can pose adverse health effects[2], including asthma exacerbation[3,4] and increased mortality[5]. At some European locations, there has been a rise in the intensity and frequency of transported dust outbreaks from deserts in recent decades[6-10]. However, it remains unclear whether this rise is consistent across Europe and whether desertification and aridity or shifts in atmospheric circulation are the main drivers behind this rise. Here we compile a database of daily dust metal concentrations from European sites, establishing robust elemental ratios for transported dust. Leveraging this database, we develop a machine-learning model to estimate daily PM10 (particulate matter smaller than 10 µm) dust concentrations from 2012 to 2021, ranging from 2.09±1.05 µg m^{-3} across Northern and Central Europe to 5.28±2.65 µg m^{-3} across the South. In southern Europe, residents are exposed to transported dust events averaging 9.68±4.85 µg m^{-3}, linked to a 0.67±0.02% rise in daily mortality. Intensified dust intrusions over the past decade are linked to shifts in atmospheric circulation. Data from an Alpine ice-core record shows a 110% increase in dust concentrations since preindustrial times, mostly associated with North African desertification. As climate change accelerates land degradation and affects weather patterns, worsening dust pollution may pose increasing risks to public health and air quality goals.

4. Certain intuitive verifications remain necessary. For example, in Fig. S7, is there any spatial correlation analysis based on satellite data or observational and research findings?

We thank the reviewer for this comment. The episode used in this study shown in Figure S7 was one of the most severe dust storms to hit Europe in recent years and therefore chosen as a validation test of the model's ability to predict trajectories of dust intrusions. Due to the lack of pure satellite imagery/data over Europe for the duration of the event apart from the MODIS unprocessed snapshots shown in Peshev et al. (2021), a direct spatial correlation analysis is not possible.

In the initial version of the manuscript, we had included the fields of the DREAM model as an additional validation test of the predicted pattern, that were later removed during the review process in accordance with reviewers' requests. Satellite reanalysis data are also available, and as such can be used for intuitive verification. We created analogous plots based on dust optical depth at 550nm (duaod550) which is available as a reanalysis product from the Copernicus Atmosphere Monitoring Service (CAMS), blending measurements, satellite imagery and model results leading to fields more accurate than the sum of its parts. We note that CAMS dust AOD integrates the entire dust column while we model surface dust concentrations. Thus, differences are to be expected to the RF surface dust concentrations we model in the present study. Nevertheless, similar features can be observed leading up to the middle of the observed dust episode (Figs S7). After that, the main dust plume according to

CAMS dust AOD is above Greece while RF transported dust resides over the Balkans. It is noteworthy that the RF model correctly predicts lower concentrations over the Adriatic for the duration of the event, despite optical depth being elevated, indicative of the uplift of the plume when hitting the Apennine Mountains, a feature mirrored in the DREAM model results as well, which further adds credence to the model's ability to capture the fine details of the event.

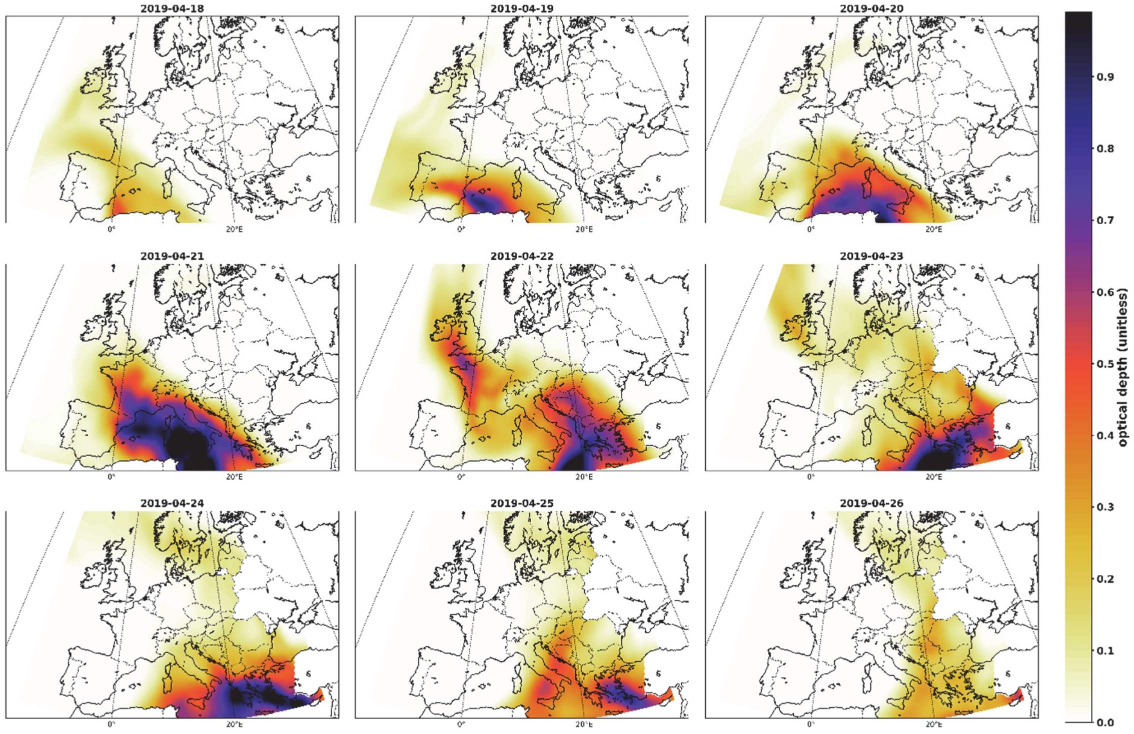

Dust optical depth at 550 nm (CAMS reanalysis product) showing the simulated trajectory of Saharan dust during the extreme event affecting the Balkans, as reported by Peshev et al. (2021), for the period 18 April 2019 (one day before the episode's onset) to 26 April 2019 (episode end).

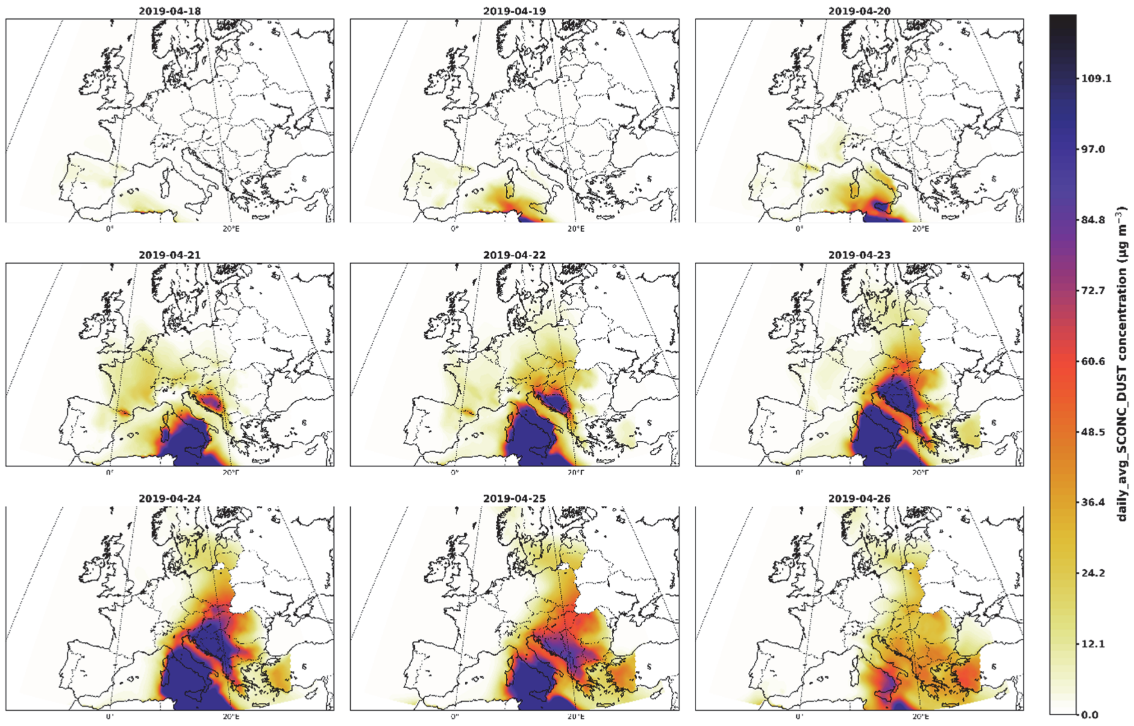

DREAM predicted dust concentrations (μg m^{-3}) showing the simulated transport trajectory of Saharan dust during the extreme event affecting the Balkans, as reported by Peshev et al. (2021), for the period 18 April 2019 (one day before the episode's onset) to 26 April 2019 (episode end).

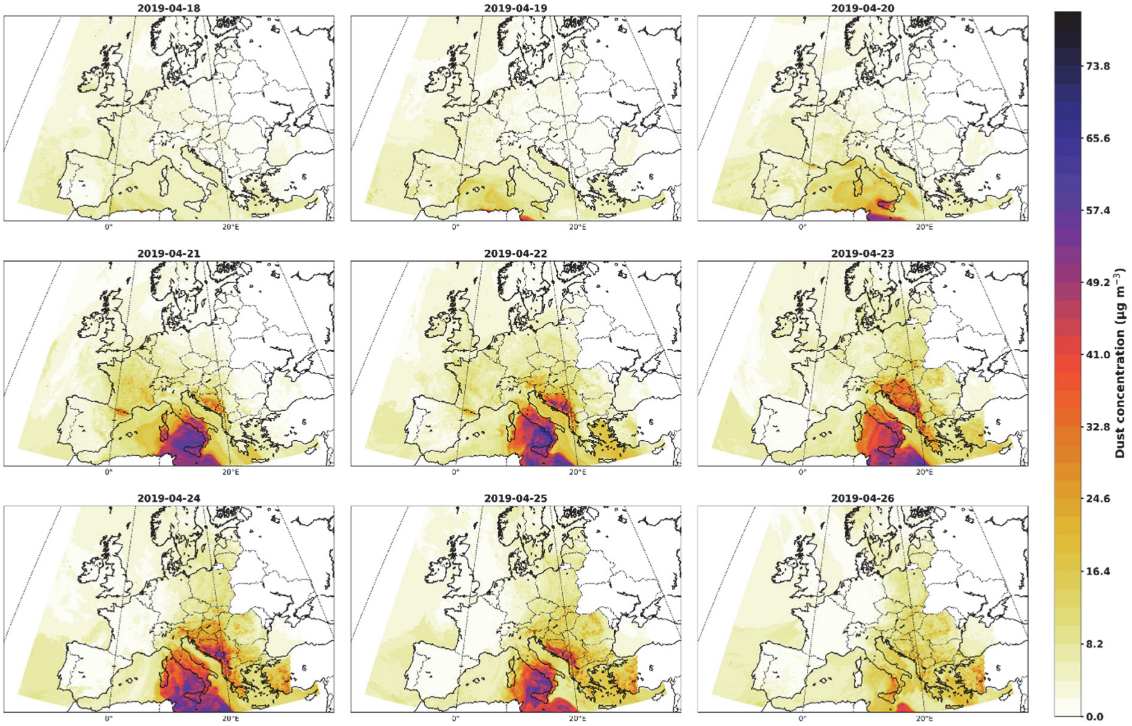

Fig S7 | Random forest–predicted dust concentrations (μg m^{-3}) showing the simulated transport trajectory of Saharan dust during the extreme event affecting the Balkans, as reported by Peshev et al. (2021), for the period 18 April 2019 (one day before the episode's onset) to 26 April 2019 (episode end).
